# High expression of *RUNX1* is associated with poorer outcomes in cytogenetically normal acute myeloid leukemia

**DOI:** 10.18632/oncotarget.7489

**Published:** 2016-02-19

**Authors:** Lin Fu, Huaping Fu, Lei Tian, Keman Xu, Kai Hu, Jing Wang, Jijun Wang, Hongmei Jing, Jinlong Shi, Xiaoyan Ke

**Affiliations:** ^1^ Department of Hematology and Lymphoma Research Center, Peking University, Third Hospital, Beijing, 100191, China; ^2^ Medical Engineering Support Center, Chinese PLA General Hospital, Beijing, 100853, China; ^3^ Department of Nuclear Medicine, Chinese PLA General Hospital, Beijing, 100853, China; ^4^ College of Medical Laboratory Science and Technology, Harbin Medical University, Daqing, 163319, China

**Keywords:** RUNX1, prognostic biomarker, CN-AML

## Abstract

Depending on its expression level, *RUNX1* can act as a tumor promoter or suppressor in hematological malignancies. The clinical impact of *RUNX1* expression in cytogenetically normal acute myeloid leukemia (CN-AML) remained unknown, however. We evaluated the prognostic significance of *RUNX1* expression using several public microarray datasets. In the testing group (*n* = 157), high *RUNX1* expression (*RUNX1*^high^) was associated with poorer overall survival (OS; *P* = 0.0025) and event-free survival (EFS; *P* = 0.0025) than low *RUNX1* expression (*RUNX1*^low^). In addition, the prognostic significance of *RUNX1* was confirmed using European Leukemia Net (ELN) genetic categories and multivariable analysis, which was further validated using a second independent CN-AML cohort (*n* = 162, OS; *P* = 0.03953). To better understand the mechanisms of *RUNX1*, we investigated genome-wide gene/microRNAs expression signatures and cell signaling pathways associated with *RUNX1* expression status. Several known oncogenes/oncogenic microRNAs and cell signaling pathways were all up-regulated, while some anti-oncogenes and molecules of immune activation were down-regulated in *RUNX1*^high^ CN-AML patients. These findings suggest *RUNX1*^high^ is a prognostic biomarker of unfavorable outcome in CN-AML, which is supported by the distinctive gene/microRNA signatures and cell signaling pathways.

## INTRODUCTION

Cytogenetically normal acute myeloid leukemia (CN-AML) comprises the largest percentage of primary AML cases [1]. Although the leukemic blasts do not include detectable chromosome abnormalities in CN-AML patients, they nonetheless hide mutations and aberrantly expressed proteins [2] and microRNAs [3], which are potentially prognostic. Among them, *NPM1* [4] and double *CEBPA* [5] mutations are associated with better outcomes, while *FLT3*-ITD [6] and *RUNX1* mutation [7] are associated with poorer ones. High expression of *WT1* [8], *BAALC* [9], *ERG* [9], *MN1* [10], *DNMT3B* [11], *TCF4* [12], *ITPR2* [13] and *MAPKBP1* [14] and low expression of *LEF1* [15] are also associated with a poor prognosis, as is high expression of *miR*-155 [16] and *miR-188-5p* [17] and low expression of *let-7a-2-3p* [17].

*RUNX1* belongs to the Runt-related transcription factor (*RUNX*) family, which plays a crucial role in normal hematopoiesis, and its abnormal expression is frequently seen in various tumors [18, 19]. In several AML subtypes, for example, chromosomal translocations involving *RUNX1* lead to fusion gene formation, *RUNX1-RUNX1T1* being the most common type [20]. In addition, *RUNX1* mutation leads to a poor outcome in CN-AML [7], and high expression of *RUNX1* correlates with a poor prognosis in breast cancer [21]. Notably, although early studies suggested *RUNX1* acts as a tumor suppressor gene in AML [22], it is now understood that *RUNX1* functions as an oncogene necessary to sustain AML [23–26]. These findings suggest that the prognostic impact of *RUNX1* in CN-AML depends on its expression level.

We found that *RUNX1* is more strongly expressed in CN-AML patients than in normal bone marrow (NBM), but also was an unfavorable prognostic factor in two large, independent groups of patients with CN-AML. In addition, we provide the first report that *RUNX1* expression is linked to particular molecular and clinical characteristics. In order to cast light on the function of *RUNX1*, we also explored *RUNX1*-associated genes, microRNAs and important cell signaling pathways.

## RESULTS

### Expression of *RUNX1* in CN-AML BM and NBM

**Figure 1:**
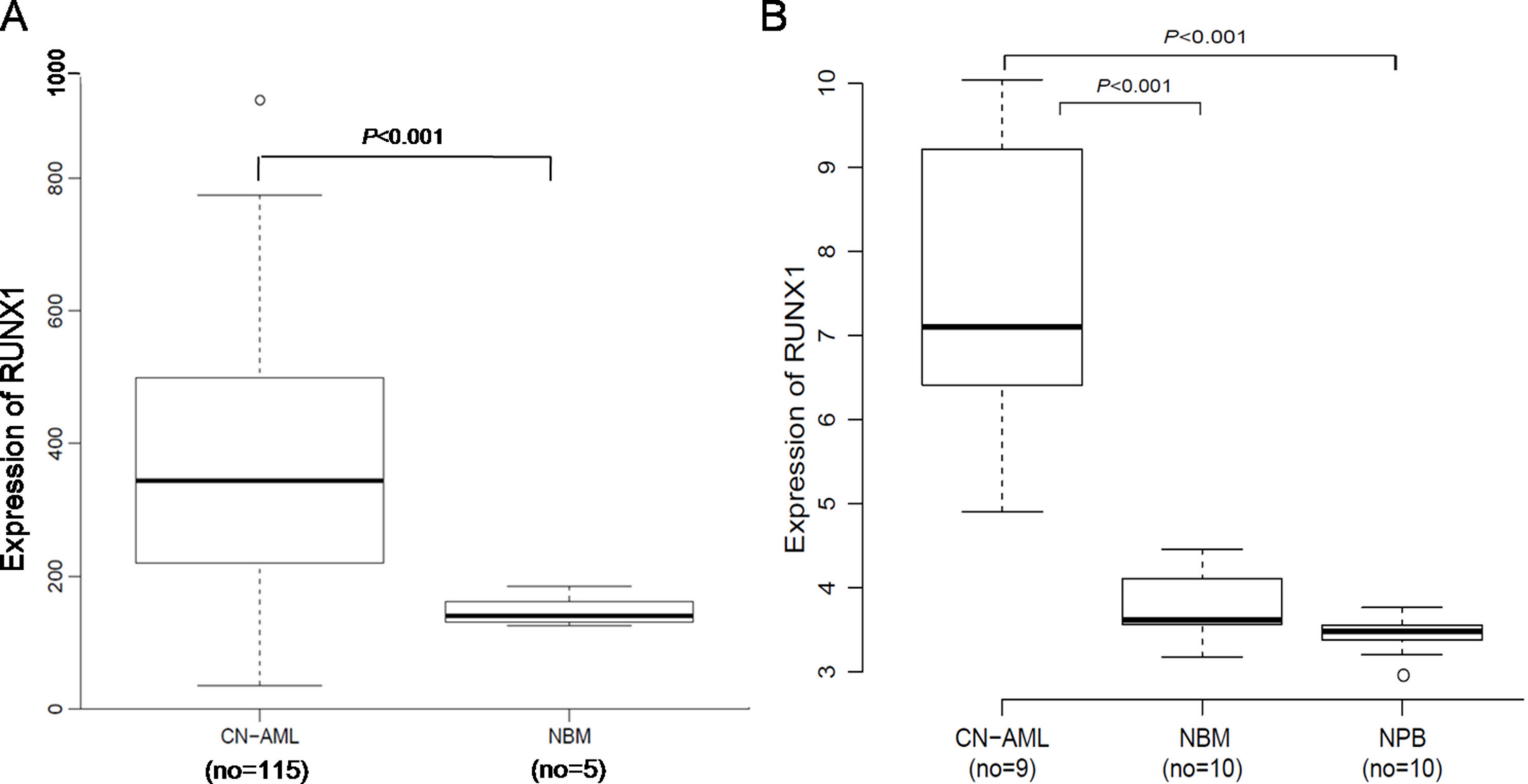
Expression of *RUNX1* in CN-AML patients and NBM (**A**) Box plot of *RUNX1* expression in CN-AML patients (*n* = 116) and NBM samples (*n* = 5). (**B**) Box plot of *RUNX1* expression in CN-AML patients (*n* = 9, including 2 BM and 7 PB samples), NBM samples (*n* = 10) and NPB samples (*n* = 10).

A microarray dataset that included 116 CN-AML samples and 5 NBM samples (GEO accession number *GSE1159*) was used for the expression analysis [27]. *RUNX1* expression was markedly higher in the CN-AML than NBM samples (*P* < 0.001) (Figure 1A). The overexpression of *RUNX1* in CN-AML was further validated using other microarray data, which included 9 CN-AML vs. 10 NBM (*P* < 0.001) and 9 CN-AML vs. 10 normal peripheral blood (NPB) (*P* < 0.001). The 9 CN-AML samples consisted of 2 BM and 7 PB samples, GEO accession number *GSE9476*) [28] (Figure 1B). These findings show that *RUNX1* overexpression is widespread among CN-AML patients, and is easy to monitor.

### Characteristics of patients in the *RUNX1*^high^ and *RUNX1*^low^ expression groups

Among the 157 CN-AML patients tested, the *RUNX1*^high^ group contained significantly more patients with FAB M2 than the *RUNX1*^low^ (*P* = 0.001). The *RUNX1*^high^ patients were also more likely than *RUNX1*^low^ patients to carry *FLT3*-ITD and no double *CEBPA* mutations (*P* < 0.001, *P* = 0.003). We found no link between *RUNX1* expression and other gene mutations, but *RUNX1*^high^ patients with CN-AML were more likely to highly express *ERG*, *WT1, DNMT3B, TCF4, MIR155HG, ITPR2* and *MAPKBP1* (*P* < 0.001, *P* < 0.001, *P* < 0.001, *P* < 0.001, *P* = 0.01, *P* < 0.001, and *P*< 0.001, respectively) (Table 1, Supplementary Figure 1).

**Table 1:** Patients' characteristics in the testing group of 157 CN-AML patients according to RUNX1 expression levels

Variable	*RUNX1*^high^, *n* = 78	*RUNX1*^low^, *n* = 79	*P*
Median age. y (range)	50 (18~77)	48 (16~75)	0.325
Female sex, no.(%)	40	33	0.27
FAB subtype, no.			
M0	1	2	1
M1	25	20	0.38
M2	24	8	0.001
M3	1	0	1
M4	12	12	0.5
M5	14	25	0.06
M6	0	1	1
Other	1	11	0.005
*FLT3*-ITD, no.	45	21	< 0.001
*FLT3*-TKD, no.	8	12	0.47
*NPM1*, mutated, no.	46	36	0.11
Double *CEBPA*, mutated, no.	2	14	0.003
*N-RAS*, mutated, no.	4	9	0.25
*K-RAS*, mutated, no.	0	1	1
*IDH1*, mutated, no.	58	59	0.64
*IDH2*, mutated, no.	59	64	0.49
ELN genetic group, no.			
Favorable	13	22	0.125
Intermediate-I	65	57	0.12
High *ERG*, no.	51	27	< 0.001
High *BAALC*, no.	43	35	0.2
High *LEF1*, no.	33	45	0.08
High *MN1*, no.	39	39	1
High *WT1*, no.	60	18	< 0.001
High *DNMT3B*, no.	58	20	< 0.001
High *TCF4*, no.	53	25	< 0.001
High *MIR155HG*, no.	47	31	0.01
High *ITPR2*, no.	54	24	< 0.001
High *MAPKBP1*, no.	54	24	< 0.001

**FAB**, French-American-British classification; **ITD**, internal tandem duplication; **TKD**, tyrosine kinase domain; **ELN**, European Leukemia Net.

High *ERG, BAALC, LEF1, MN1, WT1, DNMT3B, TCF4, MIR155HG, ITPR2* and *MAPKBP1* expression were defined as an expression level above the median of all samples, respectively.

### *RUNX1*^high^ is associated with poor outcomes

**Figure 2:**
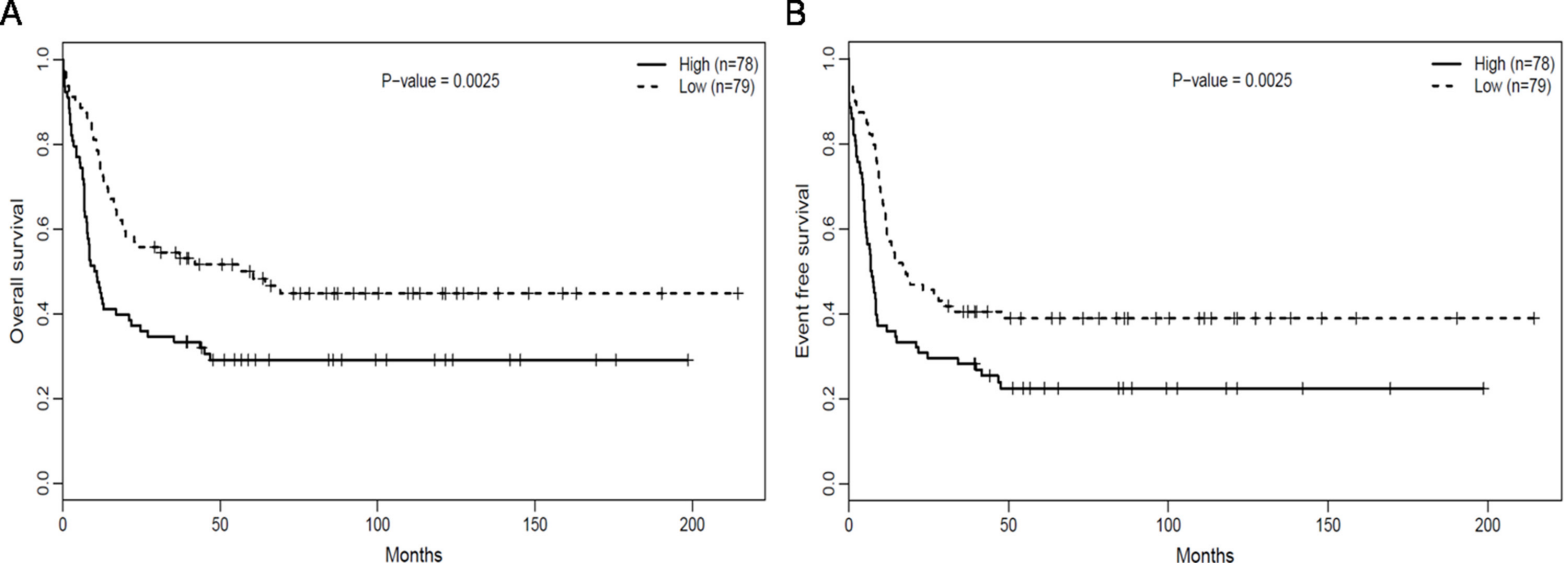
*RUNX1*^high^ is associated with poorer outcomes (**A**) OS and (**B**) EFS in the testing group of 157 CN-AML patients.

The median overall survival (OS) and event-free survival (EFS) in the *RUNX1*^high^ group were obviously poorer than that of *RUNX1*^low^group (*P* = 0.009, *P* = 0.011, respectively, Table 2). This was confirmed comparison using the Log-rank test, which also showed that OS (Figure 2B, *P* = 0.0025) and EFS (Figure 2A, *P* = 0.0025) were clearly poorer in the *RUNX1*^high^ than *RUNX1*^low^ group.

**Table 2:** Survival according to *RUNX1* expression in the testing group of 157 CN-AML patients

Outcome	All patients, *n* = 157			ELN Favorable category			ELN Intermediate-I category		
	*RUNX1*^high^, *n* = 78	*RUNX1*^low^, *n* = 79	*P*	*RUNX1*^high^, *n* = 17	*RUNX1*^low^, *n* = 18	*P*	*RUNX1*^high^, *n* = 65	*RUNX1*^low^, *n* = 57	*P*
OS									
Median OS, m	10.46 (0.07–198.7)	37.03 (0.13–214.5)	0.009	58.91 (0.59–169.5)	38.34 (0.3–214.5)	0.65	8.41 (0.07–198.7)	35.91 (0.13–190.3)	0.002
Estimated OS at 3 y. % (95% CI)	0.33 (0.24–0.46)	0.54 (0.45–0.67)	0.01	0.65 (0.46–0.92)	0.56 (0.37–0.84)	0.73	0.25 (0.16–0.38)	0.54 (0.43–0.68)	0.007
EFS									
Median EFS, m	7.1 (0.03–198.7)	17.81 (0.03–214.5)	0.011	39.82 (0.03–169.5)	33.03 (0.03–214.5)	0.36	6.57 (0.03–198.7)	15.54 (0.03–190.3)	0.004
Estimated EFS at 3 y. % (95% CI)	0.28 (0.2–0.4)	0.41 (0.31–0.53)	0.005	0.53 (0.34–0.83)	0.56 (0.37–0.84)	0.17	0.2 (0.12–0.33)	0.38 (0.27–0.52)	0.03

CI, confidence interval.

### Association of *RUNX1* expression with prognostic significance in ELN genetic groups

**Figure 3:**
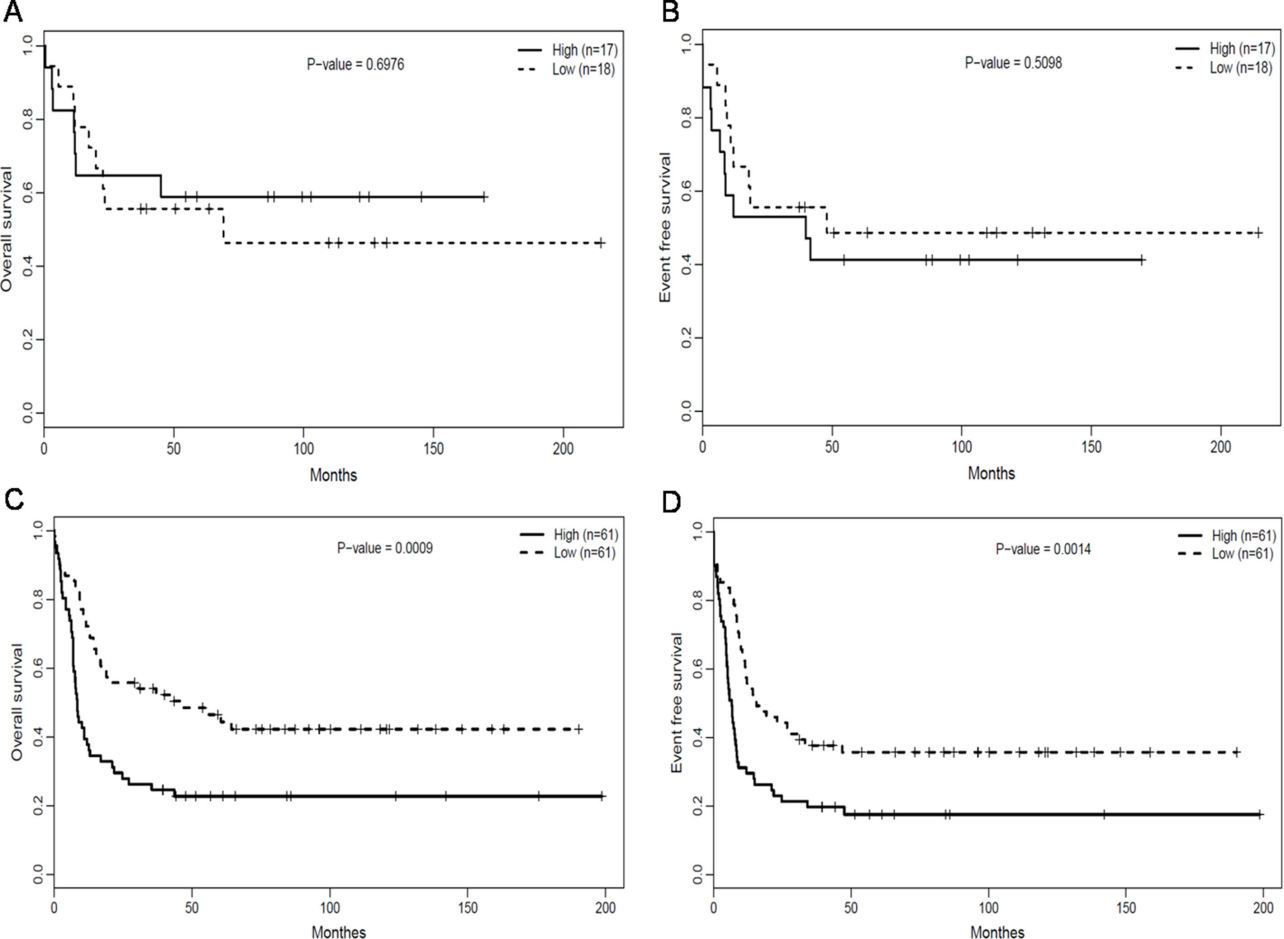
Association of *RUNX1* expression with the prognostic significance in ELN genetic groups (**A**) OS and (**B**) EFS of CN-AML patients in the ELN favorable genetic group. (**C**) OS and (**D**) EFS of CN-AML patients in the ELN intermediate-I genetic group.

We assessed the association between *RUNX1* expression and prognostic significance separately within the European Leukemia NET (ELN) favorable and Intermediate-I genetic groups. Within the ELN favorable group (*n* = 35), there was no obvious difference in OS (Figure 3A, *P* = 0.6976) and EFS (Figure 3B, *P* = 0.5098) between the *RUNX1*^high^ and *RUNX1*^low^group. In the ELN Intermediate-I group (*n* = 122), however, the *RUNX1*^high^ group had poorer OS (Figure 3C, *P* = 0.0009) and EFS (Figure 3D, *P* = 0.0014) than the *RUNX1*^low^ group. The median OS, EFS and estimated survival in the ELN Intermediate-I group (*n* = 122) also obviously differed between the *RUNX1*^high^ and *RUNX1*^low^groups (Table 2).

### *RUNX1* expression is associated with poorer OS and EFS in multivariable analyses

ELN segregated CN-AML patients based on presence of *FLT3*-ITD, mutations of *NPM1* and *CEBPA.* After adjusting for the impact of these known risk factors, we performed multivariable analyses to confirm the prognostic significance of *RUNX1* expression. In a multivariable model, the *RUNX1*^high^ group had a poorer OS (*P* = 0.04, Table 3). The other factors associated with poor OS were *NPM1* wild-type and *FLT3*-ITD. The *RUNX1*^high^ group also had a poorer EFS in a multivariable model (*P* = 0.02, Table 3). The other factors associated with poor EFS were *NPM1* wild-type and *FLT3*-ITD.

**Table 3:** Multivariable analysis with OS and EFS in the testing group of 157 CN-AML patients

Variable	OS, *n* = 157		EFS, *n* = 157	
	HR (95% CI)	*P*	HR (95% CI)	*P*
*RUNX1* expression, high VS low	1.56 (1.01–2.41)	0.04	1.65 (1.10–2.48)	0.02
Age, per 10-y increase	1.13 (0.98–1.32)	0.09	1.05 (0.92–1.21)	0.47
Sex male VS female	0.82 (0.54–1.23)	0.33	0.99 (0.67–1.46)	0.96
NPM1, mutated VS wild type	0.51 (0.32–0.81)	0.005	0.53 (0.34–0.83)	0.005
FLT3-ITD, mutated VS wild type	1.98 (1.25–3.14)	0.003	1.85 (1.20–2.85)	0.005
CEBPA, mutated VS wild type	0.71 (0.38–1.35)	0.3	0.78 (0.43–1.41)	0.41

**HR**, hazards ratio; **CI**, confidence interval.

### Validation in a patient group of 162 CN-AML samples

We also studied a group of 162 previously untreated CN-AML patients. The *RUNX1*^high^ group contained significantly more patients with FAB M1 than the *RUNX1*^low^ group (*P* = 0.0014). In addition, *RUNX1*^high^ patients with CN-AML were more likely to have higher expression of *ERG, BAALC, WT1, DNMT3B, TCF4, ITPR2* and *MAPKBP1* (*P* < 0.001, *P* = 0.028, *P* < 0.001, *P* < 0.001, *P* < 0.001, *P* < 0.001, and *P* < 0.001, respectively) and low *LEF1* (*P* < 0.001) compared with *RUNX1*^low^ patients (Supplementary Table 1). In addition, *RUNX1*^high^ patients showed a significant poor OS (*n* = 81 *vs n* = 81, *P* = 0.04; Supplementary Figure 2) than *RUNX*1^low^ patients.

### Genome-wide gene expression profiles associated with *RUNX1* expression

**Figure 4:**
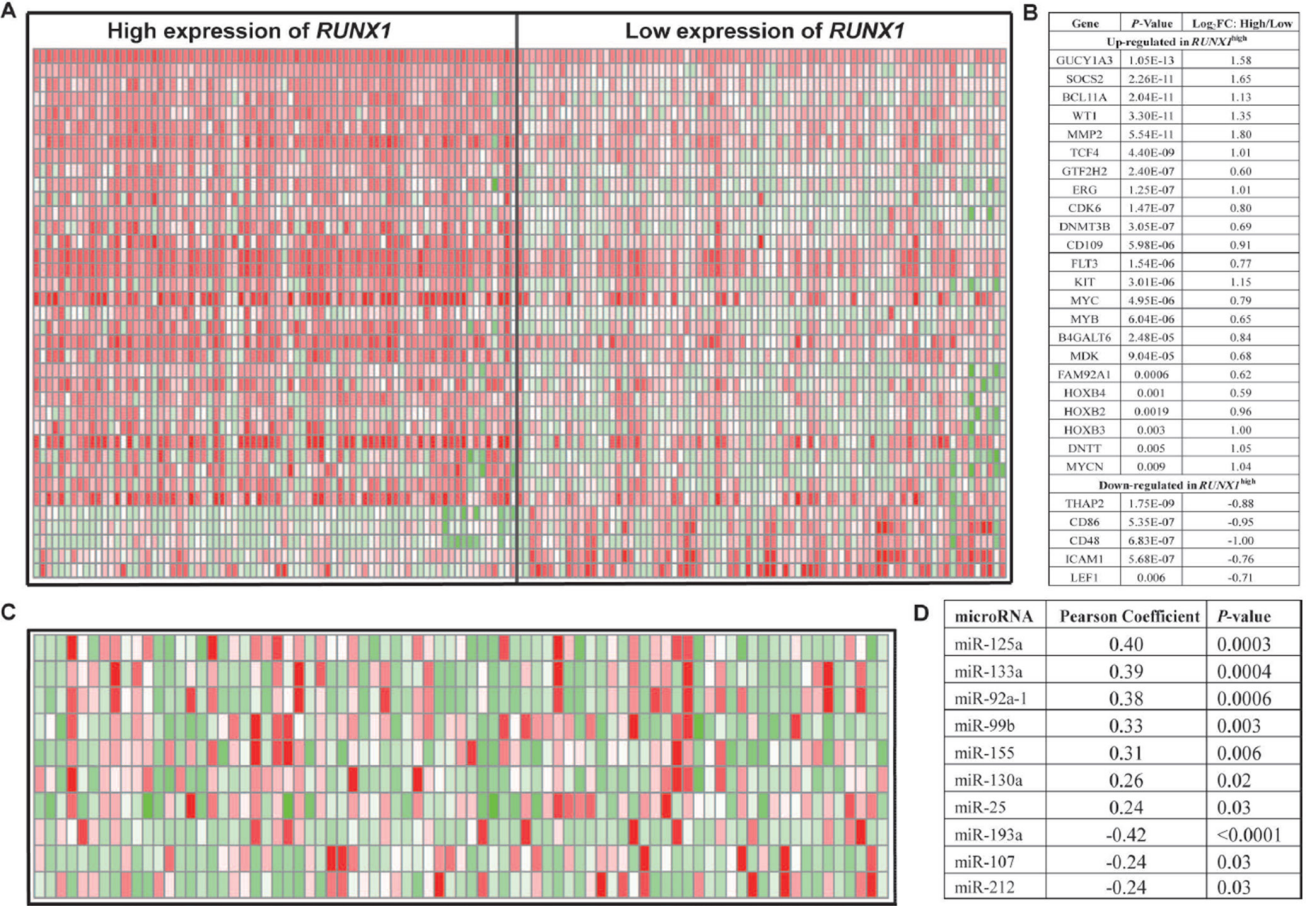
Genome-wide gene/microRNA-expression profiles associated with *RUNX1* expression (**A**) Expression heat map of associated genes (**B**) The list of associated genes. (**C**) Expression heat map of associated microRNAs. (**D**) The list of associated microRNAs.

To further evaluate the role of *RUNX1* in CN-AML, we using microarray analysis to determine *RUNX1*-associated gene expression profiles. We identified 578 up-regulated genes and 727 down-regulated genes that were significantly associated with *RUNX1*^high^ (Supplementary Table 2). The up-regulated genes included some of those previously found to be involved in leukemogenesis, including *CDK6*, which encodes a cyclin kinase; *MYCN*, *MYB* and *MYC*; members of the *HOXB* gene family (*HOXB2*, *HOXB3*, and *HOXB4*), which encode transcription factors [29]; and *c-KIT* and *FLT3*, which encode tyrosine kinases. Several independent unfavorable prognostic factors in CN-AML were also up-regulated, including *ERG*, *WT1*, *TCF4* and *DNMT3B*. Also up-regulated were *B4GALT6*, which is expressed in less differentiated precursors [30]; *SOCS2*, which is predictive of a poor outcome in pediatric AML [31]; *BCL11A* and *GUCY1A3*, which are down-regulated in low *ERG* expressers [9]; *GTF2H*2/ABCC5, which correlates with chemotherapy resistance in non-small cell lung cancer [32]; *DNTT*, which is expressed in early lymphoid precursors [33]; *CD109*, which is overexpressed in early hematopoietic stem cells [34]; *FAM92A1*, which enhances cell growth during renal carcinogenesis [35]; and *MMP2*, which promotes lung cancer metastasis [36]. The down-regulated genes included thanatos-associated protein 2 (*THAP2*) and *CD48*, *CD86* and *ICAM1*, all of which are involved in immune function. *LEF1*, an independent favorable prognostic factor in CN-AML, was also down-regulated (Figure 4A and 4B). These results provided further evidence for the prognostic correlation described above.

### Genome-wide microRNA profiles associated with *RUNX1* expression

A genome-wide analysis of microRNA profiles revealed that 108 microRNAs were significantly associated with *RUNX1* expression (*P* < 0.05) (Supplementary Table 3). *RUNX1*^high^ was positively associated with *miR-155, miR-125a, miR-99b, miR-133a, miR-130a, miR-25* and *miR-92a-1*. *MiR-155* was previously found to function as an oncogene in CN-AML [16]. *MiR-125a* and *miR-99b* were highly expressed in hematopoietic stem cells [37]. *MiR-133a* was up-regulated in CN-AML along with *IDH2* codon R172K [38]. *MiR-130a* associated with strong expression of *WT1*, which was consistent with the gene-expression profiles [39]. *MiR-25* increases induction of somatic cells into induced pluripotent stem cells [40]. *MiR-92a-1* arouses erythroleukemia through down-regulation of *p53* [41]. Notably, *miR-193a, miR-1*07 and *miR-212* were all down-regulated. We previously found that *miR-193a* enhanced expression of *c-kit [42]*, which is also consistent with the observed gene-expression profiles. *MiR-107* targets *NFIX*, which competes with *CEBPA* for binding to the promoter of *miR-223*, impaired granulocytic differentiation [43, 44]. *MiR-212* expression is favorable for survival among molecularly and cytogenetically heterogeneous AMLs [45] (Figure 4C and 4D).

### Genome-wide methylation profiling associated with *RUNX1* expression

It has been suggested that control of gene expression through methylation of the gene promoter or body plays a pivotal role in determining the behavior of cancer cells [46, 47]. Moreover, gene promoter methylation can be predictive of clinical outcome in AML patients [48, 49]. Because *RUNX1* expression correlated positively with *DNMT3B* expression, we compared the patterns of gene methylation in *RUNX1*^high^ (*n* = 37) and *RUNX1*^low^ (*n* = 37) CN-AML from TCGA [50]. However, we found no significant differences in patterns of *RUNX1* methylation in any of these analyses (Supplementary Figure 3A and 3B).

### Cell signaling pathways associated with *RUNX1* expression

We used MSigDB [51] to evaluate the cell signaling pathways underlying the biological features associated with *RUNX1*. Signaling pathways involved in *DNA_REPLICATION, RNA_POLYMERASE* and *CELL_CYCLE* were significantly up-regulated, while *NATURAL_KILLER_CELL_MEDIATED_CYTOTOXICI TY*, *ANTIGEN_PROCESSING_AND_PRESENTATION* and *APOPTOSIS* were down-regulated (Table 4). These findings were consistent with the above-noted dysregulated genes involved in the development of CN-AML.

**Table 4:** Cell signalling pathways associated with *RUNX1* expression levels

Pathway name	According to high expression of *RUNX1*	
	Regulation	*P*
KEGG_DNA_REPLICATION	Up	0.00424
KEGG_RNA_POLYMERASE	Up	0.01575
KEGG_CELL_CYCLE	Up	0.02204
KEGG_NATURAL_KILLER_CELL_MEDIATED_CYTOTOXICITY	Down	0.00000
KEGG_ANTIGEN_PROCESSING_AND_PRESENTATION	Down	0.00000
KEGG_APOPTOSIS	Down	0.00217

## DISCUSSION

CN-AML is the largest cytogenetic subset in AML patients and lacks sensitive prognostic biomarkers, so identification of universal prognostic biomarkers is a very important field in CN-AML research. *RUNX1* plays a crucial role in the development of normal hematopoiesis. Traditionally, loss of *RUNX1* leads to impaired differentiation and is followed by leukemia development [52]. However, several recent studies found that *RUNX1* plays a prosurvival role by supporting leukemia cell proliferation [23–26]. Based on earlier studies of *RUNX1*, the following conclusions can be made: 1) *RUNX1* plays an important dual role in myeloid leukemogenesis, depending on the level of its expression; 2) normal expression of *RUNX1* works as a tumor suppressor, inhibiting cell proliferation and promoting differentiation of hematopoietic progenitor cells; 3) Partial deactivation of *RUNX1* leads to amplification of myeloid progenitors and subsequent development of AML; and 4) further reduction of *RUNX1* expression causes cell cycle arrest and cell death [23–26].

Extending the studies outlined above, ours is the first study to show the prognostic relevance of *RUNX1* expression in CN-AML patients and that *RUNX1*^high^ is associated with poorer OS and EFS in CN-AML patients. *RUNX1* is up-regulated in CN-AML patients compared with NBM. In our study, the *RUNX1*^high^ group contained significantly more patients from the M1 (validating group) and M2 (testing group) FAB subgroups than did the *RUNX1*^low^ group, which suggests leukemic cells from *RUNX1*^high^ patients derive from relatively more immature cells. In addition, we found that *RUNX1*^high^ was associated with *FLT3*-ITD, non double *CEBPA* mutation and higher *ERG, WT1, DNMT3B, TCF4, MIR155HG, ITPR2, MAPKBP1* expression, all of which are unfavorable molecular characteristics in CN-AML patients. Furthermore, the association of *RUNX1*^high^ with poorer OS and EFS was confirmed in multivariable analyses adjusting for the most important clinical and molecular prognosticators in CN-AML patients. *RUNX1*^high^ was associated with wild-type *NPM1* and *FLT3*-ITD, both of which are unfavorable molecular characteristics in CN-AML patients. These results suggest *RUNX1*^high^ may be a surrogate marker for other unfavorable mutations. Our results also suggest that the prognostic impact of *RUNX1* expression is most pronounced in the ELN intermediate-I genetic group, and thus *RUNX1* expression may be used to further refine risk stratification for these patients.

The mechanisms underlying the association between *RUNX1*^high^ and poorer treatment outcomes are unclear. In our present study, we analyzed gene and microRNA expression, DNA methylation profiles, and cell signaling pathways to identify biological mechanisms associated with *RUNX1* expression in CN-AML patients. Gene sets related to cell proliferation and cell cycle regulation, particularly *c-KIT, FLT3*, *MYCN*, *MYB*, *MYC* and *CDK6*, were up-regulated in the CN-AML patients with *RUNX1*^high^, while gene sets related to independent unfavorable prognostic factors, particularly *ERG, WT1* and *DNMT3B,* were also up-regulated, and gene sets related to apoptosis, immune activation of NK cell and independent superior prognostic factor were down-regulated. Acting collectively, these features may lead to CN-AML.

The *RUNX1*-associated microRNA profile was also noteworthy, as it included the *miR-155* and *miR-130a* families, which were expressed with *RUNX1*. The up-regulation of *miR-155* was associated with an unfavorable clinical outcome independently in CN-AML. *MiR-130a* was associated with high expression of *WT1*. The down-regulation of *miR-193a* was associated with high expression of *c-KIT*. This new finding of *RUNX1*-associated alterations in microRNA expression may contribute to leukemogenesis.

Current studies suggest that hypermethylation of the gene promoter and hypomethylation of gene body contribute to the development of tumors [46, 47]. However, we found no significant association between *RUNX1* expression and the methylation levels in its promoter region or gene body. Therefore, although *RUNX1*^high^ is a predictive marker poorer outcome in CN-AML, epigenetic regulation may not play an important role in *RUNX1*^high^ CN-AML development.

Several important signaling pathways that promote cell proliferation in tumors or contribute to leukemogenesis, including *DNA_REPLICATION, RNA_POLYMERASE* and *CELL_CYCLE* were up-regulated, and *NATURAL_KILLER_CELL_MEDIATED_CYTOTO XICITY*, *ANTIGEN_PROCESSING_AND_PRESENTA TION*, all lead to immune escape, while *APOPTOSIS* was down-regulated in the *RUNX1*^high^ CN-AML. These changes may contribute to a poor outcome.

In summary, our study is the first to provide evidence that *RUNX1*^high^ is associated with poorer outcomes in CN-AML patients, even after adjusting for known molecular risk factors. In the validating group, earlier findings demonstrated that the microarray expression data for *LEF1* was in good agreement with quantitative real time PCR (qPCR) [15]. This shows to some degree the consistency and validity of the microarray expression data. Because *RUNX1* is widely expressed at a higher level in CN-AML patients than NBM, *RUNX1* expression can be easily measured. This may therefore be a valuable new marker for risk stratification of CN-AML patients. Moreover, our gene/microRNA expression data and cell signaling pathways from tested CN-AML patients offers insight into the biological changes associated with different *RUNX1* expression levels.

## MATERIALS AND METHODS

### Patients and treatment

In the testing group, 157 patients with previously untreated CN-AML (median age, 50 years; range, 16–77 years) were studied. All patients received uniform therapeutic treatment based on study protocols of the Dutch-Belgian Hemato-Oncology Cooperative Group (HOVON) between 1990 and 2008 (The details of therapeutic protocol are available at http://www.hovon.nl) [53] (Supplementary Figure 4). One hundred thirty patients (83%) were aged < 60 years (younger patients) and 27 patients (17%) were ≥ 60 years (older patients). The diagnosis of normal karyotype AML was based on conventional cytogenetic examination of at least 20 metaphases from BM. Patients were assessed for *NPM1*, *CEBPA, N-RAS*, *K-RAS*, *IDH1*, and *IDH2* mutations, *FLT3*-ITD, and tyrosine kinase domain mutations (*FLT3*-TKD [D835]). Clinical, cytogenetic and molecular information, as well as the gene expression profiles for all primary AML cases, can be publicly downloaded from the Gene Expression Omnibus (*www.ncbi.nlm.nih.gov/geo,* accession number *GSE6891*) [53]. This research was approved by the institutional review boards at Weill Cornell Medical College and Erasmus University Medical Center, and written informed consent was obtained from all patients in accordance with the Declaration of Helsinki. Another independent validation group of 162 CN-AML patients received uniform therapeutic treatment provided as part the multicenter AMLCG-1999 trial, which was used to validate our findings. These patients received intensive double induction and consolidation chemotherapy. Gene expression data are publicly available (*http://www.ncbi.nlm.nih.gov/geo/*, accession number *GSE12417*) [54]. The AMLCG-1999 clinical trials were approved by the local institutional review boards, and informed consent from all patients was obtained in accordance with the Declaration of Helsinki [54].

### Microarray analyses

Gene expression and methylation data have been previously published (accession number *GSE1159 [27], GSE9476 [28], GSE6891 [53]* and *GSE12417 [54]* for expression, The Cancer Genome Atlas (TCGA) [50] for methylation). Briefly, gene expression data were obtained using Affymetrix Human Genome 133 plus 2.0 and U133A Gene Chips. All the designs and quality control for microarray experiment were according to the standard Affymetrix protocols. Expression data for microRNA were from TCGA obtained using whole-genome high-throughput sequencing, which provided 79 CN-AML patients [50]. In addition, genome-wide methylation levels in these patients were determined using Illumina 450K chips [50]. Patients with *RUNX1* expression values above the median of all patients were classified as having *RUNX1*^high^, and the others were considered to have *RUNX1*^low^. Levels of *ERG*, *BAALC*, *LEF1*, *MN1*, *WT1, DNMT3B*, *TCF4*, *MIR155HG, ITPR2* and *MAPKBP1* expression were also determined from the microarray data.

### Statistical analyses

The time from the date of diagnosis to death due to any cause defined OS, and the time from the date of diagnosis to removal from the study due to the absence of complete remission, relapse or death defined EFS. Because we found that *RUNX1* expression is normally distributed, a distribution of the cohort based on the highest 50% (*RUNX1*^high^) and the lowest 50% *RUNX1* expression (*RUNX1*^low^) was used for further analysis (Supplementary Figure 5). The Kaplan-Meier method was then used to estimate the association between *RUNX1* expression and the OS and EFS, which were further validated using the log-rank test. To investigate the associations between *RUNX1* expression levels and clinical, molecular characteristics, the Fisher exact and Wilcoxon rank-sum tests were used for hypothesis testing with categorical and continuous variables, respectively. In addition, multivariable Cox proportional hazard models were used to study how *RUNX1* expression levels were associated with OS and EFS in the presence of other known risk factors. With the two groups divided based on *RUNX1* expression levels, Student's *t*-test and multiple hypothesis correction (False Discovery Rate, FDR) was used to identify differences in gene/microRNA expression and DNA methylation profiles. The statistical cutoff values were an absolute fold-change (FC) ≥ 1.5 and an adjusted *P*-value ≤ 0.05. All analyses were performed using the R 3.1.1 software packages.

## SUPPLEMENTARY MATERIALS TABLES AND FIGURES


