# Supplementary material for: High expression of RUNX1 is associated with poorer outcomes in cytogenetically normal acute myeloid leukemia
Source: Oncotarget. 2016 Feb 19;7(13):15828–39. doi: 10.18632/oncotarget.7489 (PMC4941280; doi:10.18632/oncotarget.7489)
Supplement: Supplementary file 1 [file oncotarget-07-15828-s001.pdf]

High expression of *RUNX1* is associated with poorer outcomes in cytogenetically normal acute myeloid leukemia

Supplementary Materials

Supplementary Table S1: Patients' characteristics in the validating group of CN-AML according to the *RUNX1* expression

Variable	*RUNX1*^{high}, *n* = 81	*RUNX1*^{low}, *n* = 81	*P*
Median age. y (range)	59 (7–81)	56 (20–83)	0.82
Median OS. d (range)	223 (3–1176)	416 (1–1176)	0.03
FAB subtype, no			
M0	2	3	1
M1	30	15	0.014
M2	18	27	0.16
M3	0	0	1
M4	22	20	0.86
M5	8	11	0.63
M6	1	5	0.21
High *ERG*, no	61	20	< 0.001
High *BAALC*, no	48	33	0.028
High *LEF1*, no	28	53	< 0.001
High *MN1*, no	44	37	0.35
High *WT1*, no	54	27	< 0.001
High *DNMT3B*, no	56	25	< 0.001
High T*CF4*, no	60	21	< 0.001
High *ITPR2*, no	63	18	< 0.001
High *MAPKBP1*, no	58	23	< 0.001

High *ERG, BAALC, LEF1, MN1,WT1, DNMT3B TCF4, ITPR2* and *MAPKBP1* expression were defined as an expression level above the median of all samples, respectively.

Supplementary Table S2: *RUNX1*-associated gene-expression profile using microarray in the testing group

probes	Symbols	*p*-value	Fold change	Meanexpression of RUNX1high	Mean expression of RUNX1low
209360_s_at	RUNX1	3.68E-34	1.311753	9.681265	8.369512
228280_at	ZC3HAV1L	1.37E-16	1.255878	8.327701	7.071823
203151_at	MAP1A	1.77E-16	1.239758	8.022762	6.783004
217892_s_at	LIMA1	1.89E-15	0.676639	7.784194	7.107555
209537_at	EXTL2	5.47E-15	0.856769	8.097405	7.240636
225295_at	SLC39A10	4.59E-15	0.814251	8.747474	7.933222
228550_at	RTN4R	5.27E-15	0.910439	6.692529	5.782091
223351_at	C17orf80	1.15E-14	0.690112	7.413718	6.723606
224959_at	SLC26A2	5.64E-14	0.637026	8.395491	7.758465
209434_s_at	PPAT	6.33E-14	0.644608	6.955966	6.311358
229530_at	GUCY1A3	1.05E-13	1.580208	8.302874	6.722666
227335_at	DIDO1	1.74E-13	0.736967	7.494155	6.757188
213391_at	DPY19L4	2.21E-13	0.762041	6.802641	6.0406
226721_at	DPY19L4	3.21E-13	0.703803	7.753397	7.049594
227426_at	SOS1	3.07E-13	0.635517	7.875537	7.24002
239237_at	LOC100506776	3.23E-13	1.266409	8.29596	7.029551
218361_at	GOLPH3L	3.89E-13	0.83911	8.197894	7.358783
205097_at	SLC26A2	4.34E-13	0.763387	7.608132	6.844745
223253_at	EPDR1	6.21E-13	1.147946	8.758419	7.610474
206081_at	SLC24A1	7.08E-13	0.696113	6.15335	5.457237
212653_s_at	EHBP1	8.41E-13	0.68067	7.553831	6.873161
209409_at	GRB10	9.52E-13	1.288065	8.411074	7.123009
222589_at	NLK	1.06E-12	0.831837	8.267342	7.435505
228095_at	PHF14	1.16E-12	0.73065	8.454919	7.72427
221942_s_at	GUCY1A3	1.65E-12	1.621215	9.099951	7.478736
212731_at	ANKRD46	1.83E-12	0.679341	6.939268	6.259927
218783_at	INTS7	2.05E-12	0.675563	6.265776	5.590213
213395_at	MLC1	2.37E-12	1.190422	8.489091	7.298668
1555881_s_at	LZTS2	3.02E-12	0.644231	7.859994	7.215763
227522_at	CMBL	3.01E-12	1.235737	8.013945	6.778208
228696_at	SLC45A3	3.35E-12	0.889049	7.988856	7.099808
204866_at	PHF16	4.10E-12	0.690149	6.661758	5.971609
227461_at	STON2	4.03E-12	1.391123	7.703123	6.312001
40472_at	LPCAT4	4.09E-12	0.652861	7.178933	6.526072
224746_at	KIAA1522	4.30E-12	0.801332	6.404184	5.602852
221652_s_at	ASUN	5.41E-12	0.691622	9.063173	8.37155
229983_at	TIGD2	5.90E-12	0.613728	6.945454	6.331725
227334_at	USP54	6.82E-12	0.650549	6.790105	6.139556
238499_at	SLC45A3	7.59E-12	0.82616	7.007895	6.181735
223403_s_at	POLR1B	8.95E-12	0.647463	7.789318	7.141855
242414_at	QPRT	9.43E-12	1.045506	7.57447	6.528965

212828_at	SYNJ2	9.94E-12	0.885825	7.022216	6.13639
218889_at	NOC3L	1.38E-11	0.605745	7.732919	7.127174
225543_at	GTF3C4	1.64E-11	0.615082	6.50118	5.886098
227235_at	GUCY1A3	1.90E-11	1.680967	8.01135	6.330384
1553218_a_at	ZNF512	1.94E-11	0.599511	8.430813	7.831302
202661_at	ITPR2	2.03E-11	0.837715	6.131988	5.294272
219497_s_at	BCL11A	2.04E-11	1.127098	9.235414	8.108317
217263_x_at	RUNX1	2.08E-11	0.644412	6.210369	5.565958
227026_at	MPHOSPH8	2.08E-11	0.598302	8.002568	7.404266
213035_at	ANKRD28	2.12E-11	0.960183	8.405162	7.444979
203372_s_at	SOCS2	2.26E-11	1.652714	7.630611	5.977898
206067_s_at	WT1	3.30E-11	1.354219	7.727775	6.373556
202630_at	APPBP2	3.43E-11	0.647776	7.048086	6.40031
226043_at	GPSM1	3.47E-11	1.137794	8.489718	7.351924
212170_at	RBM12	3.72E-11	0.660824	6.597278	5.936454
201664_at	SMC4	3.77E-11	0.827573	9.984242	9.156669
219372_at	IFT81	4.54E-11	0.64351	5.838252	5.194743
211181_x_at	RUNX1	5.07E-11	0.718017	6.635991	5.917975
201069_at	MMP2	5.54E-11	1.799152	7.976892	6.17774
203373_at	SOCS2	5.84E-11	1.675798	8.564958	6.889161
214670_at	ZKSCAN1	6.62E-11	0.649262	8.196284	7.547022
229090_at	ZEB1-AS1	7.40E-11	0.668814	5.987087	5.318273
225384_at	DOCK7	7.88E-11	0.650811	7.464311	6.8135
214806_at	BICD1	8.21E-11	0.629724	5.905583	5.275859
227536_at	ZC3H13	8.63E-11	0.693706	7.91103	7.217324
229614_at	ZNF320	9.02E-11	0.809031	6.510343	5.701311
210347_s_at	BCL11A	9.31E-11	0.77071	8.379278	7.608568
213237_at	C16orf88	1.06E-10	0.619919	6.23158	5.611661
204044_at	QPRT	1.11E-10	1.091554	6.691436	5.599883
211793_s_at	ABI2	1.19E-10	0.740015	7.295337	6.555322
225112_at	ABI2	1.37E-10	0.800442	7.825411	7.024969
225611_at	MAST4	1.43E-10	0.835822	7.550219	6.714397
225613_at	MAST4	1.50E-10	0.826681	6.768808	5.942127
238122_at	RBM12B	1.49E-10	0.585472	6.720511	6.135038
222742_s_at	RABL5	1.52E-10	0.739888	6.628553	5.888665
211180_x_at	RUNX1	1.68E-10	0.617015	6.932926	6.315911
209014_at	MAGED1	1.89E-10	0.791657	9.06096	8.269303
226157_at	TFDP2	1.92E-10	0.962232	9.333389	8.371158
219615_s_at	KCNK5	1.96E-10	1.048902	7.155858	6.106956
226098_at	IFT80	2.12E-10	0.650002	8.027738	7.377736
224827_at	UBTD2	2.34E-10	0.656654	7.770819	7.114165
225237_s_at	MSI2	2.42E-10	1.255849	9.39492	8.139072
203735_x_at	PPFIBP1	2.45E-10	0.718166	7.225566	6.5074
202561_at	TNKS	2.93E-10	0.617221	7.363787	6.746567
201243_s_at	ATP1B1	3.11E-10	1.070357	8.81351	7.743154

219498_s_at	BCL11A	3.13E-10	0.950582	8.313535	7.362953
228920_at	ZNF260	3.21E-10	0.629619	6.630745	6.001126
213005_s_at	KANK1	3.24E-10	0.89935	7.755941	6.856591
220992_s_at	TRMT1L	3.39E-10	0.650217	6.992926	6.342709
227604_at	TMEM185B	3.71E-10	0.586977	6.05621	5.469233
238050_at	ANTXR2	4.55E-10	0.649476	6.260733	5.611257
212274_at	LPIN1	4.75E-10	0.598481	7.298144	6.699663
228397_at	TUG1	4.99E-10	0.661525	6.986701	6.325176
230178_s_at	ELP2	5.01E-10	0.644267	6.856106	6.211839
1559078_at	BCL11A	5.89E-10	0.916715	7.161695	6.24498
204341_at	TRIM16	6.00E-10	0.813715	7.243345	6.429629
55872_at	ZNF512B	6.74E-10	0.647865	7.887559	7.239694
222891_s_at	BCL11A	6.83E-10	1.085605	9.086235	8.000631
225240_s_at	MSI2	6.94E-10	1.177446	9.750959	8.573513
229971_at	GPR114	7.83E-10	0.908985	7.60908	6.700096
222457_s_at	LIMA1	7.96E-10	0.623839	5.84958	5.225742
1558700_s_at	ZNF260	8.10E-10	0.651056	6.188681	5.537626
210694_s_at	MID1	9.40E-10	0.64261	6.35615	5.71354
230298_at	MBLAC2	1.05E-09	0.667036	7.280972	6.613935
212405_s_at	METTL13	1.07E-09	0.617068	8.059085	7.442016
241365_at	SATB1	1.09E-09	0.682854	7.377938	6.695084
235852_at	STON2	1.12E-09	1.103987	6.397485	5.293499
204484_at	PIK3C2B	1.15E-09	0.950514	7.777493	6.826979
235151_at	LOC283357	1.17E-09	0.682919	6.629435	5.946517
215146_s_at	TTC28	1.19E-09	0.743805	6.541984	5.798179
225098_at	ABI2	1.19E-09	0.915933	7.649941	6.734008
228171_s_at	PLEKHG4	1.25E-09	0.692028	6.750913	6.058885
226085_at	CBX5	1.34E-09	0.731947	8.804413	8.072465
201663_s_at	SMC4	1.37E-09	0.842318	9.672025	8.829706
212276_at	LPIN1	1.38E-09	0.610847	8.448127	7.83728
218694_at	ARMCX1	1.40E-09	0.837175	7.060708	6.223533
207011_s_at	PTK7	1.48E-09	0.884413	6.281164	5.396751
228442_at	NFATC2	1.48E-09	0.837461	7.321464	6.484004
236738_at	C3orf80	1.56E-09	1.628542	7.682784	6.054241
205624_at	CPA3	1.73E-09	1.879526	9.735435	7.855909
218966_at	MYO5C	1.79E-09	0.782115	7.590253	6.808138
209386_at	TM4SF1	1.87E-09	1.280377	7.292272	6.011895
226299_at	PKN3	2.28E-09	0.728137	6.168645	5.440508
1557953_at	ZKSCAN1	2.37E-09	0.628656	7.753049	7.124393
227569_at	LNX2	2.38E-09	0.616788	6.505421	5.888633
211182_x_at	RUNX1	2.63E-09	0.856856	6.555879	5.699024
212186_at	ACACA	2.69E-09	0.881249	7.453035	6.571787
222819_at	CTPS2	2.70E-09	0.588708	7.239247	6.650538
212070_at	GPR56	2.76E-09	1.595233	8.231286	6.636054
219330_at	VANGL1	2.80E-09	0.780673	7.250418	6.469744

207160_at	IL12A	2.89E-09	0.590672	5.586478	4.995807
242628_at	KLRB1	2.97E-09	0.586534	5.711758	5.125224
224722_at	MIB1	2.99E-09	0.649566	6.905868	6.256302
228654_at	SPIN4	3.01E-09	0.900944	8.821685	7.920741
213610_s_at	KLHL23	3.04E-09	0.873247	6.61238	5.739133
218318_s_at	NLK	3.76E-09	0.604541	6.988508	6.383967
213058_at	TTC28	3.81E-09	0.611031	6.502538	5.891507
213891_s_at	TCF4	4.40E-09	1.010695	8.391064	7.380369
226327_at	ZNF507	4.79E-09	0.587414	6.675385	6.087972
206188_at	ZNF623	5.09E-09	0.644524	6.351424	5.706901
218437_s_at	LZTFL1	5.90E-09	0.632234	7.652091	7.019857
206478_at	KIAA0125	6.14E-09	1.317048	9.120244	7.803197
203139_at	DAPK1	6.23E-09	0.80582	9.240368	8.434548
230266_at	RAB7B	6.26E-09	0.997714	7.265511	6.267797
202662_s_at	ITPR2	6.61E-09	0.597469	7.112503	6.515034
206954_at	WT1-AS	6.87E-09	0.706167	5.622387	4.91622
235604_x_at	ZNF493	8.02E-09	0.644125	6.610064	5.965939
224901_at	SCD5	8.78E-09	0.838157	6.754203	5.916045
239580_at	GUCY1A3	8.78E-09	1.160071	6.30121	5.141139
210365_at	RUNX1	8.99E-09	0.894292	7.856704	6.962412
201242_s_at	ATP1B1	9.36E-09	1.098107	9.116725	8.018619
202660_at	ITPR2	1.08E-08	0.64533	7.94127	7.29594
212692_s_at	LRBA	1.10E-08	0.591174	8.485203	7.894029
218689_at	FANCF	1.12E-08	0.736392	6.739004	6.002612
218792_s_at	BSPRY	1.16E-08	0.724867	6.751989	6.027122
230434_at	PHOSPHO2	1.28E-08	0.637819	6.625965	5.988146
212387_at	TCF4	1.30E-08	0.902643	7.85296	6.950317
217975_at	WBP5	1.46E-08	1.145313	7.822912	6.677599
207426_s_at	TNFSF4	1.50E-08	0.738166	7.500857	6.762691
215471_s_at	MAP7	1.50E-08	0.950887	7.04723	6.096343
218984_at	PUS7	1.53E-08	0.637653	7.457865	6.820213
232007_at	AGPAT5	1.60E-08	0.758791	7.173154	6.414363
223382_s_at	ZNRF1	1.60E-08	0.660617	8.871701	8.211084
209627_s_at	OSBPL3	1.70E-08	0.655407	7.418257	6.76285
226771_at	ATP8B2	1.74E-08	0.957116	7.605852	6.648736
218710_at	TTC27	1.83E-08	0.734191	8.010629	7.276438
226889_at	WDR35	1.86E-08	0.5946	7.191059	6.59646
244043_at	TFDP2	1.95E-08	0.795148	7.053123	6.257974
218641_at	C11orf95	2.05E-08	0.704776	7.491804	6.787028
204040_at	RNF144A	2.12E-08	0.750279	7.75904	7.00876
223268_at	C11orf54	2.15E-08	0.59396	8.009487	7.415527
224953_at	YIPF5	2.20E-08	0.713402	7.326959	6.613557
223599_at	TRIM6	2.38E-08	0.941034	6.438198	5.497164
1558956_s_at	IFT80	2.42E-08	0.702345	8.123645	7.421301
202252_at	RAB13	2.52E-08	0.976817	8.81867	7.841853

225081_s_at	CDCA7L	2.84E-08	0.835734	9.288104	8.45237
223075_s_at	AIF1L	3.03E-08	1.132528	6.850402	5.717874
202889_x_at	MAP7	3.08E-08	0.949628	6.645113	5.695486
212599_at	AUTS2	3.13E-08	0.851538	7.547799	6.696262
213258_at	TFPI	3.54E-08	1.158978	7.84644	6.687462
213394_at	MAPKBP1	4.04E-08	0.889245	6.912516	6.02327
233461_x_at	ZNF226	4.17E-08	0.870445	6.293058	5.422613
238043_at	ARID1B	4.25E-08	0.65737	7.754008	7.096638
210958_s_at	MAST4	4.41E-08	0.627104	6.272856	5.645752
222746_s_at	BSPRY	4.49E-08	0.85725	7.400879	6.543628
206582_s_at	GPR56	5.01E-08	1.139251	6.471457	5.332205
222451_s_at	ZDHHC9	5.01E-08	0.917335	6.704761	5.787426
225671_at	SPNS2	5.70E-08	0.86535	7.792498	6.927147
205739_x_at	ZNF107	6.30E-08	0.593262	7.207121	6.613859
225550_at	CNST	6.34E-08	0.655581	7.237765	6.582183
218596_at	TBC1D13	6.46E-08	0.884317	6.561023	5.676706
229492_at	VANGL1	6.53E-08	0.929416	6.104295	5.174878
222594_s_at	SPATS2	6.73E-08	0.590331	6.06967	5.47934
212775_at	OBSL1	6.77E-08	1.252799	7.251761	5.998962
238418_at	SLC35B4	6.78E-08	0.634012	6.828204	6.194192
203685_at	BCL2	6.82E-08	0.71907	8.091367	7.372297
207877_s_at	NVL	7.28E-08	0.625757	5.703095	5.077338
238732_at	COL24A1	7.29E-08	1.068372	7.123518	6.055145
205527_s_at	GEMIN4	7.40E-08	0.588464	7.130536	6.542073
238021_s_at	CRNDE	8.38E-08	1.534486	8.135111	6.600625
202890_at	MAP7	8.93E-08	1.233649	7.855193	6.621544
223961_s_at	CISH	9.10E-08	0.777771	6.709052	5.931281
225959_s_at	ZNRF1	9.62E-08	0.603358	8.171118	7.56776
212110_at	SLC39A14	9.99E-08	0.589674	6.810692	6.221018
232489_at	TRMT13	1.05E-07	0.637991	6.873011	6.235019
223513_at	CENPJ	1.10E-07	0.665195	7.539537	6.874342
219553_at	NME7	1.10E-07	0.64602	7.258876	6.612857
205497_at	ZNF175	1.13E-07	0.600278	6.694774	6.094496
212386_at	TCF4	1.13E-07	0.978415	9.026603	8.048188
229572_at	ATP6V0A2	1.14E-07	0.705652	7.539911	6.83426
235900_at	SPNS3	1.15E-07	1.005015	7.786844	6.78183
225551_at	CNST	1.18E-07	0.621014	7.174039	6.553025
1553982_a_at	RAB7B	1.19E-07	0.965948	6.956019	5.990071
40016_g_at	MAST4	1.19E-07	0.814327	6.294002	5.479675
213541_s_at	ERG	1.25E-07	1.009922	7.931104	6.921182
205190_at	PLS1	1.27E-07	0.942638	6.482297	5.53966
217841_s_at	PPME1	1.30E-07	0.622004	7.249404	6.627401
225485_at	CEP41	1.39E-07	0.630987	7.182834	6.551847
228174_at	SCAI	1.45E-07	0.616443	7.168679	6.552236
224847_at	CDK6	1.47E-07	0.797717	10.88061	10.08289

220918_at	RUNX1-IT1	1.52E-07	0.702144	7.06045	6.358306
227792_at	ITPRIPL2	1.65E-07	0.917805	7.808187	6.890382
231319_x_at	KIF9	1.79E-07	0.639944	7.161309	6.521366
218762_at	ZNF574	1.83E-07	0.734697	6.894685	6.159987
229997_at	VANGL1	1.84E-07	0.970547	7.079994	6.109447
223404_s_at	TRMT1L	1.84E-07	0.642233	7.180933	6.5387
216705_s_at	ADA	1.90E-07	1.061197	8.809603	7.748407
227766_at	LIG4	1.91E-07	0.607359	6.924523	6.317165
203865_s_at	ADARB1	2.01E-07	0.637591	6.632222	5.994631
219602_s_at	PIEZO2	2.11E-07	0.840759	6.211832	5.371072
222240_s_at	ISYNA1	2.29E-07	0.666745	7.564657	6.897912
227798_at	SMAD1	2.32E-07	0.783278	7.570494	6.787217
202813_at	TARBP1	2.35E-07	0.669412	8.846837	8.177426
223758_s_at	GTF2H2	2.40E-07	0.595513	7.488536	6.893022
201440_at	DDX23	2.45E-07	0.690613	7.211156	6.520544
203608_at	ALDH5A1	2.82E-07	1.009942	8.146988	7.137046
226991_at	NFATC2	2.87E-07	0.862183	6.629454	5.767271
236837_x_at	MIPEPP3	3.04E-07	0.622202	6.008464	5.386262
220668_s_at	DNMT3B	3.05E-07	0.689914	7.963872	7.273958
204639_at	ADA	3.07E-07	0.938825	9.363046	8.424221
219353_at	NHLRC2	3.10E-07	0.635158	6.355975	5.720818
229838_at	NUCB2	3.33E-07	0.614985	8.61494	7.999955
230381_at	C1orf186	3.32E-07	0.868651	8.3686	7.499949
210999_s_at	GRB10	3.38E-07	0.899695	7.496669	6.596974
243963_at	SDCCAG8	3.40E-07	0.59286	6.911274	6.318414
213489_at	MAPRE2	3.45E-07	0.773439	5.700956	4.927516
210993_s_at	SMAD1	3.75E-07	0.690496	7.203781	6.513286
221031_s_at	APOLD1	3.81E-07	0.590035	5.63371	5.043675
219036_at	CEP70	4.08E-07	0.814867	7.017674	6.202807
201596_x_at	KRT18	4.72E-07	1.239223	7.185003	5.945779
222146_s_at	TCF4	5.46E-07	0.799326	7.685545	6.886219
218397_at	FANCL	5.58E-07	0.615572	8.081972	7.4664
242260_at	MATR3	6.02E-07	0.743644	7.874953	7.131309
231897_at	PTGR1	6.08E-07	0.614372	6.120257	5.505885
227329_at	ZBTB46	6.38E-07	0.603366	6.486488	5.883122
206148_at	IL3RA	6.45E-07	0.981564	6.89698	5.915416
212651_at	RHOBTB1	6.52E-07	0.750917	6.476541	5.725624
223475_at	CRISPLD1	7.22E-07	0.692003	6.437091	5.745089
239232_at	MSI2	7.29E-07	0.633894	7.11738	6.483486
201564_s_at	FSCN1	7.42E-07	0.92634	7.132529	6.206189
202862_at	FAH	7.89E-07	0.590804	7.778463	7.18766
225046_at	LOC389831	7.96E-07	0.852286	6.795989	5.943703
221223_x_at	CISH	8.08E-07	0.587588	7.614699	7.027111
227908_at	TBC1D24	8.22E-07	0.614715	6.510785	5.896071
222811_at	FTSJD1	9.00E-07	0.595697	7.859019	7.263322

202932_at	YES1	9.03E-07	0.796689	6.670322	5.873632
241376_at	LOC100130097	9.08E-07	0.630471	6.975627	6.345155
226025_at	ANKRD28	9.28E-07	0.698185	10.2299	9.531716
203753_at	TCF4	9.34E-07	0.785689	8.134384	7.348695
223627_at	MEX3B	9.46E-07	0.820627	6.901553	6.080926
204429_s_at	SLC2A5	1.00E-06	0.617154	8.045323	7.428169
222603_at	ERMP1	1.01E-06	0.640615	8.67168	8.031065
1552364_s_at	MSI2	1.11E-06	0.728851	6.681239	5.952388
241926_s_at	ERG	1.12E-06	0.781151	6.99762	6.21647
210473_s_at	GPR125	1.21E-06	0.917457	6.975635	6.058179
224848_at	CDK6	1.22E-06	0.772126	10.33227	9.560144
218096_at	AGPAT5	1.23E-06	0.620626	9.300837	8.680211
205227_at	IL1RAP	1.34E-06	0.892905	8.364958	7.472054
238022_at	CRNDE	1.35E-06	1.115255	6.753048	5.637794
243579_at	MSI2	1.42E-06	0.89907	7.089033	6.189963
206674_at	FLT3	1.54E-06	0.770561	10.22171	9.451144
224851_at	CDK6	1.72E-06	0.741963	11.3552	10.61323
225233_at	MSI2	1.75E-06	0.948288	6.690636	5.742348
215143_at	DPY19L2P2	1.88E-06	0.59268	6.548085	5.955405
203069_at	SV2A	1.88E-06	0.825813	6.773621	5.947808
228885_at	MAMDC2	1.96E-06	1.358977	7.652703	6.293726
1554067_at	C12orf66	1.99E-06	0.663762	7.617666	6.953904
232232_s_at	SLC22A16	2.02E-06	0.590903	7.902477	7.311574
228245_s_at	OVOS2	2.05E-06	0.596314	7.395387	6.799074
201427_s_at	SEPP1	2.29E-06	1.72918	9.309376	7.580196
212382_at	TCF4	2.41E-06	0.823085	7.627322	6.804237
239162_at	DAPK1-IT1	2.56E-06	0.606295	6.651312	6.045016
219871_at	FLJ13197	2.72E-06	0.904319	7.407447	6.503129
215116_s_at	DNM1	2.74E-06	0.795653	6.368983	5.57333
213670_x_at	NSUN5P1	2.84E-06	0.586963	8.095171	7.508208
226550_at	SLC9A1	2.86E-06	0.631609	7.97686	7.345251
215034_s_at	TM4SF1	2.86E-06	0.895439	5.896306	5.000867
205051_s_at	KIT	3.01E-06	1.154241	9.10535	7.95111
203688_at	PKD2	3.04E-06	0.612595	8.010914	7.398319
205609_at	ANGPT1	3.30E-06	1.083521	8.787223	7.703702
206589_at	GFI1	3.31E-06	0.862855	7.952539	7.089684
225238_at	MSI2	3.41E-06	0.70299	5.909288	5.206297
241682_at	KLHL23	3.49E-06	0.696462	6.049998	5.353536
212385_at	TCF4	3.54E-06	0.752461	7.274326	6.521865
222659_at	IPO11	3.67E-06	0.623239	6.816933	6.193695
209930_s_at	NFE2	3.72E-06	0.667803	9.656321	8.988518
214375_at	PPFIBP1	3.82E-06	0.602649	6.184658	5.582009
221286_s_at	MZB1	3.93E-06	0.829156	7.046695	6.217538
203588_s_at	TFDP2	4.04E-06	0.682948	7.884271	7.201323
216953_s_at	WT1	4.22E-06	0.799979	5.801284	5.001305

200986_at	SERPING1	4.24E-06	0.814006	7.547773	6.733766
202431_s_at	MYC	4.95E-06	0.790635	9.747933	8.957297
205349_at	GNA15	5.05E-06	0.635243	9.740821	9.105578
202566_s_at	SVIL	5.35E-06	0.598317	7.085578	6.487261
219412_at	RAB38	5.50E-06	0.789684	5.750217	4.960533
209710_at	GATA2	5.58E-06	0.886481	8.678826	7.792346
226545_at	CD109	5.98E-06	0.90883	6.708223	5.799393
204798_at	MYB	6.04E-06	0.653744	11.25563	10.60188
223319_at	GPHN	6.25E-06	0.622636	5.975942	5.353306
227492_at	OCLN	6.28E-06	0.733578	7.99306	7.259482
1554489_a_at	CEP70	6.33E-06	0.763973	6.909789	6.145816
229638_at	IRX3	6.38E-06	1.444278	7.277347	5.833068
205229_s_at	COCH	6.71E-06	0.850076	6.744175	5.894099
202933_s_at	YES1	6.74E-06	0.742702	7.067304	6.324602
215248_at	GRB10	6.79E-06	0.721284	6.80954	6.088257
228988_at	ZNF711	8.86E-06	1.132186	6.952875	5.820689
220744_s_at	IFT122	9.02E-06	0.710613	6.754032	6.043419
224150_s_at	CEP70	9.12E-06	0.713851	6.988393	6.274542
225285_at	BCAT1	9.28E-06	0.998204	8.319482	7.321277
212776_s_at	OBSL1	9.42E-06	0.980993	6.100075	5.119083
209387_s_at	TM4SF1	9.62E-06	0.974107	6.257496	5.283389
204083_s_at	TPM2	9.81E-06	0.657273	6.169001	5.511728
202718_at	IGFBP2	1.04E-05	1.340763	7.452017	6.111254
205375_at	MDFI	1.07E-05	0.674172	6.064444	5.390272
220643_s_at	FAIM	1.15E-05	0.593305	6.944301	6.350996
226517_at	BCAT1	1.15E-05	0.888401	7.587341	6.69894
222632_s_at	LZTFL1	1.16E-05	0.658997	6.138986	5.479989
213460_x_at	NSUN5P2	1.17E-05	0.614559	7.977143	7.362584
213110_s_at	COL4A5	1.26E-05	1.267014	7.614152	6.347138
225532_at	CABLES1	1.39E-05	0.618432	6.499303	5.880871
1568619_s_at	ITPRIPL2	1.41E-05	0.679959	7.484201	6.804242
1555842_at	CYTH2	1.42E-05	0.614816	6.101713	5.486897
220377_at	KIAA0125	1.43E-05	1.192792	7.548698	6.355906
229507_at	FAM212A	1.45E-05	0.600668	6.133917	5.533249
203787_at	SSBP2	1.45E-05	0.654673	8.670148	8.015476
1557137_at	TMEM17	1.48E-05	0.588843	5.670979	5.082136
227574_at	OBSL1	1.48E-05	0.752814	6.122229	5.369414
221606_s_at	HMGN5	1.5E-05	0.643363	6.355236	5.711873
210299_s_at	FHL1	1.54E-05	0.834806	7.188513	6.353707
206341_at	IL2RA	1.57E-05	0.683231	5.875505	5.192274
243010_at	MSI2	1.63E-05	0.722749	6.939891	6.217143
214452_at	BCAT1	1.67E-05	0.59985	6.238708	5.638858
225802_at	TOP1MT	1.69E-05	0.678636	8.048546	7.36991
230541_at	LOC149134	1.75E-05	0.747878	6.250525	5.502647
215016_x_at	DST	1.81E-05	0.718186	6.463993	5.745807

204624_at	ATP7B	1.85E-05	0.629626	6.26686	5.637233
205608_s_at	ANGPT1	1.89E-05	0.838176	8.757174	7.918998
218795_at	ACP6	2.11E-05	0.589277	6.952726	6.363449
209676_at	TFPI	2.19E-05	0.676017	6.167455	5.491438
225962_at	ZNRF1	2.23E-05	0.755265	6.527077	5.771812
1552908_at	GCSAML	2.24E-05	0.88248	6.848481	5.966001
219922_s_at	LTBP3	2.32E-05	0.658066	7.204799	6.546734
220952_s_at	PLEKHA5	2.35E-05	0.704598	7.683536	6.978938
217974_at	TM7SF3	2.39E-05	0.598464	8.023228	7.424763
201540_at	FHL1	2.44E-05	1.007672	8.572693	7.565021
206233_at	B4GALT6	2.48E-05	0.841437	7.661901	6.820464
39248_at	AQP3	2.49E-05	0.833954	7.115426	6.281471
214039_s_at	LAPTM4B	2.62E-05	1.238673	9.122628	7.883955
224516_s_at	CXXC5	2.63E-05	0.625382	9.779658	9.154276
219654_at	PTPLA	2.66E-05	0.698135	7.369579	6.671445
212764_at	ZEB1	2.73E-05	0.692822	7.024449	6.331627
212254_s_at	DST	2.82E-05	0.712088	6.348676	5.636588
223374_s_at	B3GALNT1	2.83E-05	0.630228	6.898272	6.268044
1555600_s_at	APOL4	3.2E-05	0.723707	6.115384	5.391676
215111_s_at	TSC22D1	3.45E-05	0.79458	9.882944	9.088364
215440_s_at	BEX4	3.47E-05	0.728113	8.937329	8.209216
201417_at	SOX4	3.5E-05	0.70002	10.90419	10.20417
1554679_a_at	LAPTM4B	3.61E-05	1.002147	7.772053	6.769906
204430_s_at	SLC2A5	3.61E-05	0.846528	8.242106	7.395578
224759_s_at	C12orf23	3.66E-05	0.678772	7.600641	6.921869
224049_at	KCNK17	3.77E-05	0.681965	7.645702	6.963737
231899_at	ZC3H12C	4.12E-05	0.670962	5.967772	5.29681
210933_s_at	FSCN1	4.16E-05	0.798445	8.32634	7.527896
209702_at	FTO	4.2E-05	0.701409	8.891552	8.190143
205801_s_at	RASGRP3	4.36E-05	0.9947	7.653915	6.659215
202351_at	ITGAV	4.39E-05	0.626726	7.14063	6.513904
236562_at	ZNF439	4.47E-05	0.662357	6.543027	5.88067
210805_x_at	RUNX1	4.85E-05	0.616925	5.694689	5.077765
209191_at	TUBB6	5.1E-05	0.932368	8.631533	7.699166
208767_s_at	LAPTM4B	5.28E-05	0.898084	8.259822	7.361738
210612_s_at	SYNJ2	5.51E-05	0.642691	5.608628	4.965937
201418_s_at	SOX4	5.79E-05	0.94421	8.740306	7.796096
206331_at	CALCRL	6.1E-05	0.660068	5.794831	5.134763
1554242_a_at	COCH	6.23E-05	0.686721	6.016401	5.32968
211071_s_at	MLLT11	6.29E-05	0.651137	8.21547	7.564333
217904_s_at	BACE1	6.43E-05	0.60166	6.178223	5.576563
225355_at	NEURL1B	6.54E-05	0.913686	6.996925	6.083239
208029_s_at	LAPTM4B	6.65E-05	1.125371	8.014379	6.889008
224048_at	USP44	7.06E-05	0.616305	5.642555	5.02625
235109_at	ZBED3	7.84E-05	0.694566	7.021254	6.326688

235142_at	ZBTB8A	7.9E-05	0.918937	7.938283	7.019345
226670_s_at	PABPC1L	8.37E-05	0.589001	7.578466	6.989465
215767_at	ZNF804A	8.52E-05	0.612531	6.760708	6.148178
223939_at	SUCNR1	8.69E-05	0.976361	9.151075	8.174714
203817_at	GUCY1B3	8.94E-05	0.799497	7.35034	6.550843
223095_at	MARVELD1	9E-05	0.637274	7.804185	7.166911
209035_at	MDK	9.04E-05	0.683746	6.313822	5.630077
1552715_a_at	RXFP1	9.1E-05	0.596115	5.544989	4.948874
232985_s_at	DPPA4	9.66E-05	0.888974	6.519209	5.630235
228372_at	C10orf128	9.85E-05	0.90597	6.556981	5.651011
235171_at	LOC100505501	9.97E-05	0.851648	7.530851	6.679203
207781_s_at	ZNF711	0.000104	0.682264	5.862244	5.179979
201539_s_at	FHL1	0.000106	0.673251	6.097089	5.423838
235333_at	B4GALT6	0.000111	0.842393	8.069688	7.227295
235085_at	SGK223	0.000113	0.632749	6.483424	5.850674
222996_s_at	CXXC5	0.000121	0.59023	8.717809	8.127579
214228_x_at	TNFRSF4	0.000121	0.675358	6.126131	5.450773
210425_x_at	GOLGA8B	0.000122	0.693152	9.367791	8.67464
209099_x_at	JAG1	0.000124	0.896965	8.151203	7.254238
212364_at	MYO1B	0.000127	0.608125	5.463458	4.855333
235287_at	CDK6	0.000129	0.708118	9.149825	8.441707
210239_at	IRX5	0.000133	0.772332	5.776091	5.003759
222719_s_at	PDGFC	0.000136	0.624726	6.53185	5.907124
217963_s_at	NGFRAP1	0.000143	1.108212	8.84187	7.733658
203795_s_at	BCL7A	0.000152	0.629398	6.924486	6.295088
227860_at	CPXM1	0.000158	0.902957	8.693391	7.790434
214505_s_at	FHL1	0.00017	0.941473	6.611862	5.67039
233955_x_at	CXXC5	0.000172	0.595752	9.429218	8.833466
222920_s_at	TESPA1	0.0002	0.624854	7.454886	6.830031
204529_s_at	TOX	0.000212	0.795582	7.457742	6.662159
241133_at	TRBV27	0.000215	1.068105	8.596753	7.528648
206298_at	ARHGAP22	0.000225	0.898223	5.736252	4.838029
214390_s_at	BCAT1	0.000226	0.589869	6.195476	5.605607
210100_s_at	ABCA2	0.000236	0.629759	6.230936	5.601178
216268_s_at	JAG1	0.000262	0.911887	7.867904	6.956016
231982_at	C19orf77	0.000267	0.938756	8.560813	7.622057
210815_s_at	CALCRL	0.000273	0.585023	5.462048	4.877025
213415_at	CLIC2	0.000298	0.766514	6.282669	5.516155
223565_at	MZB1	0.000319	0.701362	6.236702	5.53534
205780_at	BIK	0.000319	0.837719	6.026114	5.188395
210298_x_at	FHL1	0.000335	0.815924	6.317342	5.501418
1555923_a_at	C10orf114	0.000343	0.595947	5.570599	4.974651
226282_at	PTPN14	0.000367	0.878254	5.872693	4.994439
220798_x_at	LPPR3	0.000388	0.648149	7.086067	6.437918
240766_at	EPC1	0.000413	0.959315	7.414098	6.454783

208798_x_at	GOLGA8A	0.000419	0.7433	9.24558	8.50228
236553_at	LOC100507520	0.000419	0.594676	6.555712	5.961035
202806_at	DBN1	0.000457	0.65793	6.493869	5.83594
204082_at	PBX3	0.000466	0.844513	9.100676	8.256163
205479_s_at	PLAU	0.000479	0.589109	6.458919	5.86981
222281_s_at	LOC100505650	0.000531	0.683173	6.625972	5.9428
218086_at	NPDC1	0.000547	0.748833	6.390581	5.641748
1565162_s_at	MGST1	0.000557	0.748353	9.901949	9.153596
228011_at	FAM92A1	0.000596	0.622642	5.765276	5.142635
242172_at	MEIS1	0.000626	0.732239	7.030773	6.298534
204069_at	MEIS1	0.000636	0.943984	8.26407	7.320087
209160_at	AKR1C3	0.000646	0.788867	7.671752	6.882885
225782_at	MSRB3	0.000719	0.678508	9.425228	8.746721
225665_at	ZAK	0.000743	0.637672	7.675723	7.038051
201416_at	SOX4	0.000783	0.798647	8.868024	8.069377
213668_s_at	SOX4	0.000807	0.930245	8.228082	7.297837
244533_at	PTPN14	0.000858	0.619533	5.609589	4.990056
205612_at	MMRN1	0.000944	0.673663	6.692272	6.018609
231736_x_at	MGST1	0.00096	0.668775	10.40005	9.73127
243110_x_at	NPW	0.00097	0.728954	5.388839	4.659885
238206_at	RXFP1	0.000978	0.695497	5.530796	4.835299
204165_at	WASF1	0.001022	0.726069	7.756582	7.030513
210664_s_at	TFPI	0.001095	0.633681	6.857935	6.224254
231767_at	HOXB4	0.001097	0.588521	7.149183	6.560663
211748_x_at	PTGDS	0.001206	0.808763	6.494578	5.685815
224918_x_at	MGST1	0.00122	0.644623	10.50847	9.863845
201830_s_at	NET1	0.001241	0.609782	8.33044	7.720658
204548_at	STAR	0.001258	0.622667	6.218694	5.596028
206574_s_at	PTP4A3	0.001315	0.640281	6.423388	5.783107
1566764_at	MACC1	0.00138	0.61013	6.097666	5.487536
206637_at	P2RY14	0.0014	0.602824	6.296498	5.693674
230670_at	IGSF10	0.001437	0.674872	7.523435	6.848563
205453_at	HOXB2	0.001473	0.956468	8.360682	7.404214
206310_at	SPINK2	0.001568	1.031873	8.8508	7.818927
213260_at	FOXC1	0.001651	0.828091	7.052017	6.223926
238784_at	DPY19L2	0.001745	0.681382	6.91133	6.229948
230158_at	DPY19L2	0.001861	0.628032	6.527889	5.899857
221004_s_at	ITM2C	0.001959	0.627352	8.879225	8.251873
202729_s_at	LTBP1	0.001989	0.679165	7.813368	7.134203
226676_at	ZNF521	0.002062	0.791881	6.224844	5.432964
209487_at	RBPMS	0.002067	0.606994	5.80365	5.196656
213217_at	ADCY2	0.002138	0.592401	6.096154	5.503753
201841_s_at	HSPB1	0.002291	0.594505	7.938106	7.3436
226677_at	ZNF521	0.002394	0.89656	6.301288	5.404728
228904_at	HOXB3	0.002504	1.003047	9.229006	8.225959

227923_at	SHANK3	0.002836	0.769042	6.517461	5.74842
207836_s_at	RBPMS	0.002965	0.592304	5.387292	4.794988
209488_s_at	RBPMS	0.002978	0.701307	5.648666	4.947358
229309_at	ADRB1	0.003111	0.638153	6.161417	5.523264
1555630_a_at	RAB34	0.003131	0.653136	8.582193	7.929057
1559477_s_at	MEIS1	0.003755	0.774195	7.479992	6.705797
1566363_at	DNTT	0.004659	1.047745	7.26976	6.222016
219218_at	BAHCC1	0.00565	0.604503	8.703588	8.099084
235753_at	HOXA7	0.005815	0.657233	7.679461	7.022228
226817_at	DSC2	0.006272	0.719843	7.27338	6.553536
228708_at	RAB27B	0.006277	0.624367	8.182219	7.557852
213479_at	NPTX2	0.007762	0.691524	5.665064	4.97354
206871_at	ELANE	0.008172	1.042487	9.576905	8.534417
1553808_a_at	NKX2-3	0.009171	0.775222	7.138799	6.363578
227297_at	ITGA9	0.009725	0.610096	7.040326	6.43023
209757_s_at	MYCN	0.009905	0.826741	7.696992	6.870251
203305_at	F13A1	0.010319	0.740325	9.006593	8.266267
201324_at	EMP1	0.010613	0.622593	7.427675	6.805082
204438_at	MRC1	0.012584	0.609934	6.483242	5.873308
213844_at	HOXA5	0.013817	0.852044	8.198733	7.346689
207341_at	PRTN3	0.015581	0.855576	8.689356	7.83378
206660_at	IGLL1	0.027079	0.6797	8.819223	8.139523
235521_at	HOXA3	0.027588	0.622885	7.598642	6.975757
221654_s_at	USP3	6.31E-19	−0.89623	8.537594	9.433819
219582_at	OGFRL1	2.21E-12	−0.88141	6.69283	7.574243
210190_at	STX11	3.95E-12	−1.60632	5.290153	6.896476
244556_at	LCP2	6.63E-12	−0.99436	6.007286	7.001651
1553133_at	C9orf72	1.52E-11	−1.23883	5.862473	7.101304
225582_at	ITPRIP	3.63E-11	−0.7273	6.908772	7.636072
213988_s_at	SAT1	6.30E-11	−1.16928	9.062489	10.23177
226026_at	DIRC2	7.08E-11	−0.73919	5.949866	6.689056
209287_s_at	CDC42EP3	8.67E-11	−0.92934	6.234473	7.163813
1552485_at	LACTB	2.51E-10	−0.69828	6.597957	7.296241
230492_s_at	GPCPD1	2.53E-10	−0.63381	7.365984	7.999796
1553134_s_at	C9orf72	3.17E-10	−0.71071	4.956158	5.666867
235593_at	ZEB2	4.10E-10	−0.86928	8.251069	9.120347
241752_at	SLC8A1	4.75E-10	−0.8295	5.350518	6.180017
201926_s_at	CD55	6.33E-10	−0.66382	8.264291	8.928107
238469_at	OGFRL1	6.41E-10	−0.77867	6.70301	7.481678
215223_s_at	SOD2	7.46E-10	−1.3709	7.511311	8.882207
217741_s_at	ZFAND5	8.75E-10	−0.5907	10.52773	11.11843
225685_at	CDC42EP3	9.08E-10	−1.23064	7.265062	8.495702
202205_at	VASP	9.82E-10	−0.82225	8.877406	9.699658
212659_s_at	IL1RN	1.06E-09	−1.02478	5.881044	6.905826
221978_at	HLA-F	1.09E-09	−0.79886	5.904243	6.703104

201502_s_at	NFKBIA	1.47E-09	−0.88873	10.71061	11.59934
230380_at	THAP2	1.75E-09	−0.88076	5.264447	6.145204
207574_s_at	GADD45B	1.82E-09	−1.03431	7.621676	8.655982
229439_s_at	RBM47	2.13E-09	−0.98414	4.61286	5.597004
209286_at	CDC42EP3	3.34E-09	−1.04385	7.031993	8.075845
203927_at	NFKBIE	3.79E-09	−0.74049	6.406853	7.147347
1569599_at	SAMSN1	3.95E-09	−1.31806	5.980768	7.298832
206584_at	LY96	6.42E-09	−0.91533	5.281492	6.19682
210592_s_at	SAT1	6.43E-09	−0.74963	10.33968	11.08931
202388_at	RGS2	6.96E-09	−1.31366	8.785281	10.09894
220023_at	APOBR	7.38E-09	−0.74461	6.680544	7.425159
209304_x_at	GADD45B	7.82E-09	−0.81852	7.141328	7.959849
212657_s_at	IL1RN	8.52E-09	−1.67166	5.837259	7.508917
210992_x_at	FCGR2C	9.54E-09	−1.52335	5.51574	7.039091
1555950_a_at	CD55	9.79E-09	−0.63021	8.330255	8.960461
238546_at	SLC8A1	1.46E-08	−0.94304	4.776385	5.719425
1557197_a_at	LGALS3	1.47E-08	−0.65521	4.95092	5.606134
205627_at	CDA	1.48E-08	−1.39277	5.068627	6.461397
217388_s_at	KYNU	1.55E-08	−1.19344	6.92199	8.115426
1552542_s_at	TAGAP	1.79E-08	−0.7928	6.670254	7.463058
214467_at	GPR65	1.88E-08	−1.10152	6.157602	7.25912
211965_at	ZFP36L1	1.99E-08	−0.90835	5.44594	6.354291
227935_s_at	PCGF5	2.02E-08	−0.63159	7.227878	7.859468
226140_s_at	OTUD1	2.03E-08	−1.04128	7.926663	8.967943
204440_at	CD83	2.10E-08	−1.24882	7.762654	9.011471
222934_s_at	CLEC4E	2.20E-08	−1.22014	5.052891	6.273034
209288_s_at	CDC42EP3	2.24E-08	−1.0529	7.433675	8.486579
202877_s_at	CD93	2.35E-08	−1.25306	6.538736	7.791794
235670_at	STX11	2.42E-08	−1.18543	6.972295	8.157729
233031_at	ZEB2	2.50E-08	−0.90822	6.476011	7.384226
206130_s_at	ASGR2	2.51E-08	−1.25966	5.376179	6.635844
210754_s_at	LYN	2.71E-08	−0.59671	9.510818	10.10753
204385_at	KYNU	2.83E-08	−0.79775	6.246297	7.04405
211810_s_at	GALC	3.00E-08	−0.60075	6.063771	6.664521
231035_s_at	OTUD1	3.09E-08	−1.02103	5.610848	6.631881
219947_at	CLEC4A	3.16E-08	−1.29231	5.826819	7.119132
218611_at	IER5	3.26E-08	−0.78572	8.789276	9.575001
216841_s_at	SOD2	3.34E-08	−1.0432	6.824636	7.867832
1555214_a_at	CLEC7A	3.35E-08	−1.3783	4.780042	6.158347
203300_x_at	AP1S2	3.42E-08	−0.75643	9.132858	9.889292
205205_at	RELB	3.46E-08	−0.68687	6.289778	6.976648
216243_s_at	IL1RN	3.64E-08	−1.14942	5.639089	6.788514
204062_s_at	ULK2	3.68E-08	−0.6942	5.576796	6.270994
209367_at	STXBP2	3.84E-08	−0.61011	8.647205	9.257312
234050_at	TAGAP	3.95E-08	−0.96008	6.052788	7.012866

202626_s_at	LYN	3.97E-08	−0.6171	9.151568	9.768668
1555643_s_at	LILRA5	4.09E-08	−1.15432	5.538494	6.692813
1555326_a_at	ADAM9	4.16E-08	−0.83881	5.332349	6.171159
203761_at	SLA	4.77E-08	−0.68725	8.079888	8.767136
218376_s_at	MICAL1	4.83E-08	−0.84701	8.088334	8.935344
205681_at	BCL2A1	4.93E-08	−1.52817	6.813302	8.341475
215990_s_at	BCL6	5.45E-08	−1.21129	5.782328	6.993618
210959_s_at	SRD5A1	5.78E-08	−0.62148	5.282091	5.903574
209305_s_at	GADD45B	6.09E-08	−0.8641	6.515067	7.379163
203455_s_at	SAT1	6.34E-08	−0.6499	10.35173	11.00162
215078_at	SOD2	6.59E-08	−1.28716	4.83679	6.123946
206934_at	SIRPB1	7.38E-08	−0.9656	5.408461	6.374064
206420_at	IGSF6	7.51E-08	−1.13829	6.065865	7.204154
205992_s_at	IL15	7.55E-08	−0.72393	5.153424	5.877352
1566342_at	SOD2	7.98E-08	−0.87885	6.886385	7.76524
210663_s_at	KYNU	8.78E-08	−1.03103	6.622332	7.653364
215838_at	LILRA5	9.18E-08	−0.97892	5.638196	6.617117
226561_at	AGFG1	9.31E-08	−0.67509	7.225095	7.90019
209636_at	NFKB2	9.43E-08	−0.6606	5.060872	5.721469
211307_s_at	FCAR	1.03E-07	−1.15229	4.775218	5.927504
200784_s_at	LRP1	1.05E-07	−0.86045	4.808573	5.669023
210538_s_at	BIRC3	1.10E-07	−0.8237	6.00409	6.827787
226810_at	OGFRL1	1.12E-07	−0.84564	8.380204	9.22584
202637_s_at	ICAM1	1.21E-07	−1.22045	6.80207	8.022516
1561615_s_at	SLC8A1	1.23E-07	−0.70103	4.842858	5.543891
204790_at	SMAD7	1.29E-07	−0.85839	5.39045	6.248837
223454_at	CXCL16	1.32E-07	−0.96531	6.07554	7.040852
228745_at	SGTB	1.34E-07	−0.74376	6.197599	6.941354
232311_at	B2M	1.35E-07	−0.61975	6.637161	7.256908
221724_s_at	CLEC4A	1.37E-07	−1.09711	5.286589	6.383701
210422_x_at	SLC11A1	1.38E-07	−0.69852	6.666757	7.365279
1554624_a_at	SIRPB1	1.39E-07	−1.22661	5.936192	7.162799
60084_at	CYLD	1.51E-07	−0.62808	5.544914	6.172993
1552807_a_at	SIGLEC10	1.68E-07	−0.76494	7.149224	7.914162
217591_at	SKIL	1.76E-07	−0.88099	6.532587	7.413578
225142_at	JHDM1D	1.77E-07	−0.7311	5.526676	6.257775
222876_s_at	ADAP2	1.80E-07	−1.14001	5.298767	6.438779
211962_s_at	ZFP36L1	1.83E-07	−1.2531	7.818815	9.071913
36564_at	RNF19B	1.89E-07	−0.7628	7.49506	8.257861
203299_s_at	AP1S2	1.92E-07	−0.62371	7.150788	7.774498
219358_s_at	ADAP2	1.97E-07	−0.68132	6.242132	6.923448
202638_s_at	ICAM1	2.03E-07	−1.11509	6.281318	7.396413
240103_at	LOC100996457	2.03E-07	−0.91532	4.693468	5.608788
235518_at	SLC8A1	2.11E-07	−1.08239	5.461757	6.544151
210423_s_at	SLC11A1	2.21E-07	−1.25532	6.140434	7.39575

218454_at	PLBD1	2.23E-07	−1.79814	6.792698	8.590841
223422_s_at	ARHGAP24	2.34E-07	−0.62086	5.109768	5.730625
203561_at	FCGR2A	2.37E-07	−1.2935	6.811453	8.104952
230264_s_at	AP1S2	2.52E-07	−0.71064	9.37659	10.08723
201489_at	PPIF	2.56E-07	−0.6511	8.789337	9.440437
222496_s_at	RBM47	2.59E-07	−1.23378	6.389368	7.623144
207535_s_at	NFKB2	2.71E-07	−0.82595	7.171304	7.997256
202901_x_at	CTSS	2.73E-07	−0.88556	9.014888	9.900452
1554406_a_at	CLEC7A	2.85E-07	−1.04416	6.420867	7.465031
222218_s_at	PILRA	2.97E-07	−1.00981	7.583347	8.593154
204897_at	PTGER4	3.03E-07	−0.74143	9.605343	10.34678
227266_s_at	FYB	3.04E-07	−0.67104	8.640225	9.31127
227791_at	SLC9A9	3.06E-07	−0.73306	5.645476	6.378535
1557257_at	BCL10	3.08E-07	−0.61652	6.620867	7.237382
228176_at	S1PR3	3.13E-07	−0.85786	5.344698	6.202563
1554309_at	EIF4G3	3.15E-07	−0.62414	5.776866	6.401002
211395_x_at	FCGR2C	3.26E-07	−1.12008	6.887099	8.007182
204487_s_at	KCNQ1	3.50E-07	−0.94867	5.37464	6.323314
207674_at	FCAR	3.60E-07	−1.26762	5.717775	6.985394
209824_s_at	ARNTL	3.76E-07	−0.75484	5.709137	6.463972
220005_at	P2RY13	3.86E-07	−1.20047	5.919253	7.119718
204675_at	SRD5A1	4.26E-07	−0.65719	5.839724	6.496917
225059_at	AGTRAP	4.40E-07	−0.77299	7.721104	8.494095
219593_at	SLC15A3	4.45E-07	−1.39268	5.738743	7.131418
202625_at	LYN	4.59E-07	−0.59885	9.371046	9.969898
204907_s_at	BCL3	4.75E-07	−0.68279	5.733212	6.415998
1555167_s_at	NAMPT	4.77E-07	−1.39766	6.262248	7.659907
239704_at	RNF144B	4.85E-07	−1.03283	4.507283	5.540118
204393_s_at	ACPP	4.87E-07	−0.85553	5.562592	6.418122
211732_x_at	HNMT	4.88E-07	−0.95366	4.859064	5.812722
213038_at	RNF19B	5.27E-07	−0.67434	7.394295	8.068634
205686_s_at	CD86	5.35E-07	−0.9523	5.528831	6.48113
202912_at	ADM	5.37E-07	−1.12652	5.479787	6.606303
206244_at	CR1	5.43E-07	−1.03503	5.229302	6.264328
211816_x_at	FCAR	5.47E-07	−1.02428	5.515862	6.540139
202902_s_at	CTSS	5.50E-07	−0.97936	9.753022	10.73238
231990_at	USP15	5.52E-07	−0.68416	7.295686	7.979845
204122_at	TYROBP	5.56E-07	−0.80669	9.539269	10.34596
217911_s_at	BAG3	5.67E-07	−0.76272	5.518497	6.281222
212423_at	ZCCHC24	5.68E-07	−0.80905	5.220955	6.030001
215485_s_at	ICAM1	5.68E-07	−0.75752	5.84653	6.604046
205660_at	OASL	5.86E-07	−0.69363	5.641484	6.335114
213624_at	SMPDL3A	5.93E-07	−1.08063	5.219962	6.300593
205027_s_at	MAP3K8	6.07E-07	−0.80121	6.69513	7.496342
221477_s_at	SOD2	6.17E-07	−0.82875	8.543492	9.372242

205863_at	S100A12	6.30E-07	−1.72351	6.571822	8.295334
1568957_x_at	SRGAP2C	6.35E-07	−0.59098	5.819351	6.410332
211799_x_at	HLA-C	6.58E-07	−0.69968	10.11576	10.81544
228555_at	CAMK2D	6.66E-07	−0.70104	5.254539	5.955574
205859_at	LY86	6.78E-07	−1.09412	7.89071	8.984832
204118_at	CD48	6.83E-07	−1.00402	7.445389	8.449409
223169_s_at	RHOU	6.86E-07	−0.84729	5.359212	6.206502
209545_s_at	RIPK2	6.87E-07	−0.62365	8.44219	9.065837
201531_at	ZFP36	7.10E-07	−1.02413	9.884471	10.90861
208092_s_at	FAM49A	7.34E-07	−0.71494	6.50101	7.215948
202643_s_at	TNFAIP3	7.34E-07	−1.09321	8.832143	9.925351
204392_at	CAMK1	7.75E-07	−0.83778	5.460887	6.298665
232617_at	CTSS	7.92E-07	−0.88163	9.693856	10.57549
242903_at	IFNGR1	8.33E-07	−0.69972	7.777261	8.476984
202878_s_at	CD93	8.64E-07	−1.29578	7.821219	9.116999
221210_s_at	NPL	8.75E-07	−0.9538	5.749141	6.702937
213607_x_at	NADK	8.92E-07	−0.6174	7.003665	7.621063
204232_at	FCER1G	9.70E-07	−1.11908	9.420812	10.53989
218033_s_at	SNN	9.89E-07	−0.59355	5.15276	5.74631
203760_s_at	SLA	1.09E-06	−0.7675	8.133793	8.901297
213006_at	CEBPD	1.09E-06	−0.97087	5.503979	6.474848
211795_s_at	FYB	1.09E-06	−0.64612	8.539778	9.185897
219788_at	PILRA	1.15E-06	−1.07818	6.109465	7.187644
35626_at	SGSH	1.17E-06	−0.87933	6.257375	7.136707
222670_s_at	MAFB	1.19E-06	−1.532	7.523676	9.055673
221541_at	CRISPLD2	1.21E-06	−1.13365	6.288722	7.422373
200866_s_at	PSAP	1.21E-06	−0.77713	10.17251	10.94964
228486_at	SLC44A1	1.26E-06	−1.0614	5.953511	7.014908
200785_s_at	LRP1	1.31E-06	−0.69627	6.018892	6.715164
205568_at	AQP9	1.34E-06	−1.41653	5.953108	7.369639
207167_at	CD101	1.44E-06	−0.71379	5.493331	6.207121
211776_s_at	EPB41L3	1.45E-06	−1.02655	5.309051	6.335604
218793_s_at	SCML1	1.45E-06	−0.7122	4.744101	5.456305
202426_s_at	RXRA	1.54E-06	−0.65458	6.495135	7.149716
223019_at	FAM129B	1.56E-06	−0.76939	4.865918	5.635303
203922_s_at	CYBB	1.59E-06	−1.27376	6.955118	8.228877
217739_s_at	NAMPT	1.64E-06	−1.10399	8.860179	9.964167
225227_at	SKIL	1.67E-06	−0.66488	6.119882	6.784759
214211_at	FTH1	1.76E-06	−0.76853	9.031375	9.799907
244235_at	IVNS1ABP	1.80E-06	−0.64871	5.387436	6.03615
222142_at	CYLD	1.82E-06	−0.64107	5.108528	5.749596
211794_at	FYB	1.82E-06	−0.86	6.377998	7.237993
221561_at	SOAT1	1.84E-06	−0.62281	7.095955	7.718763
223217_s_at	NFKBIZ	1.93E-06	−0.84863	10.08626	10.93489
204735_at	PDE4A	2.04E-06	−0.70484	5.428903	6.133747

202644_s_at	TNFAIP3	2.04E-06	−0.91687	9.568376	10.48525
223767_at	GPR84	2.05E-06	−0.78246	4.939716	5.722177
205789_at	CD1D	2.18E-06	−1.37717	6.136262	7.513427
211530_x_at	HLA-G	2.19E-06	−0.65556	10.45635	11.11191
230550_at	MS4A6A	2.20E-06	−1.19825	6.19698	7.395231
207270_x_at	CD300C	2.30E-06	−0.67726	6.682798	7.360056
1552554_a_at	NLRC4	2.42E-06	−0.59883	4.875896	5.474723
214696_at	MIR22HG	2.46E-06	−0.72262	7.386122	8.108738
207691_x_at	ENTPD1	2.50E-06	−0.73187	6.216012	6.947883
201739_at	SGK1	2.52E-06	−1.11567	7.965596	9.081264
235549_at	RNF144B	2.76E-06	−0.62725	4.845492	5.472746
1555756_a_at	CLEC7A	2.77E-06	−1.49819	6.621652	8.119839
209473_at	ENTPD1	2.78E-06	−0.78294	6.452011	7.234955
202437_s_at	CYP1B1	2.82E-06	−1.27709	5.50877	6.785856
205118_at	FPR1	2.86E-06	−0.97083	5.084514	6.05534
203645_s_at	CD163	2.87E-06	−1.46303	5.936739	7.399773
1552504_a_at	BRSK1	2.92E-06	−0.85725	5.232927	6.090181
204417_at	GALC	2.95E-06	−0.71612	7.478105	8.19423
205639_at	AOAH	3.00E-06	−0.71195	7.314118	8.026063
206765_at	KCNJ2	3.13E-06	−0.91891	5.409905	6.328814
213524_s_at	G0S2	3.17E-06	−1.25669	6.724795	7.981487
223314_at	TSPAN14	3.18E-06	−0.61825	6.249812	6.868066
1552701_a_at	CARD16	3.37E-06	−0.80012	7.492271	8.292389
201743_at	CD14	3.40E-06	−1.93318	6.774038	8.707219
230499_at	BIRC3	3.55E-06	−0.6421	6.265827	6.90793
206710_s_at	EPB41L3	3.60E-06	−1.27345	5.149443	6.422895
210176_at	TLR1	3.63E-06	−0.66065	6.326044	6.986691
204588_s_at	SLC7A7	3.89E-06	−1.26021	7.157143	8.417352
219694_at	FAM105A	3.97E-06	−0.82006	5.414494	6.234551
209474_s_at	ENTPD1	4.26E-06	−0.8221	5.605993	6.428098
238987_at	B4GALT1	4.28E-06	−0.61551	7.070318	7.685828
223405_at	NPL	4.29E-06	−0.92799	6.397174	7.325159
204860_s_at	NAIP	4.32E-06	−0.72969	7.92203	8.651716
212831_at	MEGF9	4.41E-06	−0.76736	6.416314	7.183678
203140_at	BCL6	4.44E-06	−1.10355	8.047676	9.151225
1552772_at	CLEC4D	4.52E-06	−0.74695	4.848238	5.595184
228153_at	RNF144B	4.65E-06	−0.77889	7.392459	8.171347
225019_at	CAMK2D	4.66E-06	−0.76272	5.456191	6.218911
205922_at	VNN2	4.71E-06	−1.20984	5.777878	6.987721
213002_at	MARCKS	4.91E-06	−0.78651	6.150059	6.936573
230559_x_at	FGD4	5.06E-06	−0.6349	5.121403	5.756307
201473_at	JUNB	5.12E-06	−0.78651	9.514403	10.30091
1554899_s_at	FCER1G	5.16E-06	−1.25924	8.656191	9.915431
202381_at	ADAM9	5.34E-06	−0.77325	6.750733	7.523979
219872_at	FAM198B	5.35E-06	−0.99849	5.474299	6.472785

208018_s_at	HCK	5.41E-06	−0.97409	8.44515	9.419235
218559_s_at	MAFB	5.51E-06	−1.71557	7.038153	8.753719
235019_at	CPM	5.53E-06	−0.65001	5.187274	5.83728
201670_s_at	MARCKS	5.60E-06	−1.22683	6.542208	7.76904
205237_at	FCN1	5.72E-06	−1.6105	8.048907	9.659403
1555736_a_at	AGTRAP	5.79E-06	−0.81039	8.060549	8.870938
203234_at	UPP1	5.80E-06	−0.9252	6.052394	6.977593
37028_at	PPP1R15A	5.81E-06	−0.69684	9.343239	10.04008
225251_at	RAB24	5.83E-06	−0.60036	7.682737	8.283094
209803_s_at	PHLDA2	5.89E-06	−0.95638	5.128848	6.085232
217192_s_at	PRDM1	5.93E-06	−0.70816	5.214219	5.922379
1552773_at	CLEC4D	6.02E-06	−0.6577	5.169469	5.827166
217738_at	NAMPT	6.05E-06	−0.8733	7.952211	8.825511
212681_at	EPB41L3	6.11E-06	−1.22608	5.032693	6.258768
211676_s_at	IFNGR1	6.18E-06	−0.62224	8.466923	9.089158
211100_x_at	LILRA2	6.35E-06	−0.80763	7.633975	8.4416
204908_s_at	BCL3	6.42E-06	−0.71073	7.324265	8.034994
242445_at	FGD4	6.42E-06	−0.59984	5.47361	6.073446
215049_x_at	CD163	6.54E-06	−1.40634	5.798242	7.20458
228758_at	BCL6	6.55E-06	−0.95062	5.811113	6.761733
209606_at	CYTIP	7.11E-06	−0.70519	8.314164	9.01935
227131_at	MAP3K3	7.27E-06	−0.59888	6.499936	7.098814
221755_at	EHBP1L1	7.41E-06	−0.6533	7.028912	7.682216
1555638_a_at	SAMSN1	7.78E-06	−0.613	7.741011	8.354013
200998_s_at	CKAP4	7.81E-06	−0.71863	7.310629	8.029263
221578_at	RASSF4	7.83E-06	−0.85552	5.111782	5.967306
235568_at	C19orf59	7.83E-06	−0.94849	6.441482	7.38997
217507_at	SLC11A1	7.85E-06	−0.71582	5.014969	5.730785
207872_s_at	LILRA1	7.98E-06	−0.67044	5.988797	6.659238
201565_s_at	ID2	8.33E-06	−0.83673	9.187253	10.02398
224790_at	ASAP1	8.38E-06	−0.64404	6.854618	7.498654
1561880_a_at	SIGLEC16	8.76E-06	−0.68941	4.602686	5.292097
223423_at	GPR160	9.03E-06	−0.6199	7.211281	7.831176
1554676_at	SRGN	9.15E-06	−0.79835	8.679086	9.477435
214370_at	S100A8	9.25E-06	−0.93118	5.405071	6.336248
205786_s_at	ITGAM	9.37E-06	−0.95616	8.492231	9.448392
228485_s_at	SLC44A1	9.37E-06	−0.7082	6.225665	6.933862
208949_s_at	LGALS3	9.41E-06	−1.16991	7.887706	9.057614
225316_at	MFSD2A	9.55E-06	−0.67742	4.800489	5.477908
211102_s_at	LILRA2	9.69E-06	−0.97124	5.77578	6.747015
230748_at	SLC16A6	9.83E-06	−0.69904	5.244907	5.943944
229510_at	MS4A14	9.94E-06	−0.80156	5.691653	6.493213
202464_s_at	PFKFB3	1E-05	−0.8538	7.552453	8.406251
203045_at	NINJ1	1.01E-05	−1.00743	6.735748	7.743174
223944_at	NLRP12	1.01E-05	−0.81483	6.18364	6.998468

229770_at	GLT1D1	1.03E-05	−0.81803	4.899157	5.717183
209696_at	FBP1	1.04E-05	−1.04095	6.418708	7.459655
201631_s_at	IER3	1.08E-05	−1.25216	6.909173	8.161328
214590_s_at	UBE2D1	1.08E-05	−0.76344	6.51504	7.278481
203665_at	HMOX1	1.1E-05	−1.00137	6.369972	7.371347
211135_x_at	LILRB3	1.13E-05	−0.98897	6.961851	7.950818
201668_x_at	MARCKS	1.17E-05	−1.05663	5.387709	6.444338
229040_at	ITGB2-AS1	1.24E-05	−0.7421	6.062918	6.805017
206025_s_at	TNFAIP6	1.25E-05	−0.70599	5.258574	5.964561
209037_s_at	EHD1	1.31E-05	−0.62927	6.610432	7.239699
202436_s_at	CYP1B1	1.33E-05	−0.99245	5.92067	6.913118
210660_at	LILRA1	1.36E-05	−0.94603	6.474672	7.420703
207224_s_at	SIGLEC7	1.41E-05	−0.71717	6.002544	6.719717
202295_s_at	CTSH	1.42E-05	−1.24458	7.617637	8.862216
203932_at	HLA-DMB	1.44E-05	−0.7561	7.913442	8.669537
1568955_at	SRGAP2C	1.46E-05	−0.7362	6.400619	7.136817
213418_at	HSPA6	1.56E-05	−0.93327	5.876852	6.810122
210225_x_at	LILRB3	1.57E-05	−0.97386	7.041154	8.015017
204619_s_at	VCAN	1.58E-05	−1.60606	7.380619	8.986681
213537_at	HLA-DPA1	1.6E-05	−1.08272	6.908847	7.991571
220832_at	TLR8	1.61E-05	−1.11198	5.484286	6.596265
1560058_at	LOC399900	1.64E-05	−0.77205	5.80285	6.574901
201642_at	IFNGR2	1.66E-05	−0.6273	9.16784	9.795135
204614_at	SERPINB2	1.66E-05	−1.4332	6.143553	7.576754
232829_at	OR52K3P	1.66E-05	−0.59225	5.022545	5.614795
213475_s_at	ITGAL	1.66E-05	−0.62822	8.206249	8.834466
203186_s_at	S100A4	1.7E-05	−0.78131	10.07921	10.86052
205685_at	CD86	1.7E-05	−0.74935	6.118499	6.867844
210152_at	LILRB4	1.74E-05	−0.65166	6.219214	6.870871
1552553_a_at	NLRC4	1.75E-05	−0.73559	5.606278	6.341866
221698_s_at	CLEC7A	1.82E-05	−1.2864	6.786298	8.072696
207104_x_at	LILRB1	1.85E-05	−1.11277	5.820211	6.93298
203771_s_at	BLVRA	1.85E-05	−0.61501	4.792355	5.407365
206026_s_at	TNFAIP6	1.94E-05	−0.6511	5.137546	5.78865
202895_s_at	SIRPA	1.96E-05	−0.63558	5.751351	6.386926
229584_at	LRRK2	1.97E-05	−0.91419	5.703375	6.617567
224583_at	COTL1	1.97E-05	−0.87022	9.091989	9.96221
210184_at	ITGAX	1.98E-05	−0.68311	6.727046	7.410157
200871_s_at	PSAP	2E-05	−0.66182	10.76903	11.43086
220330_s_at	SAMSN1	2.1E-05	−0.61139	8.617966	9.229354
201566_x_at	ID2	2.11E-05	−0.80541	7.135174	7.940581
1568768_s_at	BRE-AS1	2.14E-05	−0.84584	5.654567	6.500409
212956_at	TBC1D9	2.18E-05	−0.90083	5.722473	6.6233
204081_at	NRGN	2.21E-05	−1.06566	7.101539	8.167199
202435_s_at	CYP1B1	2.24E-05	−0.81003	5.593685	6.403717

214511_x_at	FCGR1B	2.32E-05	−1.01988	7.136377	8.156262
214770_at	MSR1	2.4E-05	−0.78133	4.714736	5.496067
224130_s_at	SRA1	2.41E-05	−0.65997	5.948357	6.608332
205076_s_at	MTMR11	2.42E-05	−0.80539	5.647682	6.453074
203271_s_at	UNC119	2.43E-05	−0.68912	5.53657	6.225685
210773_s_at	FPR2	2.43E-05	−0.81826	5.307442	6.125706
210895_s_at	CD86	2.5E-05	−1.06499	6.57453	7.639518
206488_s_at	CD36	2.5E-05	−1.08108	8.298137	9.379218
235385_at	1-Mar	2.61E-05	−0.99471	5.873891	6.868599
228585_at	ENTPD1	2.66E-05	−0.75555	5.263351	6.0189
211429_s_at	SERPINA1	2.7E-05	−1.2975	8.778287	10.07579
223280_x_at	MS4A6A	2.76E-05	−1.23947	7.901938	9.141405
223922_x_at	MS4A6A	2.76E-05	−0.95441	7.970818	8.925228
203923_s_at	CYBB	2.78E-05	−1.16095	8.03604	9.196993
228325_at	KIAA0146	2.79E-05	−0.95192	6.165973	7.117897
211571_s_at	VCAN	2.88E-05	−1.73194	7.764091	9.496026
1554952_s_at	NLRP12	2.89E-05	−0.72165	5.60335	6.325001
210873_x_at	APOBEC3A	2.89E-05	−1.19904	6.613033	7.812076
202201_at	BLVRB	2.93E-05	−0.78697	7.620924	8.40789
204912_at	IL10RA	2.94E-05	−0.98267	7.817007	8.79968
230413_s_at	AP1S2	2.98E-05	−0.61298	7.370443	7.983427
216016_at	NLRP3	2.99E-05	−0.81562	4.613967	5.429584
221718_s_at	AKAP13	3E-05	−0.63343	7.883935	8.517365
227948_at	FGD4	3.02E-05	−0.79245	6.332788	7.125236
229067_at	SRGAP2C	3.21E-05	−0.70729	8.225042	8.93233
223218_s_at	NFKBIZ	3.22E-05	−0.6261	10.71832	11.34442
204182_s_at	ZBTB43	3.29E-05	−0.88338	5.895731	6.779108
1552501_a_at	GPBAR1	3.44E-05	−0.8026	5.269783	6.072382
211336_x_at	LILRB1	3.45E-05	−1.06219	6.218742	7.280934
212501_at	CEBPB	3.47E-05	−0.74643	10.20887	10.9553
211764_s_at	UBE2D1	3.53E-05	−0.61577	7.950042	8.56581
201669_s_at	MARCKS	3.57E-05	−1.2641	7.537512	8.801607
205936_s_at	HK3	3.61E-05	−1.41319	6.714821	8.128006
219403_s_at	HPSE	3.63E-05	−0.77618	5.218862	5.995044
1554999_at	RASGEF1B	3.69E-05	−0.7681	5.525195	6.293298
211824_x_at	NLRP1	3.76E-05	−0.67718	5.133579	5.810758
200782_at	ANXA5	3.97E-05	−1.27752	7.523022	8.800546
203060_s_at	PAPSS2	3.97E-05	−0.70326	5.388082	6.09134
1559034_at	SIRPB2	4.03E-05	−0.74936	6.953108	7.702464
207697_x_at	LILRB2	4.12E-05	−1.24701	6.49389	7.740903
202609_at	EPS8	4.26E-05	−0.73171	5.504756	6.236463
218035_s_at	RBM47	4.27E-05	−0.978	6.472486	7.450484
210785_s_at	THEMIS2	4.34E-05	−0.70153	8.580823	9.282349
244313_at	CR1	4.37E-05	−1.05377	6.308585	7.362352
235086_at	THBS1	4.52E-05	−0.80728	4.847723	5.655

222068_s_at	DNAAF1	4.61E-05	−0.64657	4.707505	5.354079
1556034_s_at	MTMR11	4.68E-05	−0.77743	4.962174	5.739605
202014_at	PPP1R15A	5.16E-05	−0.75645	8.776421	9.532874
1555728_a_at	MS4A4A	5.18E-05	−0.84995	5.902657	6.75261
227276_at	PLXDC2	5.27E-05	−0.59451	6.60407	7.198575
205119_s_at	FPR1	5.32E-05	−1.43279	7.190914	8.623701
209823_x_at	HLA-DQB1	5.33E-05	−1.06671	6.674233	7.740947
229221_at	CD44	5.41E-05	−0.67934	6.643912	7.323249
206756_at	CHST7	5.43E-05	−0.64526	4.812922	5.458178
209555_s_at	CD36	5.44E-05	−1.12224	8.406145	9.528385
243296_at	NAMPT	5.67E-05	−0.93135	9.766472	10.69782
208450_at	LGALS2	5.74E-05	−1.18378	5.385094	6.568872
225800_at	JAZF1	5.92E-05	−0.89916	6.2822	7.181363
205067_at	IL1B	6.03E-05	−0.99962	8.274988	9.274611
205207_at	IL6	6.2E-05	−0.9173	5.050341	5.967639
206513_at	AIM2	6.24E-05	−0.63766	5.619592	6.257251
219607_s_at	MS4A4A	6.42E-05	−0.9232	5.748297	6.671497
204863_s_at	IL6ST	6.48E-05	−0.60412	5.822549	6.426669
206157_at	PTX3	6.53E-05	−1.07597	6.619685	7.695656
211656_x_at	HLA-DQB1	6.74E-05	−0.8735	7.445865	8.319362
205099_s_at	CCR1	6.85E-05	−0.87405	6.847398	7.721446
219434_at	TREM1	6.87E-05	−1.02741	6.776773	7.804182
207571_x_at	THEMIS2	6.98E-05	−0.66294	8.331275	8.99421
233888_s_at	SRGAP1	7E-05	−0.66097	4.673486	5.334456
1554240_a_at	ITGAL	7.16E-05	−0.60553	8.987177	9.592707
204834_at	FGL2	7.35E-05	−1.23308	7.736882	8.96996
206881_s_at	LILRA3	7.39E-05	−0.89011	4.775068	5.665178
210279_at	GPR18	7.43E-05	−0.60417	5.582684	6.186857
219574_at	1-Mar	7.5E-05	−0.86893	6.057594	6.92652
243099_at	NFAM1	7.66E-05	−0.59795	6.499223	7.097171
203535_at	S100A9	7.77E-05	−1.67394	8.240565	9.914501
207237_at	KCNA3	7.78E-05	−0.64952	5.062312	5.711828
210904_s_at	IL13RA1	7.88E-05	−0.74699	6.187644	6.934634
206707_x_at	FAM65B	8.01E-05	−0.68535	8.156201	8.841551
217552_x_at	CR1	8.06E-05	−0.95561	5.897562	6.853175
39402_at	IL1B	8.14E-05	−0.97875	8.275233	9.253985
218950_at	ARAP3	8.18E-05	−0.62281	5.651049	6.273855
217362_x_at	HLA-DRB6	8.46E-05	−0.6408	7.612149	8.252953
215646_s_at	VCAN	8.64E-05	−1.70619	8.035602	9.741787
218217_at	SCPEP1	8.67E-05	−0.90891	7.943945	8.852856
224301_x_at	H2AFJ	9.17E-05	−0.73684	6.426209	7.163051
221345_at	FFAR2	9.49E-05	−0.70064	4.694839	5.395481
229041_s_at	ITGB2-AS1	9.5E-05	−0.80088	7.471026	8.271904
201850_at	CAPG	9.63E-05	−0.6686	8.253319	8.921919
207857_at	LILRA2	9.74E-05	−0.84022	7.651154	8.491374

207091_at	P2RX7	0.000104	−0.59379	5.198278	5.792065
208748_s_at	FLOT1	0.000105	−0.58914	4.951035	5.540174
220088_at	C5AR1	0.000106	−1.34484	7.154855	8.499696
208488_s_at	CR1	0.000106	−0.67983	6.303503	6.983332
229383_at	1-Mar	0.000107	−0.83857	6.753194	7.591762
205159_at	CSF2RB	0.000109	−0.67914	8.705814	9.384952
219666_at	MS4A6A	0.00011	−1.14474	7.58271	8.72745
212188_at	KCTD12	0.000112	−1.24071	7.675565	8.916278
211306_s_at	FCAR	0.000113	−0.66868	4.552549	5.221228
211612_s_at	IL13RA1	0.000114	−0.67334	6.471353	7.144689
203979_at	CYP27A1	0.000114	−0.6746	5.037475	5.712077
217763_s_at	RAB31	0.000115	−1.17496	6.861697	8.036652
229937_x_at	LILRB1	0.000115	−0.96316	6.403852	7.367009
225897_at	MARCKS	0.000117	−0.95023	6.115856	7.06609
210113_s_at	NLRP1	0.000118	−0.73952	5.354804	6.094323
210166_at	TLR5	0.000119	−0.62354	6.430758	7.054295
242814_at	SERPINB9	0.00012	−0.75538	7.098903	7.854286
217897_at	FXYD6	0.00012	−0.74113	5.540326	6.281455
215666_at	HLA-DRB4	0.000123	−0.86107	4.845589	5.706661
223816_at	SLC46A2	0.000126	−0.60355	4.595053	5.198599
214054_at	DOK2	0.000129	−0.72447	7.151688	7.876156
235818_at	VSTM1	0.00013	−0.84088	4.90095	5.741826
204858_s_at	TYMP	0.00013	−0.94484	6.57112	7.515963
229228_at	CREB5	0.000132	−0.71774	5.467084	6.184828
227995_at	LOC100509635	0.000133	−0.63466	5.382875	6.017532
225009_at	CMTM4	0.000133	−0.587	6.320541	6.90754
207704_s_at	GAS7	0.000135	−0.61763	6.733803	7.351436
49306_at	RASSF4	0.000135	−0.73447	6.048187	6.782662
206278_at	PTAFR	0.000137	−0.62633	5.561423	6.187752
219714_s_at	CACNA2D3	0.00014	−0.73284	5.142161	5.874998
205098_at	CCR1	0.000141	−0.97219	6.884819	7.857013
221042_s_at	CLMN	0.000145	−0.60689	4.938184	5.545073
239336_at	THBS1	0.000145	−0.76978	4.60792	5.377699
208540_x_at	S100A11	0.000148	−0.76145	8.58073	9.34218
223344_s_at	MS4A7	0.00015	−0.91279	6.790738	7.703533
201939_at	PLK2	0.00015	−0.72825	5.278722	6.006974
201109_s_at	THBS1	0.000152	−1.13896	5.429123	6.568087
211101_x_at	LILRA2	0.000161	−0.81655	7.42776	8.244313
228685_at	IL17RA	0.000161	−0.63929	6.756329	7.395616
225798_at	JAZF1	0.000161	−0.83163	6.74136	7.572986
224356_x_at	MS4A6A	0.000165	−1.17976	7.945364	9.12512
210146_x_at	LILRB2	0.000166	−1.08181	6.827963	7.909774
204759_at	RCBTB2	0.000172	−0.61523	7.005374	7.620602
206034_at	SERPINB8	0.000172	−0.69664	5.945838	6.642483
204319_s_at	RGS10	0.000176	−0.67685	7.066855	7.743704

202833_s_at	SERPINA1	0.000178	−1.4978	8.172471	9.670273
239012_at	RNF144B	0.000183	−0.62672	5.450904	6.077628
204620_s_at	VCAN	0.000183	−1.37792	8.85911	10.23703
208594_x_at	LILRA6	0.000184	−0.90206	6.85908	7.761138
206743_s_at	ASGR1	0.000184	−0.67772	4.435721	5.113444
218831_s_at	FCGRT	0.000186	−0.87677	7.152253	8.029026
218810_at	ZC3H12A	0.000189	−0.76687	6.894491	7.661363
238320_at	NEAT1	0.000191	−0.65665	8.909219	9.565869
200660_at	S100A11	0.0002	−0.94483	8.658157	9.602992
1559975_at	BTG1	0.000202	−0.65533	6.784144	7.439477
226841_at	MPEG1	0.000202	−1.35131	7.679872	9.031179
218319_at	PELI1	0.000208	−0.779	8.140044	8.91904
1559502_s_at	LRRC25	0.000208	−0.98164	6.603009	7.584647
226818_at	MPEG1	0.000208	−1.35946	8.338094	9.697555
204896_s_at	PTGER4	0.00021	−0.82134	6.021128	6.842472
217728_at	S100A6	0.000216	−0.83168	9.183363	10.01504
233555_s_at	SULF2	0.000216	−0.86619	5.817417	6.683604
232213_at	PELI1	0.000218	−0.71192	7.400108	8.112024
217818_s_at	ARPC4	0.000221	−0.72828	6.188056	6.916338
207113_s_at	TNF	0.000222	−0.92169	5.939994	6.861688
1559883_s_at	SAMHD1	0.000229	−0.85855	7.022946	7.8815
238893_at	LOC338758	0.000229	−0.79126	8.807861	9.59912
220532_s_at	TMEM176B	0.000233	−1.1299	6.504096	7.633991
203388_at	ARRB2	0.000238	−0.59975	8.86062	9.460374
212647_at	RRAS	0.000238	−0.6725	5.609571	6.282074
225626_at	PAG1	0.00024	−0.61913	6.792742	7.411871
204103_at	CCL4	0.000246	−1.15414	6.665001	7.819145
211367_s_at	CASP1	0.000249	−0.59993	7.918002	8.517933
202498_s_at	SLC2A3	0.000251	−0.73711	7.395765	8.132873
201888_s_at	IL13RA1	0.000252	−0.68823	5.818746	6.506976
235458_at	HAVCR2	0.000258	−0.72513	6.219569	6.944695
205241_at	SCO2	0.000278	−0.59105	7.469586	8.060631
202087_s_at	CTSL1	0.000278	−0.68649	6.776813	7.463305
241098_at	CLEC7A	0.000287	−0.68774	4.426574	5.114317
209791_at	PADI2	0.000288	−0.7508	6.027592	6.778397
235421_at	MAP3K8	0.000294	−0.61036	6.788226	7.398584
213716_s_at	SECTM1	0.000304	−0.86196	6.380836	7.242795
205819_at	MARCO	0.000305	−0.69749	4.582801	5.280286
203066_at	CHST15	0.000308	−1.02974	6.205673	7.235412
210772_at	FPR2	0.000309	−0.67274	5.428892	6.101635
225245_x_at	H2AFJ	0.00032	−0.59819	7.000515	7.598709
205476_at	CCL20	0.000324	−0.99598	4.463314	5.459295
242197_x_at	CD36	0.00033	−0.76182	5.288904	6.050724
212464_s_at	FN1	0.000333	−0.69565	4.655202	5.350852
210569_s_at	SIGLEC9	0.000371	−0.6021	4.486024	5.088122

224596_at	SLC44A1	0.000373	−0.62418	9.234417	9.858598
209684_at	RIN2	0.000386	−0.71192	5.981099	6.693017
213446_s_at	IQGAP1	0.000396	−0.60182	7.996199	8.598019
1554173_at	CD300LB	0.000404	−0.62775	5.665913	6.293659
212830_at	MEGF9	0.000418	−0.76521	7.872601	8.637812
206359_at	SOCS3	0.000428	−0.61291	5.967657	6.580566
208438_s_at	FGR	0.000433	−0.96985	8.535822	9.505676
221731_x_at	VCAN	0.000449	−1.39521	8.890808	10.28601
229101_at	IL17RA	0.00045	−0.62829	8.190233	8.818527
202340_x_at	NR4A1	0.000457	−0.78476	6.114019	6.898778
201108_s_at	THBS1	0.000459	−0.85074	5.529849	6.380585
202497_x_at	SLC2A3	0.000467	−0.72853	8.227928	8.956462
204057_at	IRF8	0.000478	−0.78304	8.259723	9.042767
201110_s_at	THBS1	0.000489	−1.04051	5.944678	6.985191
232724_at	MS4A6A	0.00049	−0.67048	5.586548	6.257027
224358_s_at	MS4A7	0.000502	−0.76674	6.224173	6.990916
1553906_s_at	FGD2	0.000502	−0.71466	8.125548	8.840204
211719_x_at	FN1	0.000509	−0.74892	4.649568	5.398489
211661_x_at	PTAFR	0.000516	−0.6659	7.226701	7.892603
210495_x_at	FN1	0.000519	−0.72246	4.787968	5.510431
209949_at	NCF2	0.000529	−1.21014	7.988664	9.198807
207275_s_at	ACSL1	0.00054	−0.74514	8.410457	9.155596
212196_at	IL6ST	0.000542	−0.6044	6.400657	7.005057
200999_s_at	CKAP4	0.00055	−0.80934	6.829627	7.638971
234985_at	LDLRAD3	0.00055	−0.76083	5.998618	6.759452
204655_at	CCL5	0.000555	−0.5881	7.774449	8.362553
229560_at	TLR8	0.000627	−1.03396	7.075289	8.109247
203973_s_at	CEBPD	0.000629	−0.93734	9.321231	10.25857
209581_at	PLA2G16	0.000635	−0.66916	4.840749	5.509906
1405_i_at	CCL5	0.000685	−0.78226	7.400067	8.182323
218345_at	TMEM176A	0.000687	−0.9117	6.458347	7.370047
205715_at	BST1	0.000687	−0.73	6.10296	6.832957
1567628_at	CD74	0.000688	−0.78	8.537264	9.317263
219890_at	CLEC5A	0.000736	−0.7957	5.614784	6.410487
220146_at	TLR7	0.000756	−0.67276	5.195141	5.867904
205174_s_at	QPCT	0.000758	−0.60935	4.98143	5.590784
1555349_a_at	ITGB2	0.000763	−0.60666	10.49587	11.10253
209728_at	HLA-DRB4	0.000774	−1.53138	6.021607	7.552982
226722_at	FAM20C	0.000796	−0.61659	5.380696	5.997284
200766_at	CTSD	0.000803	−0.61151	8.521239	9.132748
203508_at	TNFRSF1B	0.000823	−0.74207	7.711185	8.453256
211924_s_at	PLAUR	0.000828	−0.80027	8.09948	8.899753
217762_s_at	RAB31	0.000838	−1.00556	7.139975	8.145532
207072_at	IL18RAP	0.000846	−0.67523	5.375525	6.050755
202859_x_at	IL8	0.000848	−0.99537	9.826633	10.822

219505_at	CECR1	0.00093	−0.84808	8.936696	9.784774
208894_at	HLA-DRA	0.000936	−0.86141	10.52419	11.3856
201107_s_at	THBS1	0.001	−0.61208	4.494745	5.106824
206940_s_at	POU4F1	0.001009	−0.67459	4.94603	5.620615
204502_at	SAMHD1	0.001011	−0.9438	7.80657	8.750371
201373_at	PLEC	0.001017	−0.59619	5.968115	6.564307
202499_s_at	SLC2A3	0.001018	−0.64223	8.346703	8.988937
235306_at	GIMAP8	0.001026	−0.65274	6.04302	6.695758
211506_s_at	IL8	0.00105	−1.08075	8.402024	9.482774
208981_at	PECAM1	0.001067	−0.61655	9.336252	9.952805
219093_at	PID1	0.001097	−0.6196	4.843722	5.463322
211991_s_at	HLA-DPA1	0.001098	−0.94602	9.149905	10.09593
214366_s_at	ALOX5	0.001102	−0.72378	6.137406	6.861182
201163_s_at	IGFBP7	0.001107	−0.5873	9.360844	9.94814
211990_at	HLA-DPA1	0.001112	−0.79313	10.45658	11.24971
200838_at	CTSB	0.001116	−0.61906	9.46918	10.08824
227396_at	PTPRJ	0.001151	−0.73743	7.062924	7.800358
1563088_a_at	LOC284837	0.001185	−0.64354	5.692539	6.336082
225175_s_at	SLC44A2	0.001193	−0.59343	5.907763	6.501194
213566_at	RNASE6	0.001207	−1.06964	7.249108	8.318752
201422_at	IFI30	0.001233	−1.19417	8.844132	10.03831
228532_at	C1orf162	0.001238	−0.64406	8.809535	9.453597
223343_at	MS4A7	0.00127	−0.89264	8.089348	8.981992
232304_at	PELI1	0.001278	−0.68864	7.177742	7.866379
224724_at	SULF2	0.001323	−0.85376	7.017951	7.871715
212192_at	KCTD12	0.00142	−1.08655	7.876868	8.963421
201963_at	ACSL1	0.00144	−0.63659	9.145901	9.782489
208983_s_at	PECAM1	0.001445	−0.63539	8.302681	8.93807
209683_at	FAM49A	0.001458	−0.58891	6.214303	6.803208
231124_x_at	LY9	0.001504	−0.58714	5.975968	6.563105
232068_s_at	TLR4	0.001538	−0.77163	6.097734	6.869366
226436_at	RASSF4	0.001543	−0.79604	7.24909	8.045128
203773_x_at	BLVRA	0.001582	−0.59364	6.613156	7.206793
205220_at	HCAR3	0.001585	−0.8668	6.534194	7.400998
219279_at	DOCK10	0.001704	−0.70591	6.962113	7.668022
210982_s_at	HLA-DRA	0.001705	−0.92008	10.16277	11.08285
218232_at	C1QA	0.001837	−0.76943	5.383984	6.153414
211474_s_at	SERPINB6	0.001871	−0.5857	8.20934	8.795043
1555759_a_at	CCL5	0.001883	−0.6313	7.10553	7.736834
211372_s_at	IL1R2	0.001922	−0.69334	4.815358	5.508697
217764_s_at	RAB31	0.001936	−0.84836	7.926955	8.775314
205249_at	EGR2	0.001975	−0.67822	5.610348	6.28857
204794_at	DUSP2	0.002052	−0.72594	6.616834	7.342773
220066_at	NOD2	0.002133	−0.6605	6.066724	6.72722
224277_at	MOP-1	0.002134	−0.59906	4.420287	5.019348

201162_at	IGFBP7	0.002155	−0.69115	7.862872	8.554023
213241_at	PLXNC1	0.002163	−0.6077	7.854594	8.462294
201044_x_at	DUSP1	0.002214	−0.64593	7.958744	8.604672
223204_at	FAM198B	0.002249	−0.91262	7.277875	8.190496
202007_at	NID1	0.002298	−0.89467	5.469988	6.364657
212998_x_at	HLA-DQB1	0.002303	−1.05093	7.460208	8.511142
202687_s_at	TNFSF10	0.002351	−0.6171	6.511251	7.128356
228056_s_at	NAPSB	0.002448	−0.71617	5.308653	6.024826
205403_at	IL1R2	0.002582	−0.73608	5.306744	6.042823
201811_x_at	SH3BP5	0.002584	−0.60026	7.366324	7.966588
204787_at	VSIG4	0.002671	−0.64809	5.746116	6.394201
204621_s_at	NR4A2	0.002902	−0.68889	8.063423	8.75231
212993_at	NACC2	0.002945	−0.63739	6.267435	6.904827
219243_at	GIMAP4	0.002945	−0.70764	5.888597	6.596233
209616_s_at	CES1	0.002986	−0.75488	5.389587	6.144468
201506_at	TGFBI	0.003107	−0.82839	5.670766	6.499152
223168_at	RHOU	0.003214	−0.63073	6.37449	7.005215
228094_at	AMICA1	0.003217	−0.74063	7.202711	7.943337
211302_s_at	PDE4B	0.003272	−0.66829	7.407099	8.075385
216015_s_at	NLRP3	0.003379	−0.67504	6.360279	7.035315
210512_s_at	VEGFA	0.003528	−0.63984	6.572391	7.212233
204470_at	CXCL1	0.00359	−0.58722	5.41446	6.001683
201393_s_at	IGF2R	0.003715	−0.75513	6.924197	7.679332
220091_at	SLC2A6	0.003819	−0.62414	5.928607	6.552745
206380_s_at	CFP	0.003839	−0.73356	7.915656	8.649216
1554997_a_at	PTGS2	0.00391	−0.81902	6.011836	6.830856
227265_at	FGL2	0.004095	−0.94602	8.606341	9.552365
220266_s_at	KLF4	0.004319	−0.7093	7.442947	8.152251
214329_x_at	TNFSF10	0.004695	−0.65267	6.414328	7.067
210845_s_at	PLAUR	0.004742	−0.67052	8.740113	9.410634
202284_s_at	CDKN1A	0.005008	−0.60756	7.377775	7.98533
227184_at	PTAFR	0.00513	−0.59504	8.473261	9.0683
204112_s_at	HNMT	0.005173	−0.77009	6.111453	6.881541
217143_s_at	YME1L1	0.00552	−0.59081	5.868867	6.459676
204748_at	PTGS2	0.005545	−0.77941	7.028158	7.807568
228766_at	CD36	0.005605	−0.77742	8.32487	9.102287
202510_s_at	TNFAIP2	0.005633	−0.58812	8.390278	8.978402
202917_s_at	S100A8	0.005681	−1.06114	10.0832	11.14433
207794_at	CCR2	0.005847	−0.61884	6.040878	6.659723
211864_s_at	MYOF	0.005983	−0.6564	5.821984	6.478385
221558_s_at	LEF1	0.006079	−0.71325	6.380833	7.09408
200872_at	S100A10	0.006614	−0.65685	9.619589	10.27644
216248_s_at	NR4A2	0.006743	−0.68193	8.014288	8.696218
201137_s_at	HLA-DPB1	0.006849	−0.69009	9.590469	10.28056
202688_at	TNFSF10	0.00696	−0.64772	7.494082	8.141799

204622_x_at	NR4A2	0.0071	−0.6741	8.087097	8.761199
203708_at	PDE4B	0.007132	−0.71694	7.571509	8.288454
231577_s_at	GBP1	0.008655	−0.65048	6.188489	6.838967
202391_at	BASP1	0.009813	−0.80116	7.876467	8.677627
204141_at	TUBB2A	0.010032	−0.65912	6.097405	6.75652
210427_x_at	ANXA2	0.01026	−0.60813	9.403321	10.01145
209774_x_at	CXCL2	0.010609	−0.77181	8.014726	8.78654
235964_x_at	SAMHD1	0.011765	−0.7287	8.789307	9.518008
201798_s_at	MYOF	0.013034	−0.66839	6.626148	7.29454
201590_x_at	ANXA2	0.013458	−0.59642	9.406298	10.00271
202820_at	AHR	0.014847	−0.59056	8.279072	8.869627
203290_at	HLA-DQA1	0.015291	−0.60598	5.436743	6.042724
213503_x_at	ANXA2	0.015777	−0.6056	9.287027	9.89263
228055_at	NAPSB	0.016381	−0.69557	6.310889	7.006464
234987_at	SAMHD1	0.017	−0.6921	9.096218	9.788319
200762_at	DPYSL2	0.017366	−0.60526	8.267419	8.872677
207850_at	CXCL3	0.021428	−0.65848	6.857597	7.516078
206978_at	CCR2	0.023224	−0.62185	6.718405	7.340259
205557_at	BPI	0.025467	−0.66681	5.923219	6.590024
204446_s_at	ALOX5	0.02558	−0.60971	8.132672	8.742377
235529_x_at	SAMHD1	0.026019	−0.59524	9.312416	9.90766
216834_at	RGS1	0.029001	−0.62085	6.626317	7.247171
1555745_a_at	LYZ	0.02939	−0.70725	10.66354	11.37078

Supplementary Table S3: Correlation between *RUNX1* expression and microRNA profiles using whole-genome high-throughput sequencing

microRNA symbol	Correlation Coefficient	*P*-value	False Discover Rate
hsa-mir-16-2.MIMAT0004518	0.561794	7.17E-08	4.68E-05
hsa-mir-146b.MIMAT0002809	0.539189	2.95E-07	9.62E-05
hsa-mir-146b.MIMAT0004766	0.53209	4.50E-07	9.79E-05
hsa-mir-16.MIMAT0000069	0.482745	6.62E-06	0.00108
hsa-mir-196b.MIMAT0001080	0.475145	9.66E-06	0.001261
hsa-mir-15b.MIMAT0000417	0.462283	1.8E-05	0.001954
hsa-mir-550.MIMAT0004800	0.456991	2.3E-05	0.002145
hsa-mir-1249.MIMAT0005901	−0.44456	4.05E-05	0.00311
hsa-mir-940.MIMAT0004983	−0.4433	4.29E-05	0.00311
hsa-let-7a.MIMAT0000062	0.42284	0.000104	0.006147
hsa-mir-193a.MIMAT0004614	−0.42424	9.76E-05	0.006147
hsa-mir-320c.MIMAT0005793	0.41689	0.000132	0.007207
hsa-mir-125a.MIMAT0000443	0.400943	0.00025	0.012583
hsa-let-7e.MIMAT0000066	0.393968	0.000328	0.01261
hsa-let-7e.MIMAT0004485	0.39366	0.000332	0.01261
hsa-mir-1307.MIMAT0005951	−0.39776	0.000283	0.01261
hsa-mir-133a.MIMAT0000427	0.390996	0.000367	0.01261
hsa-mir-22.MIMAT0000077	−0.39675	0.000295	0.01261
hsa-mir-23a.MIMAT0000078	−0.39171	0.000357	0.01261
hsa-mir-92a-1.MIMAT0004507	0.377269	0.000609	0.019899
hsa-mir-1289.MIMAT0005879	0.369158	0.000814	0.024166
hsa-mir-876.MIMAT0004924	0.369158	0.000814	0.024166
hsa-mir-551b.MIMAT0003233	0.366498	0.000894	0.025377
hsa-let-7d.MIMAT0000065	0.361157	0.001076	0.028822
hsa-mir-1.MIMAT0000416	0.36041	0.001103	0.028822
hsa-mir-1323.MIMAT0005795	0.353902	0.001376	0.028985
hsa-mir-135a-1.MIMAT0004595	0.354976	0.001327	0.028985
hsa-mir-15b.MIMAT0004586	0.355271	0.001314	0.028985
hsa-mir-200c.MIMAT0000617	−0.35441	0.001353	0.028985
hsa-mir-30b.MIMAT0000420	0.35445	0.001351	0.028985
hsa-mir-600.MIMAT0003268	0.353902	0.001376	0.028985
hsa-mir-582.MIMAT0004797	−0.34991	0.001572	0.032081
hsa-mir-576.MIMAT0003241	0.345916	0.001793	0.035477
hsa-mir-132.MIMAT0000426	−0.345	0.001847	0.035482
hsa-mir-132.MIMAT0004594	−0.34051	0.002137	0.037707
hsa-mir-133b.MIMAT0000770	0.340571	0.002132	0.037707
hsa-mir-2110.MIMAT0010133	−0.34135	0.002079	0.037707
hsa-mir-187.MIMAT0000262	−0.33489	0.002555	0.042416
hsa-mir-22.MIMAT0004495	−0.33449	0.002588	0.042416
hsa-mir-651.MIMAT0003321	0.334358	0.002598	0.042416
hsa-mir-99b.MIMAT0000689	0.333352	0.002682	0.042712
hsa-mir-616.MIMAT0003284	−0.33235	0.002767	0.04302

hsa-mir-21.MIMAT0000076	−0.32892	0.003079	0.046758
hsa-mir-193a.MIMAT0000459	−0.32552	0.003419	0.050736
hsa-mir-501.MIMAT0004774	−0.3245	0.003527	0.051178
hsa-mir-326.MIMAT0000756	−0.32119	0.003899	0.055347
hsa-mir-550.MIMAT0003257	0.320172	0.004021	0.055864
hsa-mir-1976.MIMAT0009451	−0.31591	0.004567	0.06213
hsa-mir-1179.MIMAT0005824	0.311755	0.005161	0.06601
hsa-mir-320b.MIMAT0005792	0.311609	0.005183	0.06601
hsa-mir-320d.MIMAT0006764	0.312624	0.005031	0.06601
hsa-mir-374b.MIMAT0004955	0.311124	0.005257	0.06601
hsa-mir-1266.MIMAT0005920	0.309796	0.005464	0.066071
hsa-mir-30b.MIMAT0004589	0.31013	0.005411	0.066071
hsa-mir-720.MIMAT0005954	0.308327	0.005701	0.06769
hsa-mir-155.MIMAT0000646	0.30645	0.006018	0.068977
hsa-mir-624.MIMAT0003293	0.306433	0.006021	0.068977
hsa-mir-103.MIMAT0000101	−0.3055	0.006184	0.069619
hsa-mir-1228.MIMAT0005582	−0.30381	0.00649	0.071826
hsa-mir-152.MIMAT0000438	−0.30301	0.006639	0.072249
hsa-mir-99b.MIMAT0004678	0.302174	0.006799	0.072778
hsa-mir-449a.MIMAT0001541	0.295665	0.008158	0.085919
hsa-mir-508.MIMAT0002880	0.294437	0.008439	0.087475
hsa-mir-185.MIMAT0004611	−0.29316	0.008742	0.089195
hsa-mir-1250.MIMAT0005902	−0.29169	0.0091	0.091423
hsa-mir-140.MIMAT0004597	−0.29033	0.009445	0.092053
hsa-mir-196b.MIMAT0009201	0.290748	0.009337	0.092053
hsa-mir-582.MIMAT0003247	−0.28932	0.009706	0.093211
hsa-mir-125a.MIMAT0004602	0.287194	0.01028	0.093237
hsa-mir-378.MIMAT0000732	−0.28722	0.010272	0.093237
hsa-mir-484.MIMAT0002174	−0.28774	0.01013	0.093237
hsa-mir-877.MIMAT0004949	−0.28781	0.01011	0.093237
hsa-mir-491.MIMAT0002807	0.282895	0.011531	0.101753
hsa-mir-607.MIMAT0003275	0.28291	0.011526	0.101753
hsa-mir-500.MIMAT0002871	−0.2807	0.01222	0.106392
hsa-let-7b.MIMAT0004482	0.279636	0.012566	0.107964
hsa-mir-185.MIMAT0000455	−0.27905	0.012758	0.108198
hsa-mir-219-1.MIMAT0004567	0.273578	0.014704	0.123098
hsa-let-7f-1.MIMAT0004486	0.27133	0.015574	0.127122
hsa-mir-362.MIMAT0000705	−0.27174	0.015412	0.127122
hsa-let-7a.MIMAT0004481	0.268414	0.016768	0.135179
hsa-mir-19b-1.MIMAT0004491	0.266058	0.017789	0.141665
hsa-mir-223.MIMAT0000280	−0.26473	0.018389	0.144676
hsa-mir-103-2.MIMAT0009196	−0.26043	0.020447	0.154392
hsa-mir-130a.MIMAT0004593	0.260744	0.020292	0.154392
hsa-mir-192.MIMAT0004543	0.260488	0.020419	0.154392
hsa-mir-548k.MIMAT0005882	0.260189	0.02057	0.154392

hsa-mir-155.MIMAT0004658	0.258245	0.021569	0.160052
hsa-mir-130a.MIMAT0000425	0.257085	0.022185	0.160963
hsa-mir-361.MIMAT0000703	0.257294	0.022073	0.160963
hsa-mir-1538.MIMAT0007400	−0.25615	0.022693	0.161074
hsa-mir-629.MIMAT0003298	0.256554	0.022472	0.161074
hsa-mir-25.MIMAT0000081	0.243254	0.030758	0.215971
hsa-mir-212.MIMAT0000269	−0.24138	0.032108	0.223052
hsa-mir-551a.MIMAT0003214	0.239394	0.033598	0.23094
hsa-mir-107.MIMAT0000104	−0.23856	0.03424	0.232092
hsa-mir-24.MIMAT0000080	−0.23817	0.034541	0.232092
hsa-mir-589.MIMAT0004799	−0.2378	0.034832	0.232092
hsa-mir-532.MIMAT0002888	−0.23633	0.036002	0.237467
hsa-mir-10a.MIMAT0004555	0.235879	0.036371	0.237505
hsa-mir-1234.MIMAT0005589	0.235108	0.037005	0.239247
hsa-mir-24-2.MIMAT0004497	−0.23262	0.039112	0.247965
hsa-mir-574.MIMAT0003239	−0.23303	0.03876	0.247965
hsa-mir-135a.MIMAT0000428	0.230895	0.040631	0.254588
hsa-mir-30d.MIMAT0004551	0.230554	0.040937	0.254588
hsa-mir-149.MIMAT0000450	−0.22969	0.041716	0.256987
hsa-let-7f.MIMAT0000067	0.222385	0.048857	0.296562
hsa-mir-584.MIMAT0003249	0.222201	0.049049	0.296562
hsa-mir-30d.MIMAT0000245	0.220067	0.051321	0.307457
hsa-mir-514.MIMAT0002883	0.218682	0.052841	0.310859
hsa-mir-708.MIMAT0004927	−0.21877	0.052748	0.310859
hsa-mir-642.MIMAT0003312	−0.21775	0.053883	0.314155
hsa-mir-376b.MIMAT0002172	−0.21651	0.055305	0.319592
hsa-let-7g.MIMAT0000414	0.213108	0.059338	0.336936
hsa-mir-502.MIMAT0004775	−0.21324	0.059172	0.336936
hsa-mir-221.MIMAT0004568	−0.21183	0.06092	0.340424
hsa-mir-92a-2.MIMAT0004508	0.211767	0.060995	0.340424
hsa-mir-1224.MIMAT0005458	0.21084	0.062161	0.343993
hsa-let-7b.MIMAT0000063	0.209575	0.063783	0.350001
hsa-mir-490.MIMAT0002806	0.208499	0.065189	0.354735
hsa-mir-190.MIMAT0000458	0.207349	0.066718	0.360057
hsa-mir-616.MIMAT0004805	−0.20692	0.067293	0.360181
hsa-mir-513a.MIMAT0002877	0.206411	0.067988	0.360944
hsa-mir-874.MIMAT0004911	0.20342	0.072166	0.380037
hsa-mir-106b.MIMAT0000680	0.201271	0.075294	0.387142
hsa-mir-548n.MIMAT0005916	0.201525	0.074918	0.387142
hsa-mir-92a.MIMAT0000092	0.201881	0.074396	0.387142
hsa-mir-1914.MIMAT0007889	−0.19952	0.077924	0.397534
hsa-mir-151.MIMAT0004697	0.197602	0.080883	0.409432
hsa-mir-106b.MIMAT0004672	0.195422	0.084357	0.418586
hsa-mir-130b.MIMAT0000691	0.19511	0.084863	0.418586
hsa-mir-27b.MIMAT0004588	0.194901	0.085205	0.418586

hsa-mir-378.MIMAT0000731	−0.19487	0.085256	0.418586
hsa-mir-26a.MIMAT0000082	0.192372	0.089416	0.435735
hsa-mir-144.MIMAT0004600	0.190282	0.093018	0.443364
hsa-mir-216b.MIMAT0004959	0.190596	0.09247	0.443364
hsa-mir-589.MIMAT0003256	−0.19061	0.09244	0.443364
hsa-mir-194.MIMAT0000460	0.189669	0.094096	0.44525
hsa-mir-548d.MIMAT0003323	0.18925	0.094838	0.445532
hsa-mir-126.MIMAT0000445	0.187599	0.097808	0.454194
hsa-mir-744.MIMAT0004945	−0.18745	0.098073	0.454194
hsa-mir-26a-1.MIMAT0004499	0.186657	0.099536	0.457724
hsa-mir-496.MIMAT0002818	−0.18618	0.100413	0.458529
hsa-mir-1275.MIMAT0005929	0.184779	0.103049	0.4673
hsa-mir-374a.MIMAT0000727	0.184309	0.103943	0.468104
hsa-mir-188.MIMAT0004613	−0.18339	0.105708	0.469893
hsa-mir-21.MIMAT0004494	−0.18335	0.10578	0.469893
hsa-mir-1228.MIMAT0005583	−0.18223	0.107967	0.476369
hsa-mir-296.MIMAT0000690	−0.18077	0.110875	0.485916
hsa-mir-1226.MIMAT0005577	−0.17876	0.114977	0.490262
hsa-mir-1254.MIMAT0005905	0.178488	0.115529	0.490262
hsa-mir-27a.MIMAT0000084	−0.17844	0.115621	0.490262
hsa-mir-34c.MIMAT0004677	−0.17864	0.115211	0.490262
hsa-mir-636.MIMAT0003306	0.178493	0.11552	0.490262
hsa-mir-345.MIMAT0000772	0.175605	0.121626	0.512397
hsa-mir-10a.MIMAT0000253	0.174952	0.12304	0.515031
hsa-mir-139.MIMAT0000250	−0.17135	0.131073	0.530394
hsa-mir-141.MIMAT0000432	−0.16972	0.134832	0.530394
hsa-mir-215.MIMAT0000272	−0.17214	0.129278	0.530394
hsa-mir-26a-2.MIMAT0004681	0.172007	0.129575	0.530394
hsa-mir-423.MIMAT0004748	0.17167	0.130338	0.530394
hsa-mir-508.MIMAT0004778	0.170547	0.13291	0.530394
hsa-mir-611.MIMAT0003279	0.170103	0.133936	0.530394
hsa-mir-628.MIMAT0004809	−0.1699	0.134406	0.530394
hsa-mir-7-1.MIMAT0004553	0.169758	0.134738	0.530394
hsa-mir-765.MIMAT0003945	−0.17215	0.129241	0.530394
hsa-mir-493.MIMAT0003161	−0.16894	0.136663	0.531197
hsa-mir-506.MIMAT0002878	0.169062	0.13637	0.531197
hsa-mir-188.MIMAT0000457	−0.16831	0.13815	0.533799
hsa-mir-181d.MIMAT0002821	0.16703	0.141214	0.5415
hsa-mir-489.MIMAT0002805	−0.16662	0.142195	0.5415
hsa-mir-625.MIMAT0004808	0.166445	0.142631	0.5415
hsa-mir-572.MIMAT0003237	−0.16592	0.143906	0.543182
hsa-mir-937.MIMAT0004980	−0.16552	0.144892	0.543763
hsa-mir-641.MIMAT0003311	0.165102	0.145927	0.544516
hsa-mir-513c.MIMAT0005789	0.164696	0.146936	0.545166
hsa-mir-1915.MIMAT0007892	0.16371	0.149404	0.548094

hsa-mir-216a.MIMAT0000273	0.164021	0.148622	0.548094
hsa-mir-101-1.MIMAT0004513	0.162644	0.152109	0.554901
hsa-let-7g.MIMAT0004584	0.159002	0.161621	0.583087
hsa-mir-1913.MIMAT0007888	0.159027	0.161555	0.583087
hsa-mir-29c.MIMAT0004673	0.157208	0.166468	0.596698
hsa-mir-548f.MIMAT0005895	−0.15693	0.167222	0.596698
hsa-mir-663b.MIMAT0005867	−0.15558	0.170959	0.60672
hsa-mir-1271.MIMAT0005796	0.15463	0.173616	0.612817
hsa-mir-1225.MIMAT0005572	−0.15294	0.178413	0.623014
hsa-mir-885.MIMAT0004947	−0.15316	0.177798	0.623014
hsa-mir-1255a.MIMAT0005906	−0.1512	0.183478	0.624015
hsa-mir-183.MIMAT0000261	−0.15215	0.180694	0.624015
hsa-mir-184.MIMAT0000454	0.151519	0.182539	0.624015
hsa-mir-320a.MIMAT0000510	0.151527	0.182516	0.624015
hsa-mir-501.MIMAT0002872	−0.15182	0.181649	0.624015
hsa-mir-30c-2.MIMAT0004550	−0.15048	0.185583	0.627658
hsa-mir-877.MIMAT0004950	−0.15018	0.186471	0.627658
hsa-mir-18a.MIMAT0002891	0.149859	0.187434	0.627665
hsa-mir-425.MIMAT0003393	−0.14942	0.188756	0.628864
hsa-mir-30a.MIMAT0000088	−0.14834	0.192003	0.636437
hsa-mir-190b.MIMAT0004929	−0.14317	0.208132	0.642096
hsa-mir-20a.MIMAT0004493	0.14566	0.200241	0.642096
hsa-mir-211.MIMAT0000268	−0.14638	0.198003	0.642096
hsa-mir-217.MIMAT0000274	0.147136	0.195671	0.642096
hsa-mir-218.MIMAT0000275	−0.143	0.208658	0.642096
hsa-mir-29a.MIMAT0004503	−0.14297	0.208784	0.642096
hsa-mir-31.MIMAT0004504	0.146061	0.198993	0.642096
hsa-mir-330.MIMAT0000751	−0.14441	0.204173	0.642096
hsa-mir-340.MIMAT0004692	−0.14279	0.209351	0.642096
hsa-mir-374b.MIMAT0004956	0.144505	0.203872	0.642096
hsa-mir-454.MIMAT0003884	−0.14387	0.205889	0.642096
hsa-mir-541.MIMAT0004919	−0.14363	0.20666	0.642096
hsa-mir-548a-3.MIMAT0004803	−0.14353	0.206965	0.642096
hsa-mir-625.MIMAT0003294	0.14276	0.209443	0.642096
hsa-mir-629.MIMAT0004810	0.143852	0.205945	0.642096
hsa-mir-934.MIMAT0004977	−0.14377	0.206211	0.642096
hsa-mir-365.MIMAT0000710	−0.14175	0.212719	0.646072
hsa-mir-451.MIMAT0001631	0.14178	0.212621	0.646072
hsa-mir-628.MIMAT0003297	−0.13993	0.218726	0.661243
hsa-mir-148a.MIMAT0004549	−0.13952	0.220091	0.6623
hsa-mir-760.MIMAT0004957	0.1392	0.221147	0.662426
hsa-let-7c.MIMAT0004483	−0.13559	0.233469	0.672254
hsa-mir-106a.MIMAT0004517	0.137392	0.227264	0.672254
hsa-mir-129.MIMAT0000242	−0.13791	0.225486	0.672254
hsa-mir-138-1.MIMAT0004607	−0.13697	0.228694	0.672254

hsa-mir-202.MIMAT0002811	−0.13547	0.233884	0.672254
hsa-mir-204.MIMAT0000265	−0.13577	0.232841	0.672254
hsa-mir-30a.MIMAT0000087	−0.13722	0.227855	0.672254
hsa-mir-361.MIMAT0004682	0.136233	0.231245	0.672254
hsa-mir-509.MIMAT0002881	0.135232	0.234723	0.672254
hsa-mir-664.MIMAT0005949	0.136114	0.231657	0.672254
hsa-mir-1185.MIMAT0005798	−0.13423	0.238243	0.676402
hsa-mir-338.MIMAT0004701	−0.13442	0.237577	0.676402
hsa-let-7f-2.MIMAT0004487	0.132257	0.245276	0.677437
hsa-mir-126.MIMAT0000444	0.132651	0.243858	0.677437
hsa-mir-145.MIMAT0004601	−0.13322	0.241831	0.677437
hsa-mir-301a.MIMAT0000688	−0.13181	0.246907	0.677437
hsa-mir-365-2.MIMAT0009199	−0.13353	0.240709	0.677437
hsa-mir-539.MIMAT0003163	−0.13306	0.242388	0.677437
hsa-mir-708.MIMAT0004926	−0.13193	0.24646	0.677437
hsa-mir-873.MIMAT0004953	−0.13211	0.24581	0.677437
hsa-mir-7.MIMAT0000252	−0.13151	0.247979	0.677531
hsa-mir-18a.MIMAT0000072	−0.13067	0.251033	0.679263
hsa-mir-192.MIMAT0000222	0.130787	0.250607	0.679263
hsa-mir-33b.MIMAT0004811	0.13048	0.251733	0.679263
hsa-mir-146a.MIMAT0000449	0.127517	0.262753	0.680863
hsa-mir-20a.MIMAT0000075	0.129395	0.255728	0.680863
hsa-mir-20b.MIMAT0001413	0.128621	0.258608	0.680863
hsa-mir-432.MIMAT0002814	−0.12752	0.262723	0.680863
hsa-mir-433.MIMAT0001627	−0.12907	0.25692	0.680863
hsa-mir-483.MIMAT0004761	0.129524	0.255252	0.680863
hsa-mir-543.MIMAT0004954	−0.12854	0.258916	0.680863
hsa-mir-624.MIMAT0004807	0.129285	0.256138	0.680863
hsa-mir-664.MIMAT0005948	0.128201	0.260181	0.680863
hsa-mir-876.MIMAT0004925	−0.12768	0.262151	0.680863
hsa-mir-346.MIMAT0000773	−0.12648	0.266697	0.688353
hsa-mir-1226.MIMAT0005576	−0.12499	0.272413	0.697592
hsa-mir-548l.MIMAT0005889	0.125198	0.271603	0.697592
hsa-mir-301b.MIMAT0004958	−0.12436	0.274854	0.701092
hsa-mir-429.MIMAT0001536	−0.12357	0.277943	0.706214
hsa-mir-101.MIMAT0000099	0.119638	0.293627	0.712438
hsa-mir-10b.MIMAT0000254	−0.11796	0.300509	0.712438
hsa-mir-1180.MIMAT0005825	−0.12156	0.285905	0.712438
hsa-mir-1256.MIMAT0005907	−0.1176	0.302005	0.712438
hsa-mir-1306.MIMAT0005950	−0.12056	0.289916	0.712438
hsa-mir-137.MIMAT0000429	0.119525	0.294087	0.712438
hsa-mir-139.MIMAT0004552	−0.11755	0.302213	0.712438
hsa-mir-18b.MIMAT0001412	−0.121	0.288113	0.712438
hsa-mir-1909.MIMAT0007883	0.119123	0.295726	0.712438
hsa-mir-197.MIMAT0000227	−0.12022	0.291285	0.712438

hsa-mir-29a.MIMAT0000086	0.119203	0.295401	0.712438
hsa-mir-32.MIMAT0000090	0.119694	0.293401	0.712438
hsa-mir-33a.MIMAT0000091	−0.11773	0.301453	0.712438
hsa-mir-380.MIMAT0000735	−0.1193	0.294995	0.712438
hsa-mir-423.MIMAT0001340	0.11763	0.301863	0.712438
hsa-mir-513b.MIMAT0005788	0.122346	0.282759	0.712438
hsa-mir-548j.MIMAT0005875	0.118097	0.299935	0.712438
hsa-mir-581.MIMAT0003246	−0.1201	0.291757	0.712438
hsa-mir-610.MIMAT0003278	0.119103	0.295809	0.712438
hsa-mir-744.MIMAT0004946	−0.12124	0.287179	0.712438
hsa-mir-23b.MIMAT0000418	−0.11519	0.312088	0.73307
hsa-mir-145.MIMAT0000437	−0.11334	0.319963	0.7447
hsa-mir-200b.MIMAT0004571	−0.11318	0.32065	0.7447
hsa-mir-411.MIMAT0003329	−0.11296	0.321601	0.7447
hsa-mir-592.MIMAT0003260	−0.11362	0.318774	0.7447
hsa-mir-148b.MIMAT0004699	0.112187	0.324941	0.749776
hsa-mir-182.MIMAT0000260	−0.1109	0.330545	0.751457
hsa-mir-218-2.MIMAT0004566	−0.11064	0.331704	0.751457
hsa-mir-31.MIMAT0000089	0.110441	0.332574	0.751457
hsa-mir-654.MIMAT0003330	−0.11067	0.331581	0.751457
hsa-mir-663.MIMAT0003326	−0.11066	0.331613	0.751457
hsa-mir-767.MIMAT0003883	0.1112	0.329242	0.751457
hsa-mir-1915.MIMAT0007891	0.110113	0.334022	0.752125
hsa-mir-1287.MIMAT0005878	0.106954	0.348156	0.760354
hsa-mir-136.MIMAT0000448	−0.10809	0.343049	0.760354
hsa-mir-27b.MIMAT0000419	0.108995	0.338979	0.760354
hsa-mir-29b-1.MIMAT0004514	0.108015	0.343367	0.760354
hsa-mir-324.MIMAT0000762	−0.10758	0.345309	0.760354
hsa-mir-335.MIMAT0004703	−0.10827	0.342226	0.760354
hsa-mir-34b.MIMAT0000685	0.107922	0.343784	0.760354
hsa-mir-369.MIMAT0001621	−0.10714	0.347308	0.760354
hsa-mir-943.MIMAT0004986	−0.107	0.347926	0.760354
hsa-mir-143.MIMAT0000435	−0.10569	0.353898	0.765218
hsa-mir-28.MIMAT0000085	0.106059	0.352229	0.765218
hsa-mir-653.MIMAT0003328	−0.10589	0.352984	0.765218
hsa-mir-1284.MIMAT0005941	−0.10518	0.356263	0.766924
hsa-mir-19b.MIMAT0000074	0.10501	0.357037	0.766924
hsa-mir-181a-1.MIMAT0000270	−0.10443	0.359704	0.770121
hsa-mir-105.MIMAT0000102	−0.09528	0.403579	0.782009
hsa-mir-1204.MIMAT0005868	−0.09573	0.40134	0.782009
hsa-mir-1227.MIMAT0005580	−0.10087	0.376402	0.782009
hsa-mir-124.MIMAT0004591	0.10094	0.376081	0.782009
hsa-mir-1298.MIMAT0005800	−0.09876	0.386537	0.782009
hsa-mir-142.MIMAT0000433	0.095931	0.400346	0.782009
hsa-mir-154.MIMAT0000452	−0.09848	0.387884	0.782009

hsa-mir-199a.MIMAT0000231	−0.09955	0.382725	0.782009
hsa-mir-328.MIMAT0000752	−0.09702	0.394983	0.782009
hsa-mir-330.MIMAT0004693	−0.10195	0.371306	0.782009
hsa-mir-335.MIMAT0000765	−0.09853	0.387651	0.782009
hsa-mir-382.MIMAT0000737	−0.09607	0.399647	0.782009
hsa-mir-409.MIMAT0001639	−0.09694	0.395366	0.782009
hsa-mir-505.MIMAT0004776	0.096746	0.396338	0.782009
hsa-mir-512.MIMAT0002823	−0.09528	0.403579	0.782009
hsa-mir-516b.MIMAT0002859	−0.09528	0.403579	0.782009
hsa-mir-517c.MIMAT0002866	−0.09528	0.403579	0.782009
hsa-mir-518a.MIMAT0005457	−0.09528	0.403579	0.782009
hsa-mir-518c.MIMAT0002847	−0.09528	0.403579	0.782009
hsa-mir-518e.MIMAT0002861	−0.09528	0.403579	0.782009
hsa-mir-518f.MIMAT0002842	−0.09528	0.403579	0.782009
hsa-mir-519a-1.MIMAT0005452	−0.09528	0.403579	0.782009
hsa-mir-519a.MIMAT0002869	−0.09528	0.403579	0.782009
hsa-mir-519d.MIMAT0002853	−0.09528	0.403579	0.782009
hsa-mir-520a.MIMAT0002834	−0.09528	0.403579	0.782009
hsa-mir-520d.MIMAT0002856	−0.09528	0.403579	0.782009
hsa-mir-522.MIMAT0002868	−0.09528	0.403579	0.782009
hsa-mir-526b.MIMAT0002835	−0.09528	0.403579	0.782009
hsa-mir-527.MIMAT0002862	−0.09528	0.403579	0.782009
hsa-mir-891a.MIMAT0004902	−0.09972	0.381913	0.782009
hsa-mir-98.MIMAT0000096	−0.10044	0.378466	0.782009
hsa-mir-99a.MIMAT0004511	0.095938	0.400311	0.782009
hsa-mir-214.MIMAT0004564	−0.09478	0.406054	0.784476
hsa-mir-598.MIMAT0003266	−0.09427	0.408602	0.787071
hsa-mir-100.MIMAT0004512	0.0895	0.432809	0.792407
hsa-mir-1236.MIMAT0005591	−0.09173	0.421401	0.792407
hsa-mir-1247.MIMAT0005899	0.090265	0.428873	0.792407
hsa-mir-1277.MIMAT0005933	−0.08739	0.443788	0.792407
hsa-mir-1285.MIMAT0005876	−0.08933	0.433691	0.792407
hsa-mir-1286.MIMAT0005877	0.087127	0.445157	0.792407
hsa-mir-129-2.MIMAT0004605	0.086886	0.446422	0.792407
hsa-mir-1291.MIMAT0005881	0.092432	0.417827	0.792407
hsa-mir-134.MIMAT0000447	−0.08972	0.431688	0.792407
hsa-mir-182.MIMAT0000259	−0.09204	0.419793	0.792407
hsa-mir-191.MIMAT0001618	−0.08948	0.432899	0.792407
hsa-mir-214.MIMAT0000271	−0.09127	0.423733	0.792407
hsa-mir-223.MIMAT0004570	−0.09061	0.427127	0.792407
hsa-mir-28.MIMAT0004502	0.092193	0.419039	0.792407
hsa-mir-299.MIMAT0000687	−0.08992	0.430654	0.792407
hsa-mir-29b-2.MIMAT0004515	0.088325	0.4389	0.792407
hsa-mir-29c.MIMAT0000681	0.090166	0.429379	0.792407
hsa-mir-34c.MIMAT0000686	−0.08891	0.435859	0.792407

hsa-mir-363.MIMAT0003385	0.090324	0.428571	0.792407
hsa-mir-421.MIMAT0003339	−0.08686	0.446563	0.792407
hsa-mir-485.MIMAT0002176	−0.0871	0.445295	0.792407
hsa-mir-497.MIMAT0002820	−0.08978	0.431353	0.792407
hsa-mir-499.MIMAT0002870	−0.08787	0.441297	0.792407
hsa-mir-551b.MIMAT0004794	0.093147	0.414218	0.792407
hsa-mir-556.MIMAT0004793	−0.09336	0.413135	0.792407
hsa-mir-564.MIMAT0003228	−0.0889	0.435898	0.792407
hsa-mir-606.MIMAT0003274	−0.08712	0.445184	0.792407
hsa-mir-658.MIMAT0003336	−0.09188	0.420604	0.792407
hsa-mir-93.MIMAT0004509	0.087402	0.443716	0.792407
hsa-mir-1292.MIMAT0005943	−0.08639	0.449044	0.793062
hsa-mir-143.MIMAT0004599	−0.08633	0.449362	0.793062
hsa-mir-1911.MIMAT0007885	−0.08581	0.452073	0.79455
hsa-mir-29b.MIMAT0000100	0.085707	0.452638	0.79455
hsa-mir-138-2.MIMAT0004596	−0.08434	0.459908	0.805147
hsa-mir-363.MIMAT0000707	0.083508	0.464361	0.807334
hsa-mir-379.MIMAT0004690	−0.08318	0.466103	0.807334
hsa-mir-495.MIMAT0002817	−0.08324	0.465795	0.807334
hsa-mir-802.MIMAT0004185	−0.08351	0.464337	0.807334
hsa-mir-520g.MIMAT0002858	−0.08251	0.469725	0.811457
hsa-mir-1257.MIMAT0005908	0.078564	0.491311	0.812245
hsa-mir-127.MIMAT0004604	−0.08056	0.480329	0.812245
hsa-mir-129-1.MIMAT0004548	0.077664	0.496303	0.812245
hsa-mir-1301.MIMAT0005797	0.079934	0.483761	0.812245
hsa-mir-138.MIMAT0000430	−0.07799	0.494501	0.812245
hsa-mir-144.MIMAT0000436	−0.07981	0.484459	0.812245
hsa-mir-1910.MIMAT0007884	0.081306	0.476264	0.812245
hsa-mir-218-1.MIMAT0004565	−0.0788	0.490009	0.812245
hsa-mir-24-1.MIMAT0000079	−0.07811	0.49382	0.812245
hsa-mir-338.MIMAT0000763	−0.07768	0.496206	0.812245
hsa-mir-340.MIMAT0000750	−0.07889	0.489492	0.812245
hsa-mir-490.MIMAT0004764	0.078564	0.491311	0.812245
hsa-mir-497.MIMAT0004768	−0.08103	0.477747	0.812245
hsa-mir-511.MIMAT0002808	−0.08036	0.481421	0.812245
hsa-mir-518c.MIMAT0002848	−0.08042	0.481091	0.812245
hsa-mir-520h.MIMAT0002867	−0.07792	0.494875	0.812245
hsa-mir-532.MIMAT0004780	−0.08044	0.480976	0.812245
hsa-mir-548b.MIMAT0003254	−0.07806	0.494092	0.812245
hsa-mir-612.MIMAT0003280	−0.08101	0.477874	0.812245
hsa-mir-671.MIMAT0003880	0.080443	0.480973	0.812245
hsa-mir-887.MIMAT0004951	−0.07789	0.495026	0.812245
hsa-mir-1262.MIMAT0005914	−0.07558	0.507979	0.813015
hsa-mir-1269.MIMAT0005923	−0.07689	0.500603	0.813015
hsa-mir-1304.MIMAT0005892	0.075687	0.507363	0.813015

hsa-mir-148a.MIMAT0000243	−0.07665	0.501958	0.813015
hsa-mir-1914.MIMAT0007890	0.076383	0.503454	0.813015
hsa-mir-373.MIMAT0000726	0.076045	0.50535	0.813015
hsa-mir-377.MIMAT0004689	0.076822	0.500998	0.813015
hsa-mir-493.MIMAT0002813	−0.07701	0.499943	0.813015
hsa-mir-92b.MIMAT0004792	−0.07577	0.506905	0.813015
hsa-mir-106a.MIMAT0000103	0.074054	0.516593	0.814751
hsa-mir-1181.MIMAT0005826	−0.07371	0.518572	0.814751
hsa-mir-127.MIMAT0000446	−0.07369	0.518682	0.814751
hsa-mir-337.MIMAT0004695	−0.07362	0.519045	0.814751
hsa-mir-34b.MIMAT0004676	−0.07454	0.513843	0.814751
hsa-mir-379.MIMAT0000733	−0.0749	0.511809	0.814751
hsa-mir-505.MIMAT0002876	0.073716	0.518515	0.814751
hsa-mir-654.MIMAT0004814	−0.07423	0.515583	0.814751
hsa-mir-410.MIMAT0002171	−0.07243	0.525843	0.823442
hsa-mir-369.MIMAT0000721	−0.07196	0.528544	0.823721
hsa-mir-381.MIMAT0000736	−0.07218	0.527307	0.823721
hsa-mir-1276.MIMAT0005930	−0.07152	0.531082	0.825098
hsa-mir-17.MIMAT0000070	0.071149	0.533218	0.825098
hsa-mir-30c.MIMAT0000244	0.071314	0.532268	0.825098
hsa-mir-1229.MIMAT0005584	0.069595	0.542225	0.825223
hsa-mir-151.MIMAT0000757	0.070351	0.537836	0.825223
hsa-mir-186.MIMAT0004612	0.069809	0.540981	0.825223
hsa-mir-302d.MIMAT0000718	−0.07011	0.539236	0.825223
hsa-mir-487b.MIMAT0003180	−0.06947	0.542965	0.825223
hsa-mir-542.MIMAT0003389	−0.06955	0.542471	0.825223
hsa-mir-769.MIMAT0003886	0.069391	0.543409	0.825223
hsa-mir-92b.MIMAT0003218	0.070831	0.535058	0.825223
hsa-mir-425.MIMAT0001343	−0.06911	0.545069	0.825824
hsa-mir-136.MIMAT0004606	−0.06777	0.552905	0.826194
hsa-mir-141.MIMAT0004598	−0.06844	0.548936	0.826194
hsa-mir-23b.MIMAT0004587	0.067899	0.552134	0.826194
hsa-mir-337.MIMAT0000754	−0.06876	0.547113	0.826194
hsa-mir-517a.MIMAT0002852	−0.06811	0.550918	0.826194
hsa-mir-517b.MIMAT0002857	−0.06811	0.550918	0.826194
hsa-let-7a-2.MIMAT0010195	0.067133	0.556639	0.828445
hsa-mir-25.MIMAT0004498	0.06708	0.556949	0.828445
hsa-mir-100.MIMAT0000098	0.066586	0.559865	0.830891
hsa-mir-375.MIMAT0000728	−0.0662	0.562171	0.83242
hsa-mir-19a.MIMAT0000073	0.065731	0.564924	0.832721
hsa-mir-380.MIMAT0000734	−0.06583	0.564346	0.832721
hsa-mir-485.MIMAT0002175	−0.06505	0.568956	0.836776
hsa-mir-140.MIMAT0000431	−0.06368	0.577179	0.84129
hsa-mir-146a.MIMAT0004608	0.063696	0.577058	0.84129
hsa-mir-579.MIMAT0003244	0.063738	0.576807	0.84129

hsa-mir-675.MIMAT0006790	0.064123	0.574503	0.84129
hsa-mir-135b.MIMAT0004698	−0.06299	0.581306	0.845418
hsa-let-7i.MIMAT0004585	0.061735	0.588862	0.851369
hsa-mir-181c.MIMAT0000258	0.06104	0.593071	0.851369
hsa-mir-200b.MIMAT0000318	−0.06115	0.592387	0.851369
hsa-mir-323.MIMAT0000755	−0.06136	0.591115	0.851369
hsa-mir-33b.MIMAT0003301	−0.06107	0.592896	0.851369
hsa-mir-499.MIMAT0004772	−0.06081	0.594441	0.851369
hsa-mir-573.MIMAT0003238	0.060801	0.594524	0.851369
hsa-mir-200a.MIMAT0001620	−0.06044	0.596716	0.852638
hsa-mir-181a-2.MIMAT0004558	0.06017	0.598363	0.853124
hsa-mir-889.MIMAT0004921	−0.05969	0.601291	0.855431
hsa-mir-665.MIMAT0004952	−0.05911	0.604815	0.858574
hsa-mir-770.MIMAT0003948	0.058811	0.606669	0.859338
hsa-mir-339.MIMAT0000764	−0.05845	0.608869	0.859797
hsa-mir-33a.MIMAT0004506	−0.05833	0.609626	0.859797
hsa-mir-452.MIMAT0001636	−0.05698	0.617945	0.866314
hsa-mir-766.MIMAT0003888	−0.05693	0.618227	0.866314
hsa-mir-96.MIMAT0004510	−0.0573	0.615958	0.866314
hsa-mir-125b.MIMAT0000423	0.05526	0.628595	0.876999
hsa-mir-590.MIMAT0004801	0.055054	0.629882	0.876999
hsa-mir-942.MIMAT0004985	0.055344	0.628073	0.876999
hsa-let-7i.MIMAT0000415	0.053545	0.639308	0.879779
hsa-mir-128.MIMAT0000424	−0.05259	0.645285	0.879779
hsa-mir-1293.MIMAT0005883	−0.05269	0.644664	0.879779
hsa-mir-1912.MIMAT0007887	−0.05221	0.647673	0.879779
hsa-mir-196a-2.MIMAT0004562	0.053979	0.636591	0.879779
hsa-mir-196a.MIMAT0000226	0.051667	0.651121	0.879779
hsa-mir-376c.MIMAT0000720	−0.05352	0.639487	0.879779
hsa-mir-409.MIMAT0001638	0.05381	0.637646	0.879779
hsa-mir-449b.MIMAT0009203	−0.05245	0.646178	0.879779
hsa-mir-541.MIMAT0004920	−0.05208	0.648542	0.879779
hsa-mir-597.MIMAT0003265	−0.05357	0.639167	0.879779
hsa-mir-617.MIMAT0003286	−0.05189	0.649712	0.879779
hsa-mir-618.MIMAT0003287	0.051815	0.650187	0.879779
hsa-mir-933.MIMAT0004976	−0.05221	0.647671	0.879779
hsa-mir-944.MIMAT0004987	0.051514	0.652087	0.879779
hsa-mir-1909.MIMAT0007882	−0.05064	0.657612	0.883581
hsa-mir-577.MIMAT0003242	−0.05065	0.657576	0.883581
hsa-mir-181c.MIMAT0004559	0.049991	0.661735	0.885477
hsa-mir-639.MIMAT0003309	−0.05006	0.661311	0.885477
hsa-mir-486.MIMAT0002177	−0.04935	0.66581	0.887294
hsa-mir-99a.MIMAT0000097	0.049371	0.66568	0.887294
hsa-mir-494.MIMAT0002816	0.048453	0.671536	0.893101
hsa-mir-19a.MIMAT0004490	0.047971	0.674618	0.89445

hsa-mir-32.MIMAT0004505	−0.04787	0.675289	0.89445
hsa-mir-296.MIMAT0004679	−0.04715	0.679877	0.898704
hsa-mir-191.MIMAT0000440	0.046567	0.683623	0.90173
hsa-mir-205.MIMAT0000266	−0.04579	0.688602	0.90173
hsa-mir-370.MIMAT0000722	−0.04567	0.689385	0.90173
hsa-mir-376a-1.MIMAT0003386	0.045151	0.69276	0.90173
hsa-mir-455.MIMAT0003150	0.046155	0.68628	0.90173
hsa-mir-590.MIMAT0003258	−0.04487	0.694594	0.90173
hsa-mir-605.MIMAT0003273	−0.04519	0.692497	0.90173
hsa-mir-660.MIMAT0003338	−0.04498	0.693838	0.90173
hsa-mir-939.MIMAT0004982	0.045027	0.693562	0.90173
hsa-mir-1537.MIMAT0007399	−0.04406	0.699818	0.901971
hsa-mir-195.MIMAT0000461	−0.04358	0.702939	0.901971
hsa-mir-331.MIMAT0004700	−0.04356	0.703068	0.901971
hsa-mir-342.MIMAT0000753	−0.04368	0.702277	0.901971
hsa-mir-424.MIMAT0001341	0.04458	0.696452	0.901971
hsa-mir-518b.MIMAT0002844	−0.0436	0.702784	0.901971
hsa-mir-671.MIMAT0004819	−0.04297	0.706921	0.905136
hsa-let-7d.MIMAT0004484	0.042688	0.708743	0.905657
hsa-mir-1182.MIMAT0005827	0.041843	0.714257	0.905657
hsa-mir-183.MIMAT0004560	−0.0419	0.713873	0.905657
hsa-mir-627.MIMAT0003296	0.042204	0.711899	0.905657
hsa-mir-938.MIMAT0004981	−0.04184	0.714263	0.905657
hsa-mir-1258.MIMAT0005909	−0.04094	0.720139	0.908269
hsa-mir-150.MIMAT0004610	−0.03962	0.728841	0.908269
hsa-mir-154.MIMAT0000453	−0.03972	0.728161	0.908269
hsa-mir-203.MIMAT0000264	0.03999	0.726405	0.908269
hsa-mir-452.MIMAT0001635	−0.04061	0.722344	0.908269
hsa-mir-542.MIMAT0003340	−0.04012	0.725545	0.908269
hsa-mir-545.MIMAT0004785	0.040645	0.722106	0.908269
hsa-mir-638.MIMAT0003308	−0.04007	0.725902	0.908269
hsa-mir-675.MIMAT0004284	−0.04049	0.72313	0.908269
hsa-mir-374a.MIMAT0004688	0.039363	0.730525	0.908634
hsa-mir-1973.MIMAT0009448	−0.03849	0.736286	0.914059
hsa-mir-1908.MIMAT0007881	−0.03809	0.73894	0.915613
hsa-mir-17.MIMAT0000071	−0.0378	0.740872	0.916268
hsa-mir-224.MIMAT0000281	−0.03718	0.744964	0.918027
hsa-mir-324.MIMAT0000761	−0.03694	0.746512	0.918027
hsa-mir-518a.MIMAT0002863	0.037049	0.745817	0.918027
hsa-mir-331.MIMAT0000760	−0.0364	0.750117	0.91968
hsa-mir-431.MIMAT0001625	0.036317	0.750673	0.91968
hsa-mir-122.MIMAT0000421	0.03606	0.752381	0.920047
hsa-mir-186.MIMAT0000456	0.034986	0.759528	0.921014
hsa-mir-34a.MIMAT0000255	−0.03498	0.759573	0.921014
hsa-mir-450a.MIMAT0001545	−0.0355	0.756124	0.921014

hsa-mir-504.MIMAT0002875	0.034882	0.760224	0.921014
hsa-mir-632.MIMAT0003302	−0.03495	0.759741	0.921014
hsa-mir-10b.MIMAT0004556	−0.03403	0.765935	0.925002
hsa-mir-206.MIMAT0000462	0.03354	0.769183	0.925002
hsa-mir-26b.MIMAT0000083	−0.03356	0.769032	0.925002
hsa-mir-491.MIMAT0004765	0.033595	0.768815	0.925002
hsa-mir-30e.MIMAT0000693	−0.03312	0.771996	0.926679
hsa-mir-18b.MIMAT0004751	0.032577	0.775633	0.929336
hsa-mir-224.MIMAT0009198	−0.03225	0.777859	0.930296
hsa-mir-9.MIMAT0000441	−0.03175	0.781158	0.932534
hsa-mir-148b.MIMAT0000759	0.031513	0.782781	0.932766
hsa-mir-125b-2.MIMAT0004603	0.031051	0.785889	0.933065
hsa-mir-30c-1.MIMAT0004674	0.031111	0.785483	0.933065
hsa-mir-20b.MIMAT0004752	0.029724	0.794831	0.941968
hsa-mir-588.MIMAT0003255	−0.02951	0.796278	0.941974
hsa-mir-147b.MIMAT0004928	−0.02878	0.801215	0.94279
hsa-mir-329.MIMAT0001629	−0.02874	0.801498	0.94279
hsa-mir-342.MIMAT0004694	−0.02878	0.801234	0.94279
hsa-mir-502.MIMAT0002873	−0.02855	0.802743	0.94279
hsa-mir-125b-1.MIMAT0004592	0.027995	0.806525	0.943513
hsa-mir-1280.MIMAT0005946	−0.02521	0.825454	0.943513
hsa-mir-1294.MIMAT0005884	0.026721	0.815173	0.943513
hsa-mir-193b.MIMAT0004767	−0.02528	0.825003	0.943513
hsa-mir-195.MIMAT0004615	−0.02558	0.822953	0.943513
hsa-mir-222.MIMAT0004569	0.02506	0.826477	0.943513
hsa-mir-26b.MIMAT0004500	−0.02635	0.817674	0.943513
hsa-mir-27a.MIMAT0004501	0.025679	0.822258	0.943513
hsa-mir-34a.MIMAT0004557	0.026578	0.816147	0.943513
hsa-mir-431.MIMAT0004757	−0.02655	0.816348	0.943513
hsa-mir-454.MIMAT0003885	0.025721	0.821972	0.943513
hsa-mir-455.MIMAT0004784	0.028106	0.805778	0.943513
hsa-mir-503.MIMAT0002874	−0.02603	0.81988	0.943513
hsa-mir-545.MIMAT0003165	−0.0265	0.816706	0.943513
hsa-mir-615.MIMAT0003283	0.025646	0.822481	0.943513
hsa-mir-96.MIMAT0000095	−0.02734	0.810969	0.943513
hsa-mir-488.MIMAT0004763	0.024512	0.830216	0.946127
hsa-mir-15a.MIMAT0004488	−0.024	0.833676	0.947623
hsa-mir-221.MIMAT0000278	0.023837	0.834821	0.947623
hsa-mir-483.MIMAT0002173	0.023682	0.835882	0.947623
hsa-let-7c.MIMAT0000064	0.023086	0.839955	0.95029
hsa-mir-150.MIMAT0000451	−0.02193	0.84785	0.95029
hsa-mir-153.MIMAT0000439	0.022088	0.846786	0.95029
hsa-mir-200a.MIMAT0000682	−0.02227	0.845521	0.95029
hsa-mir-376a.MIMAT0000729	0.021553	0.850456	0.95029
hsa-mir-449b.MIMAT0003327	−0.02157	0.850362	0.95029

hsa-mir-659.MIMAT0003337	−0.02143	0.851332	0.95029
hsa-mir-885.MIMAT0004948	−0.02157	0.850362	0.95029
hsa-mir-95.MIMAT0000094	0.021428	0.851315	0.95029
hsa-mir-219.MIMAT0000276	−0.02101	0.854174	0.951835
hsa-mir-432.MIMAT0002815	0.020561	0.857264	0.953652
hsa-mir-222.MIMAT0000279	0.020176	0.859913	0.954717
hsa-mir-411.MIMAT0004813	0.019996	0.861146	0.954717
hsa-mir-299.MIMAT0002890	−0.01868	0.870234	0.963157
hsa-mir-548e.MIMAT0005874	−0.01715	0.880756	0.973153
hsa-mir-93.MIMAT0000093	0.016603	0.884529	0.974026
hsa-mir-935.MIMAT0004978	−0.01672	0.883689	0.974026
hsa-mir-1278.MIMAT0005936	−0.01616	0.887557	0.974076
hsa-mir-548o.MIMAT0005919	−0.0163	0.886589	0.974076
hsa-mir-362.MIMAT0004683	−0.01542	0.892713	0.976043
hsa-mir-556.MIMAT0003220	−0.01526	0.893834	0.976043
hsa-mir-655.MIMAT0003331	−0.01562	0.891329	0.976043
hsa-mir-1288.MIMAT0005942	−0.01413	0.901659	0.976424
hsa-mir-142.MIMAT0000434	−0.0143	0.900443	0.976424
hsa-mir-199a.MIMAT0000232	−0.01429	0.900508	0.976424
hsa-mir-199b.MIMAT0004563	−0.01416	0.901444	0.976424
hsa-mir-383.MIMAT0000738	0.014782	0.89712	0.976424
hsa-mir-1246.MIMAT0005898	0.011506	0.919838	0.977214
hsa-mir-1260.MIMAT0005911	−0.01096	0.9236	0.977214
hsa-mir-15a.MIMAT0000068	0.012683	0.911664	0.977214
hsa-mir-181a.MIMAT0000256	−0.01128	0.921428	0.977214
hsa-mir-181b.MIMAT0000257	0.011905	0.917064	0.977214
hsa-mir-193b.MIMAT0002819	−0.01233	0.914083	0.977214
hsa-mir-339.MIMAT0004702	0.010893	0.924096	0.977214
hsa-mir-422a.MIMAT0001339	−0.01079	0.924836	0.977214
hsa-mir-450b.MIMAT0004909	−0.01251	0.912894	0.977214
hsa-mir-488.MIMAT0002804	−0.01087	0.924245	0.977214
hsa-mir-548a.MIMAT0003251	−0.01087	0.924245	0.977214
hsa-mir-552.MIMAT0003215	−0.01335	0.907017	0.977214
hsa-mir-580.MIMAT0003245	0.012841	0.910573	0.977214
hsa-mir-609.MIMAT0003277	−0.01079	0.924836	0.977214
hsa-mir-7-2.MIMAT0004554	−0.0129	0.910145	0.977214
hsa-mir-586.MIMAT0003252	0.010566	0.926369	0.977252
hsa-mir-1468.MIMAT0006789	0.009547	0.93345	0.983134
hsa-mir-377.MIMAT0000730	−0.00929	0.935265	0.983459
hsa-mir-199b.MIMAT0000263	0.008848	0.938313	0.983811
hsa-mir-210.MIMAT0000267	0.008805	0.938613	0.983811
hsa-mir-1237.MIMAT0005592	0.005956	0.958454	0.987454
hsa-mir-1238.MIMAT0005593	−0.00633	0.955854	0.987454
hsa-mir-124.MIMAT0000422	0.0057	0.960235	0.987454
hsa-mir-1248.MIMAT0005900	0.005861	0.959118	0.987454

hsa-mir-130b.MIMAT0004680	−0.00681	0.952532	0.987454
hsa-mir-149.MIMAT0004609	0.005731	0.960022	0.987454
hsa-mir-200c.MIMAT0004657	−0.0072	0.949818	0.987454
hsa-mir-202.MIMAT0002810	0.006659	0.953556	0.987454
hsa-mir-30e.MIMAT0000692	0.006965	0.951421	0.987454
hsa-mir-486.MIMAT0004762	−0.00637	0.955602	0.987454
hsa-mir-500.MIMAT0004773	0.006306	0.956012	0.987454
hsa-mir-652.MIMAT0003322	0.007424	0.948228	0.987454
hsa-mir-570.MIMAT0003235	−0.00521	0.963624	0.989381
hsa-mir-194-2.MIMAT0004671	0.003883	0.972907	0.99112
hsa-mir-23a.MIMAT0004496	−0.00433	0.969817	0.99112
hsa-mir-758.MIMAT0003879	0.003919	0.972659	0.99112
hsa-mir-769.MIMAT0003887	−0.00417	0.970882	0.99112
hsa-mir-9.MIMAT0000442	−0.00429	0.970094	0.99112
hsa-mir-1295.MIMAT0005885	−0.00309	0.978426	0.991859
hsa-mir-135b.MIMAT0000758	−0.00291	0.979708	0.991859
hsa-mir-19b-2.MIMAT0004492	−0.00333	0.976794	0.991859
hsa-mir-643.MIMAT0003313	0.003034	0.978829	0.991859
hsa-mir-16-1.MIMAT0004489	−0.00237	0.98343	0.99255
hsa-mir-875.MIMAT0004923	−0.00241	0.983198	0.99255
hsa-mir-487a.MIMAT0002178	0.001442	0.989934	0.997572
hsa-mir-1270.MIMAT0005924	−7.5E−05	0.999479	0.999933
hsa-mir-1296.MIMAT0005794	−0.0006	0.995807	0.999933
hsa-mir-187.MIMAT0004561	0.000397	0.997233	0.999933
hsa-mir-424.MIMAT0004749	−9.59E−06	0.999933	0.999933
hsa-mir-656.MIMAT0003332	0.000233	0.998373	0.999933

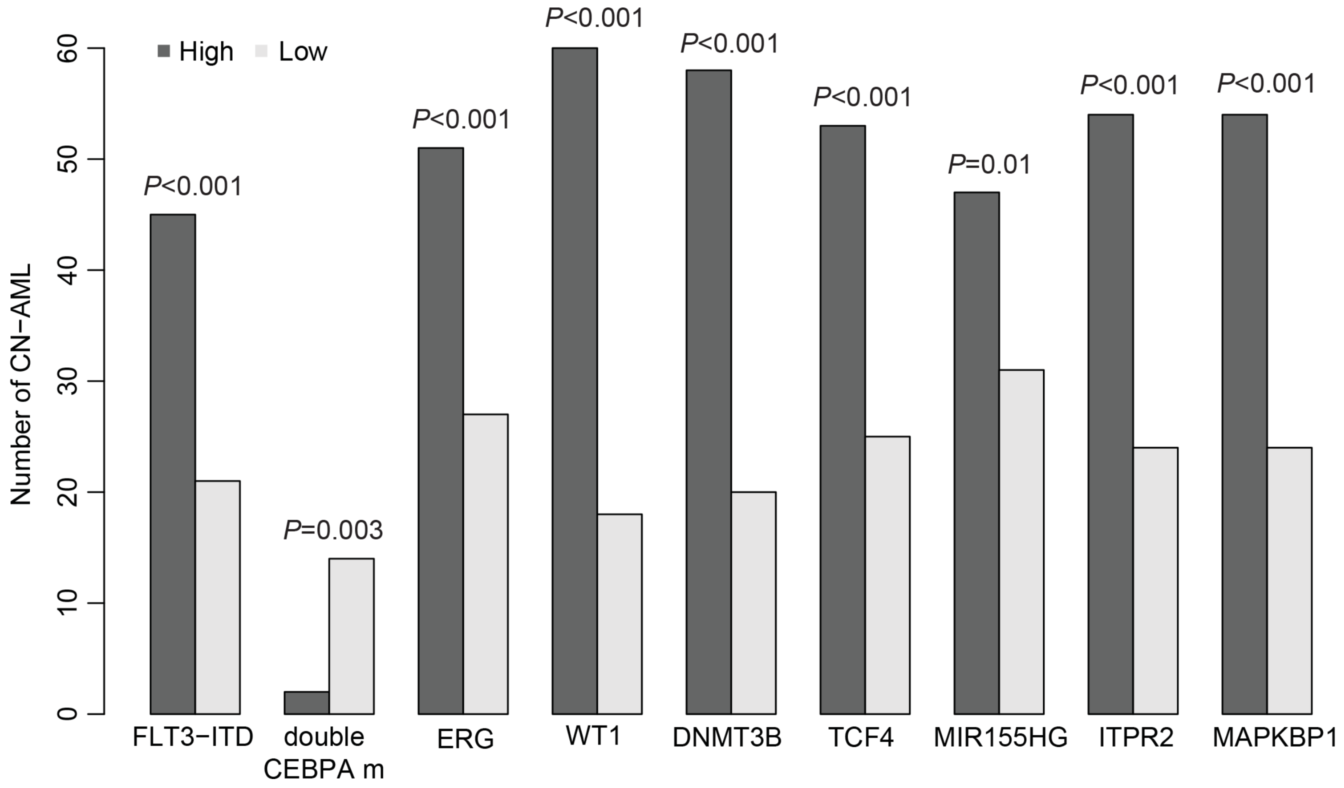

Supplementary Figure S1: Association between *RUNX1* expression and prognostic markers in CN-AML.

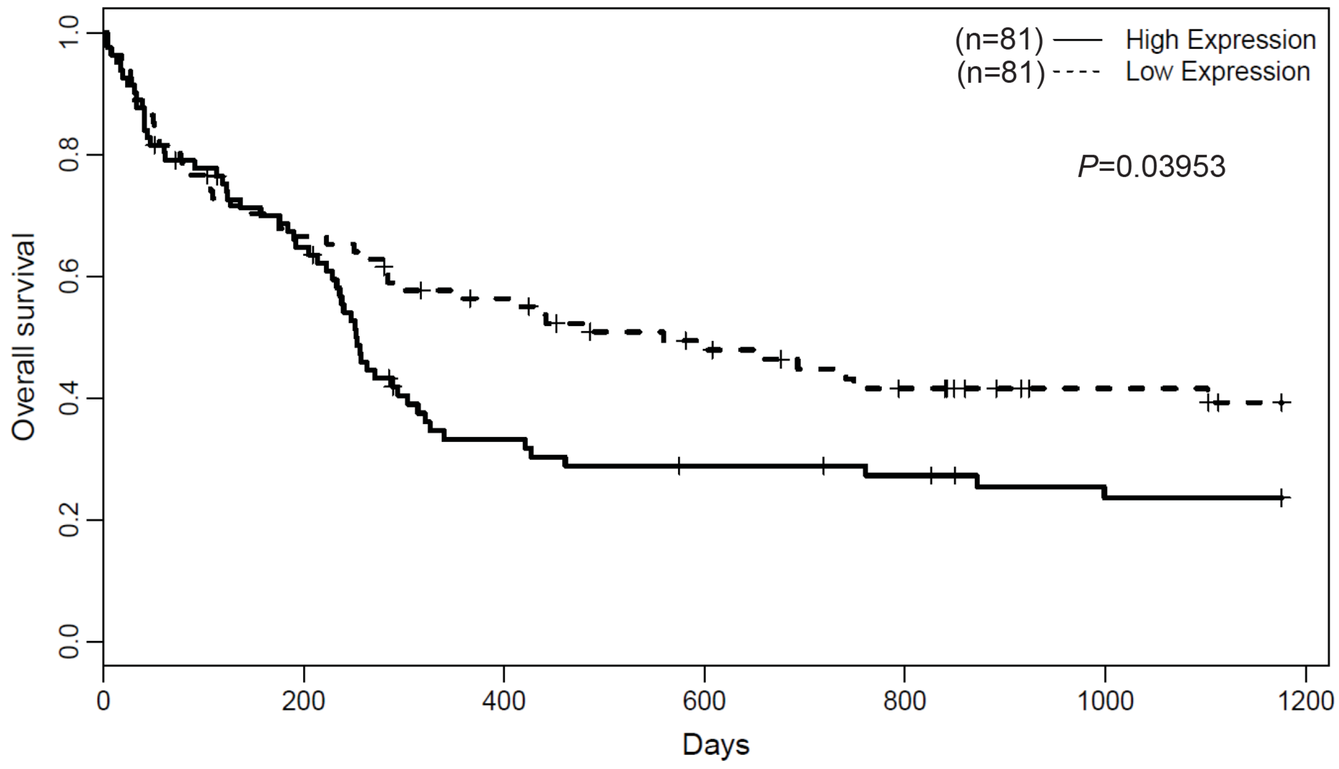

Supplementary Figure S2: OS of CN-AML patients in the validating group.

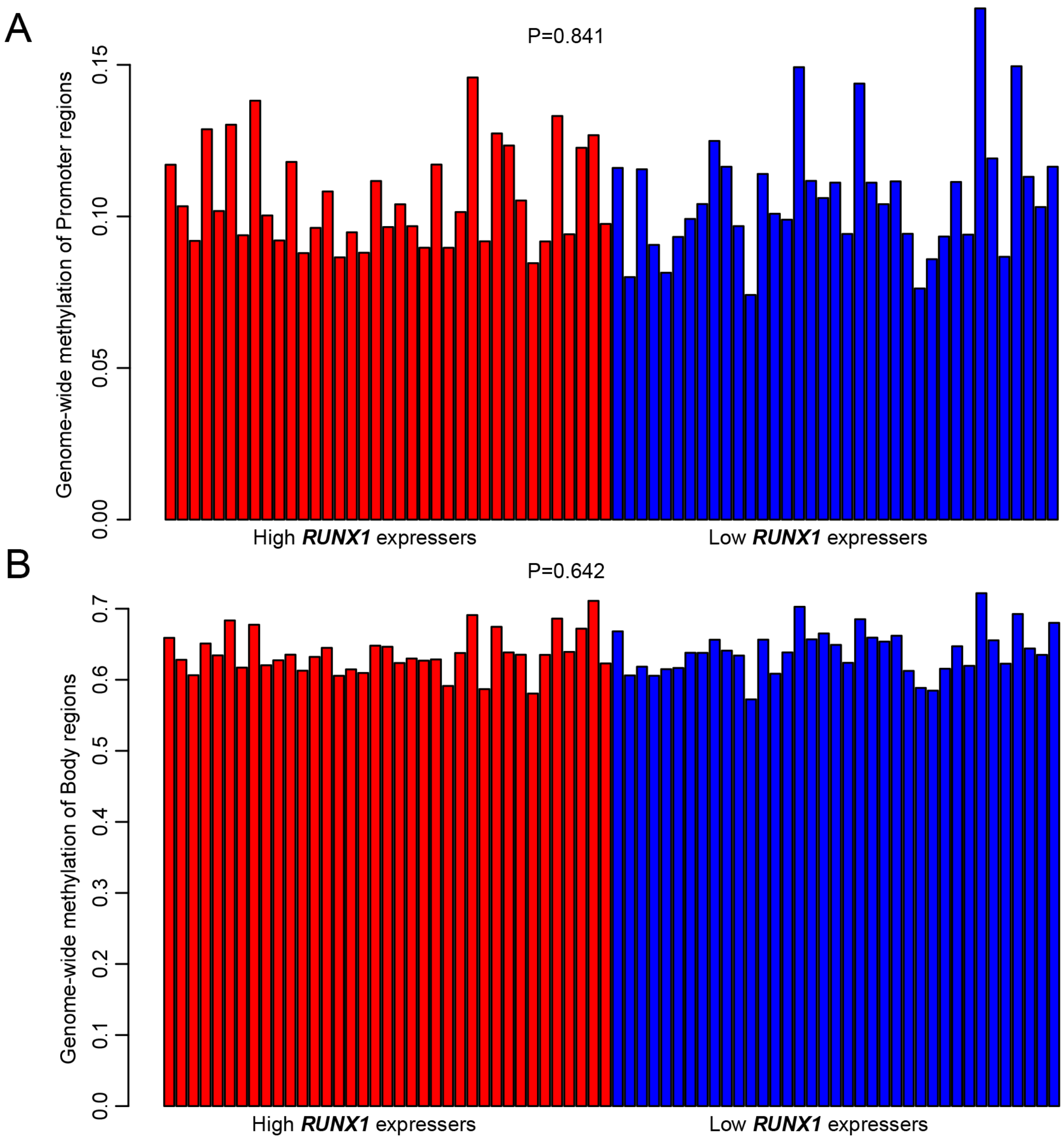

Supplementary Figure S3: Genome-wide methylation profiling associated with RUNX1 expression. (**A**) Gene promoter methylation profiling and (**B**) Gene body methylation profiling associated with RUNX1 expression.

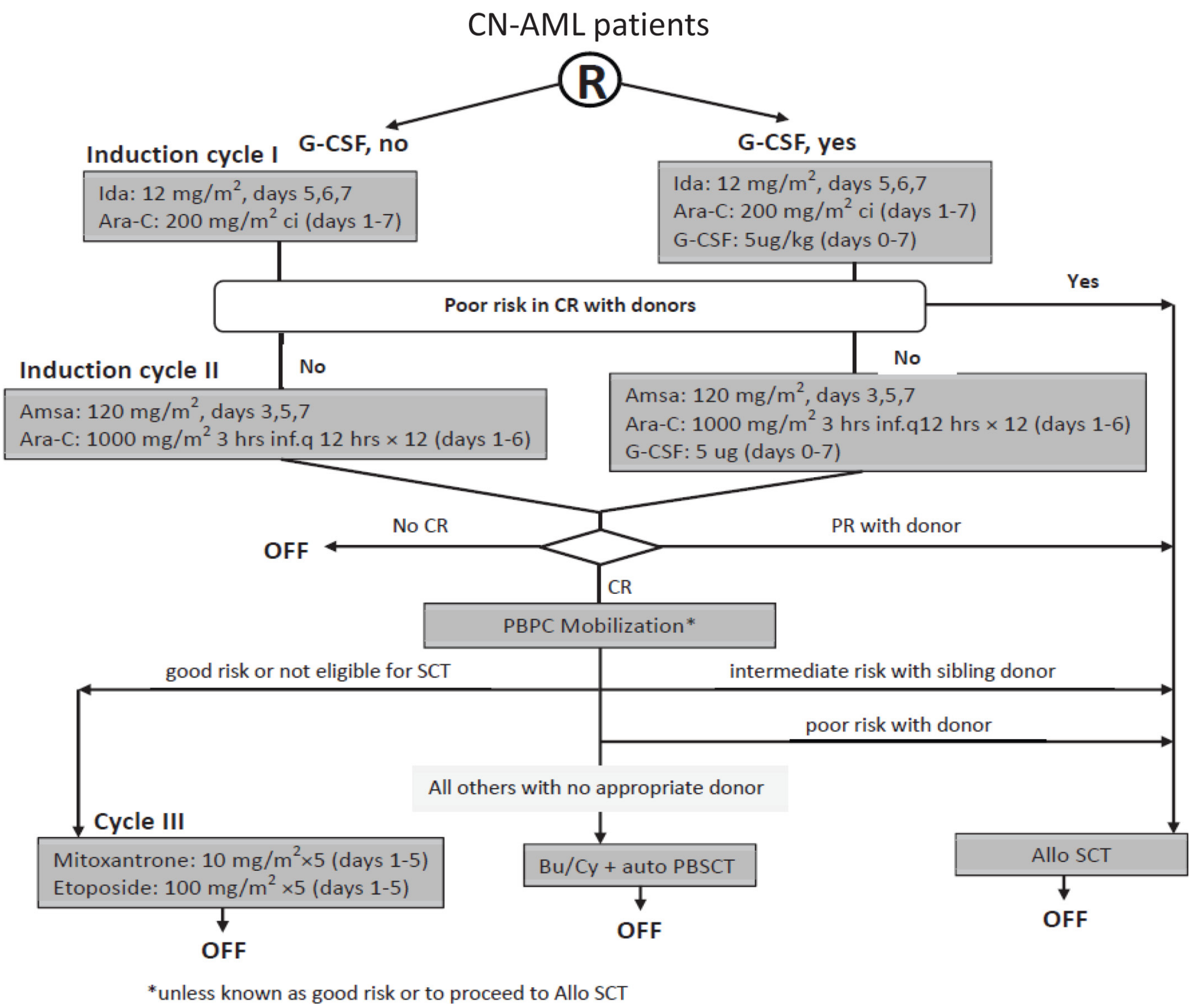

Supplementary Figure S4: Therapeutic strategies of CN-AML patients.

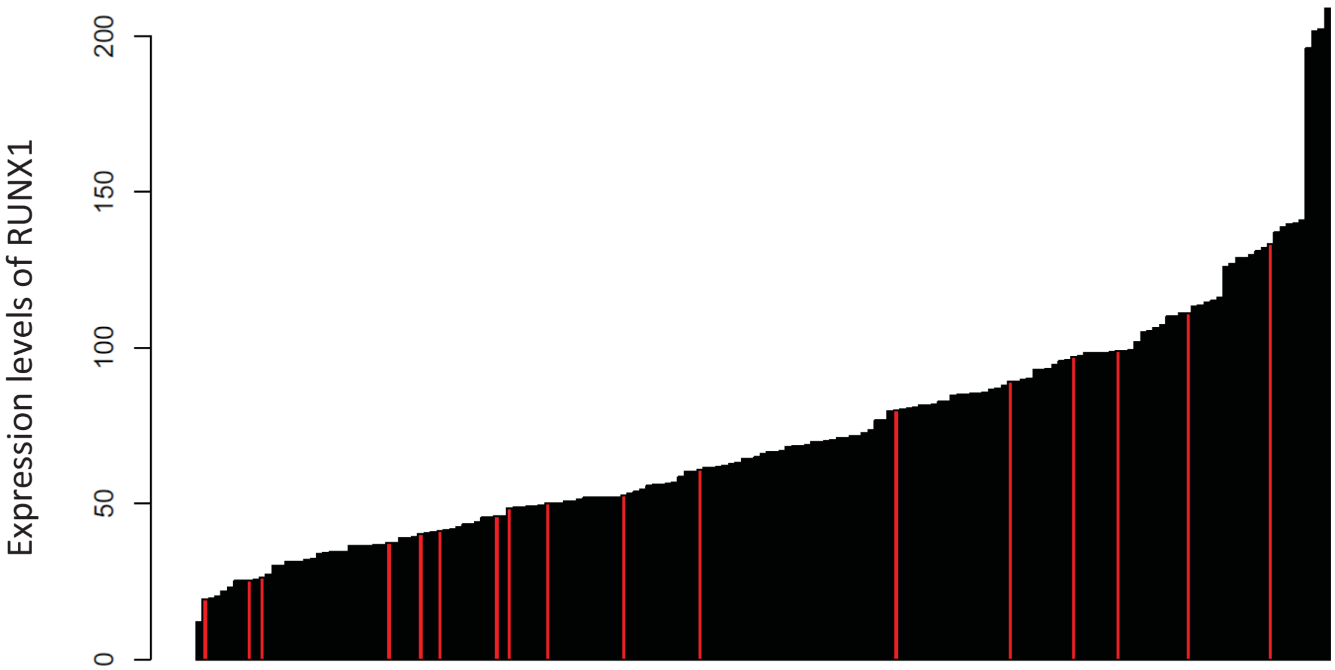

Supplementary Figure S5: ***RUNX1*** **expression is normally distributed.**
